# Regulated localization of transposable element RNA during influenza A virus infection

**DOI:** 10.1038/s44319-025-00498-2

**Published:** 2025-06-16

**Authors:** Marie Lork, Liam Childs, Gauthier Lieber, Florence Kwaschik, Renate König, Benjamin G Hale

**Affiliations:** 1 Institute of Medical Virology, University of Zurich, Zurich, 8057 Switzerland; 2 Host-Pathogen Interactions, Paul-Ehrlich-Institut, Langen, 63225 Germany

**Keywords:** dsRNA Sensors, Influenza A Virus, Transposable Elements, Viral Antagonism, Chromatin, Transcription & Genomics, Microbiology, Virology & Host Pathogen Interaction, Signal Transduction

## Abstract

Influenza A virus (IAV) infection triggers derepression of host transposable elements (TEs), which have the potential to form double-stranded (ds)RNAs and could enhance innate antiviral immunity. However, the contribution of TEs to stimulating such pathways during infection is unknown, and it remains unclear whether derepressed TEs actually form dsRNAs. Here, we perform strand-specific total RNA-Seq on nucleus-associated and cytosolic fractions from cells infected with wild-type IAV or an engineered IAV lacking NS1, a dsRNA-binding interferon-antagonist protein. Both infections globally increase levels of host TE RNAs with bioinformatic and experimental evidence for double-strandedness. However, NS1-deficient IAV leads to significantly more of these putative dsRNA-forming TEs accumulating in the cytosolic fraction. Co-precipitations identify that wild-type NS1, but not a dsRNA-binding mutant, associates with these TEs, and microscopy shows co-localization of wild-type NS1 with dsRNA in perinuclear regions. Our data reveal the double-stranded nature of some IAV-triggered host TEs and suggest that NS1-mediated sequestration could limit their engagement of cytosolic innate immune sensors.

## Synopsis

Influenza A virus infection increases host transposable element (TE) expression. This study provides evidence that some infection-induced TEs form double-stranded RNAs that accumulate in the cytosol in the absence of the viral interferon-antagonist protein NS1.

- Influenza A virus (IAV) infection induces host transposable element double-stranded RNAs (TE-dsRNAs).
- TE-dsRNAs are mainly observed in the nucleus, but accumulate in the cytosol in the absence of the IAV NS1 protein.
- IAV NS1 protein, via its RNA-binding domain, can physically associate with TE-dsRNAs.
- Viral sequestration of TE-dsRNAs may limit their immunostimulatory engagement with host antiviral cytosolic sensors.

## Abstract (web summary)

Influenza A virus infection increases host transposable element (TE) expression. This study provides evidence that some infection-induced TEs form double-stranded RNAs that accumulate in the cytosol in the absence of the viral interferon-antagonist protein NS1.

## Graphical abstract

**Figure:**
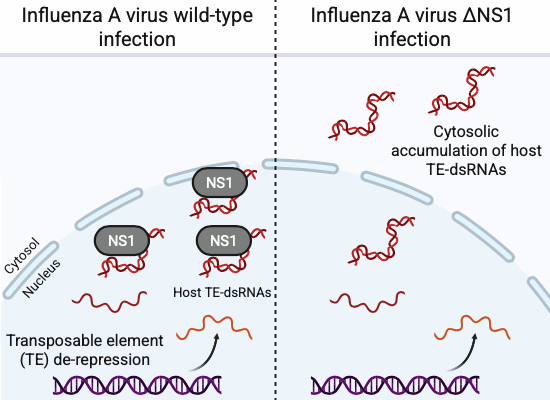

**Figure:**
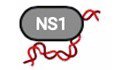

## Introduction

While only 1–2% of the human genome encodes proteins, the remaining repetitive, low-complexity part is poorly characterized, and was long referred to as “junk DNA” (ENCODE Project Consortium 2012; Lander et al, 2001). A substantial fraction thereof (~45%) is made up by transposable elements (TEs), DNA sequences that have the ability to move within a genome and can be classified based on their mechanism of transposition: DNA transposons move by a “cut and paste” mechanism while retrotransposons adopt a “copy and paste” mechanism using RNA as an intermediate (Luning Prak and Kazazian, 2000). Endogenous retroviruses (ERVs), a type of retrotransposon, stem from ancient retroviral insertions into germline cells and can feature two long terminal repeats (LTRs) flanking their proviral genome (Grundy et al, 2022). Aside from LTR-containing ERVs, the genome also harbors non-LTR retroelements, such as long interspersed elements (LINEs) and short interspersed elements (SINEs). TE retrotransposition is generally associated with genome instability due to target gene disruption, alternative splicing, and various epigenetic changes (Bhat et al, 2022). For example, it has been demonstrated that the global epigenetic dysregulation of TEs in some cancer cells, resulting in their expression, can have oncogenic effects in certain scenarios (Grundy et al, 2022). To avoid these undesirable effects, expression of TEs is usually repressed by epigenetic mechanisms such as DNA methylation and histone modification. One key silencing pathway involves Krüppel-associated box (KRAB) zinc-finger proteins (ZFPs), which bind to specific DNA sequences in LTR elements and, together with TRIM28 (KAP1), recruit the methyltransferase SETDB1 to the promoter regions of KRAB target genes, resulting in repression of specific TEs (Rowe et al, 2010; Turelli et al, 2014). More recently, the involvement of the Human Silencing Hub (HUSH) complex in the suppression of TEs, especially evolutionary young LINE1 elements, has been described (Robbez-Masson et al, 2018).

Intriguingly, TEs have gained considerable interest for their apparent co-option by cells as contributors to innate immune defenses (Hale, 2022). For example, TE LTRs can function as promoters or enhancers for nearby genes, thereby affecting gene regulation, including for genes involved in the innate immune response (Chuong et al, 2016; Srinivasachar Badarinarayan and Sauter, 2021). In addition, aberrant accumulation of derepressed RNA transcripts has been reported to be a source of highly immunogenic “self” dsRNA, that can be sensed by host pattern recognition receptors (PRRs) to induce an antiviral interferon (IFN) response (Chiappinelli et al, 2015; Cuellar et al, 2017; Roulois et al, 2015). Mechanistically, the production of immunostimulatory TE-dsRNAs might arise through various processes, including possible LTR-dependent bidirectional transcription, hairpin formation, or read-through transcription of head-to-head or tail-to-tail arranged elements (Sadeq et al, 2021). In this context, we recently identified a potential new type of physiological mechanism leading to enhanced activation of antiviral immunity in response to virus infection: an influenza A virus (IAV)-triggered “switch” in TRIM28 causes transcriptional derepression of host TE RNAs (mainly ERVs), which appear to potentiate a protective IFN response (Schmidt et al, 2019). Specifically, we could show that loss of SUMO-modified TRIM28 leads to the transcriptional upregulation of several TEs that may act in an immunostimulatory fashion to impair virus replication. These data not only indicated that TEs may have been co-opted to aid defense against exogenous pathogens, but that the mechanisms governing retroelement silencing are physiologically regulated by the cell. Notably, IAV is not the only type of pathogen described to upregulate various host retroelements during infection (Young et al, 2014). Recently published re-analyses of publicly available gene expression datasets showed that infection with a diverse range of viruses can trigger changes in TE expression (Chen et al, 2023; Macchietto et al, 2020), indicating that insights into molecular mechanisms leading to IAV-induced TE expression, and their consequences, could be broadly applicable to other viruses.

To date, it has only been suggested that IAV-triggered derepressed retroelements have the potential to promote antiviral immunity by forming immunostimulatory dsRNAs that might activate PRRs (Schmidt et al, 2019). Indeed, it was not formally shown that these TEs form dsRNA, or that they can exit the nucleus and be located in the cytosol, where many PRRs are based. In addition, it is still unclear whether IAV might antagonize this pathway downstream of TE derepression, as IAV is known to employ various countermeasures to limit host antiviral defenses (Hale et al, 2010). In this context, the non-structural protein 1 (NS1), is probably the most well-characterized of the IAV innate immune antagonist proteins (Ayllon and García-Sastre, 2015). NS1 contains a dsRNA-binding domain that is critical for suppressing IFN induction during IAV infection (Donelan et al, 2003), although the precise dsRNA target bound by NS1 remains unclear (Cheng et al, 2009; Hatada et al, 1992; Weber et al, 2006; Zhang et al, 2018).

To address some of these open questions, here we perform an in-depth transcriptomic analysis of nucleus-associated and cytosolic fractions obtained from cells infected with wild-type (wt) IAV or an engineered IAV lacking NS1 expression (ΔNS1). We confirm that the lack of NS1 leads to enhanced stimulation of host innate immune responses during infection, as characterized by the expression of cytokines, chemokines, and interferon-stimulated genes (ISGs). Employing a novel bioinformatics analysis pipeline, we further identify and quantify the expression of TEs in each cellular compartment during infection, focusing on TEs that exhibit overlapping sense and antisense transcripts and thus have the possibility to form dsRNA. We observe a significant increase in such potentially double-stranded TE RNAs (TE-dsRNAs) during infection with both viruses, an observation that we validate orthogonally using an antibody specific to dsRNA. Strikingly, our fractionation analysis reveals that TE-dsRNAs only predominantly accumulate in the cytosol following infection with IAV ΔNS1, suggesting a possible role for NS1 in restricting the efficient release of TE-dsRNAs from nucleus-associated structures. This hypothesis is supported by the finding that wt NS1, but not a dsRNA-binding deficient mutant, can co-precipitate TE-dsRNAs. We speculate that NS1 restrains cytosolic accumulation of TE-dsRNAs in order to limit their impact on immunostimulatory, inflammatory and cell death antiviral pathways.

## Results and discussion

### Integrated analysis of subcellular host transcriptomes during IAV infection reveals the importance of NS1 in suppressing cytokine and chemokine induction

**Figure 1:**
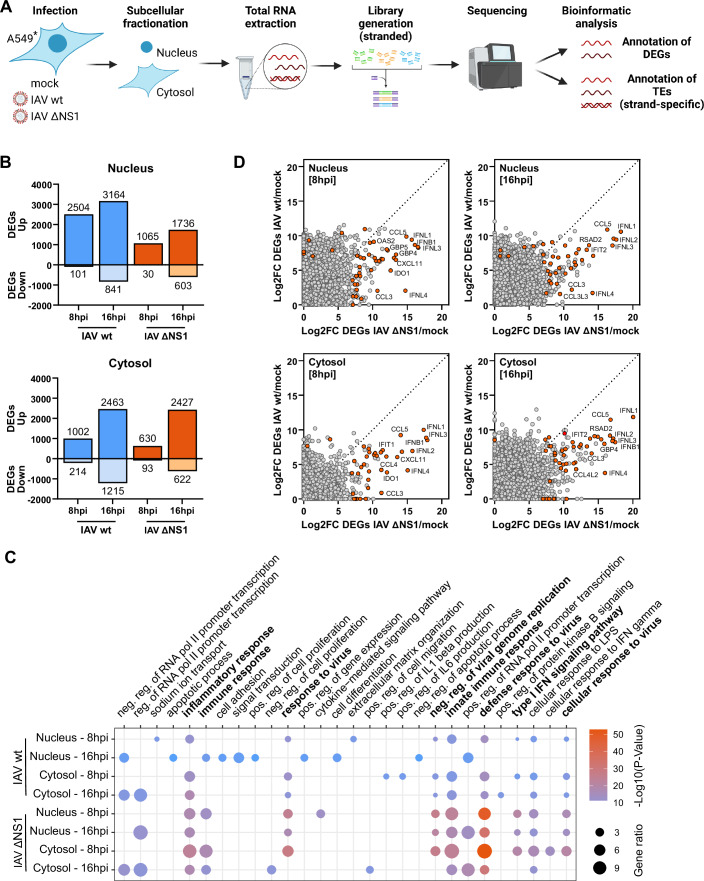
Integrated analysis of subcellular host transcriptomes during IAV infection reveals the importance of NS1 in suppressing cytokine and chemokine induction. (**A**) Schematic representation of the experimental set-up: illustration depicting the experimental design, whereby A549 cells (*A549-ACE2/TMPRSS2) were infected with wt IAV or IAV ΔNS1 [MOI 5 PFU/cell] for 8 h or 16 h, followed by subcellular fractionation, RNA extraction, and transcriptome analysis. For sequencing analysis, *n* = 3 biological replicates were performed. (**B**) Transcriptome analysis of subcellular fractions: comparison of the total number of differentially expressed genes (DEGs) in distinct subcellular fractions of A549-ACE2/TMPRSS2 cells following infection with wt IAV or IAV ΔNS1 [MOI 5 PFU/cell] at the times indicated. DEGs were defined by log_2_FC > 2 and *P*_adj_ <0.1 (significance determined using the Wald test from the DESeq2 package). Analysis is based on *n* = 3 biological replicates. (**C**) Gene ontology analysis: gene ontology (GO) analysis was performed on DEGs. Dot plot visualization displays the top ten enriched GO terms for each condition (*P* < 0.001, significance determined using a modified Fisher’s exact test in DAVID). Dot color indicates the *P* value, while dot size represents the percentage of genes enriched from the total gene set. Analysis is based on *n* = 3 biological replicates. (**D**) Comparative analysis of DEGs: comparison of DEGs between wt IAV and IAV ΔNS1-infected subcellular fractions at the indicated times. Cytokines, chemokines, and interferon-stimulated genes (ISGs) with a log_2_FC > 7 and *P*_adj_ <0.1 in either condition are highlighted in orange, emphasizing genes with substantial expression changes. Analysis is based on *n* = 3 biological replicates. See also Dataset EV1.

To gain information on expression of protein-coding genes and TEs during IAV infection, we established an in-depth transcriptomic analysis workflow (Fig. 1A). Given the importance of IAV NS1 as an IFN-antagonist (Ayllon and García-Sastre, 2015), we sought to compare the differential transcriptional responses induced by wt IAV or IAV ΔNS1 (both based on A/WSN/1933, WSN; H1N1). We therefore performed host RNA-Seq of an A549-derived human lung carcinoma epithelial cell line at 8 or 16 h post infection with either wt IAV or IAV ΔNS1 (MOI of 5 PFU/cell), and compared this with mock-infected cells. Importantly, following initial cell harvest, we separated cells into nucleus-associated and cytosolic fractions, and extracted total RNA from these fractions to allow an understanding of the subcellular localization of the examined transcripts. To enhance resolution and simplify sample complexity, ribosomal RNA depletion was performed prior to sequencing. We opted for ribosomal depletion over the polyadenylation (polyA) enrichment method to ensure comprehensive transcriptomic coverage, mitigating the risk of overlooking TE transcripts lacking a polyA tail. Furthermore, the experiment and subsequent bioinformatics analysis was specifically designed to evaluate the presence of derepressed TEs in a double-stranded state: RNA sequencing was conducted in a strand-specific manner, thus facilitating subsequent assignment of reads to their original strands, and downstream bioinformatics analysis focused on determining relative changes in TE transcripts with overlapping sense and antisense transcripts.

In all replicates of our sequencing experiment, verification of subcellular fraction purity was achieved using RT-qPCR for established markers: the long non-coding RNA MALAT1 for nuclear localization (Hutchinson et al, 2007), and GAPDH mRNA for its increased prevalence in the cytosol (Samacoits et al, 2018) (Fig. EV1A). In addition, western blot analysis for Histone H3 and GAPDH served as a nuclear and cytosolic marker, respectively (Fig. EV1B). Transcriptomic profiling of the fractionated samples revealed substantial alterations in cellular gene expression following both IAV infections compared to mock-infected cells across all timepoints and fractions (Fig. 1B; Dataset EV1). Infection with wt IAV for 8 or 16 h resulted in 2504 and 3164 genes, respectively, exhibiting significant induction (log_2_ fold change >2; adjusted *P* value < 0.1) in the nucleus-associated fraction. In addition, 1002 and 2463 genes, respectively, were upregulated in the cytosolic fraction upon infection with wt IAV (Fig. 1B). Infection with IAV ΔNS1 resulted in comparable numbers of differentially expressed genes (DEGs) in the cytosolic fraction as compared to wt IAV infection, with 630 and 2427 genes significantly upregulated after 8 or 16 h, respectively (Fig. 1B). This was also broadly the case in the nucleus-associated fraction, with IAV ΔNS1 infection leading to about half the number of DEGs as compared to wt IAV infection. Gene enrichment analyses of DEGs revealed that while both wt IAV and IAV ΔNS1 modulated similar pathways such as “response to virus”, “innate immune response”, and “inflammatory response”, IAV ΔNS1 exhibited much stronger effects (Fig. 1C). This observation was further substantiated through direct comparison of specific gene expression changes between wt IAV and IAV ΔNS1-infected cells: both virus infections elicited upregulation of RNAs encoding cytokines, chemokines, and ISGs across both subcellular fractions, but the magnitude of antiviral response gene induction was notably greater in IAV ΔNS1-infected cells (Figs. 1D and EV1C; Dataset EV1). To rule out that these effects were caused by potential differences in viral replication kinetics, we used RT-qPCR to confirm similar levels of viral M segment RNA between wt IAV and IAV ΔNS1 infections (Fig. EV1D). Taken together, these transcriptomic analyses reveal distinct patterns of host DEGs during infection of A549-derived cells with wt IAV and IAV ΔNS1, and highlight the role of NS1 in limiting induction of key immune and inflammatory pathways.

**Figure EV1:**

Fractionation quality controls and subcellular host transcriptome analysis during IAV infection. (**A**) Subcellular fraction purity assessment: RT-qPCR analysis was conducted on RNA samples from experiments similar to those shown in Fig. 1 with wt IAV. GAPDH was utilized as a cytosolic fraction marker, and MALAT1 served as a nucleus-associated fraction marker. Bars represent mean values and SDs from *n* = 3 biological replicates (each dot corresponds to one replicate). Significance was determined by unpaired *t* test on log-transformed data (**P = 0.0024; ns, non-significant). (**B**) Subcellular fraction purity assessment: western blot analysis was conducted on cell lysates from experiments similar to those shown in Fig. 1 with wt IAV, probing for the specific marker proteins GAPDH (cytosolic fraction) and Histone H3 (nucleus-associated fraction). Data are representative of *n* = 3 biological replicates. (**C**) Differential gene expression analysis in subcellular fractions: volcano plots depicting the differential gene expression in A549-ACE2/TMPRSS2 cells following infection with wt IAV or IAV ΔNS1 [MOI = 5 PFU/cell] at the indicated times and in the indicated subcellular fractions. Genes with statistically significant differential expression (*P*_adj_ <0.1, determined using the Wald test from the DESeq2 package) and a log_2_FC > 2 were considered differentially expressed genes (DEGs). Cytokines, chemokines, and interferon-stimulated genes (ISGs) with a log_2_FC > 7 and *P*_adj_ <0.1 are highlighted in orange. Selected gene names are shown. Analysis is based on *n* = 3 biological replicates. See also Dataset EV1. (**D**) Assessment of infection comparability between wt IAV and IAV ΔNS1: A549 cells were infected with wt IAV or IAV ΔNS1 [MOI = 5 PFU/cell] for 16 h. RT-qPCR analysis for IAV M segment was performed on RNA samples. Bars represent mean values and SDs from *n* = 3 biological replicates (each dot corresponds to one replicate). Significance was determined by ordinary one-way ANOVA on log-transformed data (****P ≤ 0.0001; ns non-significant).

### Bioinformatics analysis of IAV-induced TE-dsRNAs identifies increased cytosolic accumulation in the absence of NS1

**Figure 2:**
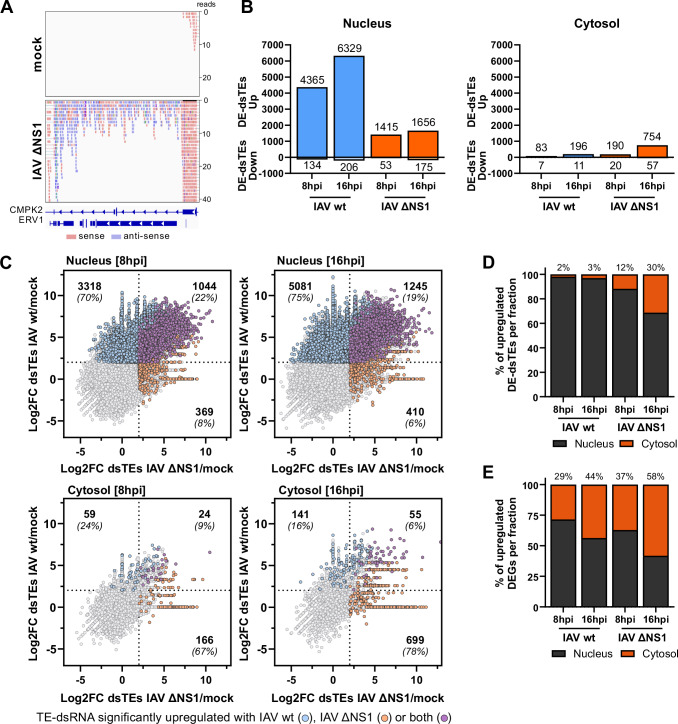
Bioinformatics analysis of IAV-induced TE-dsRNAs identifies increased cytosolic accumulation in the absence of NS1. (**A**) Bioinformatic strategy for read filtering: in our bioinformatic approach, overlapping reads in both sense and antisense directions were filtered for and assessed as likely to form double-stranded RNA (dsRNA). For the example TE (ERV1) and flanking regions, sense reads are shown in red, while antisense reads are depicted in blue. (**B**) Analysis of differentially expressed TEs in subcellular fractions: quantification of differentially expressed TEs with evidence for double-strandedness (DE-dsTEs) (log_2_FC > 2; *P*_adj_ <0.1, significance determined using the Wald test from the DESeq2 package) in nucleus-associated and cytosolic fractions following infection with wt IAV or IAV ΔNS1 [MOI 5 PFU/cell] for the indicated times. Analysis is based on *n* = 3 biological replicates. (**C**) Comparative analysis of DE-dsTEs in response to wt IAV or IAV ΔNS1 infection: visualization of significantly upregulated dsTEs in response to wt IAV (blue) and IAV ΔNS1 (orange) infections. dsTEs significantly upregulated in both conditions are highlighted in purple. The numbers in each quadrant represent both the absolute counts and the relative percentages of DE-dsTEs that were significantly upregulated. Analysis is based on *n* = 3 biological replicates. (**D**) Percentage of DE-dsTEs in distinct subcellular fractions: bar chart of the percentage of DE-dsTEs within each respective subcellular fraction during wt IAV and IAV ΔNS1 [MOI 5 PFU/cell] infections at the indicated timepoint. The percentages annotated above the bars specifically denote the cytosolic fractions. Analysis is based on *n* = 3 biological replicates. (**E**) Percentage of DEGs in distinct subcellular fractions: bar chart of the percentage of DEGs within the respective subcellular fractions at the indicated times post infection. The percentages annotated above the bars specifically denote the cytosolic fractions. Analysis is based on *n* = 3 biological replicates. See also Dataset EV2.

We next sought to characterize TE upregulation during IAV infection and assess evidence for the production of double-stranded TE RNAs (TE-dsRNAs). For this purpose, we developed a bioinformatics pipeline for the in silico detection of dsRNA from stranded, paired, short-read RNA-Seq data. The pipeline initially trimmed and aligned reads to the human genome, allowing gapped alignments. It then counted reads that both mapped uniquely to TEs and had the potential to form dsRNA with reads from the opposing strand, yielding estimates of dsRNA read counts for each TE. Finally, the differential expression of TE-dsRNA transcripts was calculated. One caveat to our “unique mappers” pipeline is that younger TEs and highly repetitive older TEs may be excluded from the analysis, biasing the types of elements we could study (Bourque et al, 2018). Furthermore, the analysis is based on “bulk” RNA-Seq data, introducing the possibility that not all complementary reads originated from the same cell. Notwithstanding these unavoidable experimental caveats, an illustrative depiction of the annotation of sense and antisense reads is presented in Fig. 2A. The bioinformatics analysis unveiled a substantial upregulation of TEs with reads in both sense and antisense orientations in the nucleus-associated fraction in response to infection with both wt IAV and IAV ΔNS1, which is suggestive of sequences that could form potential TE-dsRNAs (Figs. 2B,C and EV2A; and Dataset EV2). Specifically, cells infected with wt IAV for 8 or 16 h exhibited significant upregulation of 4365 and 6329 TE-dsRNAs (log_2_ fold change >2; adjusted *P* value < 0.1), respectively, in the nucleus-associated fraction compared to mock-infected cells (Fig. 2B). Remarkably, only a low number of these TE-dsRNAs were detected in the cytosolic fraction during wt IAV infection (83 and 196 at 8 h and 16 h, respectively) (Fig. 2B). Conversely, infection with IAV ΔNS1 led to fewer differentially expressed TE-dsRNAs in the nucleus-associated fraction (only 1415 and 1656 TE-dsRNAs upregulated after 8 h and 16 h of infection, respectively) (Fig. 2B), which may be due to the lower replicative capacity of IAV ΔNS1 as compared to wt IAV. However, it was striking that cells infected with IAV ΔNS1 exhibited a substantially greater number of potential TE-dsRNAs in the cytosol (190 and 754 at 8 h and 16 h, respectively) (Fig. 2B). Collectively, the proportion of cytosolic versus nucleus-associated TE-dsRNAs was markedly higher in IAV ΔNS1-infected cells compared to wt IAV-infected cells (Fig. 2C,D). Specifically, at 16 h post IAV ΔNS1 infection, about 30% of the total TE-dsRNAs were localized in the cytosol, whereas in wt IAV-infected cells, only 3% of the induced TE-dsRNAs demonstrated cytosolic localization (Fig. 2D). The identities of cytosol-localized TE-dsRNAs in IAV ΔNS1-infected cells, in particular those not found in the cytosol of wt IAV-infected cells, are listed in Dataset EV2. Notably, this enhanced cytosolic localization of TE-dsRNAs in IAV ΔNS1-infected cells appeared to be specific to this class of RNAs, as no clear differences in the nucleus-associated and cytosolic distribution of non-TE DEGs were observed between wt IAV and IAV ΔNS1 (Fig. 2E). Furthermore, multiple different TE families and classes were observed as dsRNAs in the cytosol, including LTRs, LINEs, SINEs, and DNA transposons, and we did not observe any family being notably overrepresented or any potential length bias, e.g., toward the shorter Alu/SINE elements (Fig. EV2B,C), although the caveat of our “unique mappers” strategy is that some TEs may have been excluded. Importantly, application of our bioinformatics pipeline to re-analyze similarly-generated raw RNA-Seq data from independent infection of normal human bronchial epithelial (BEAS-2B) cells with the recent Perth/09 H3N2 IAV strain also uncovered clear evidence for differential expression of TEs with evidence of double-strandedness, although comprehensive analysis was compromised by the shorter read length of these available data (Fig. EV2D) (Fabozzi et al, 2018). These sequencing data provide evidence that IAV infection not only leads to substantial induction of TEs (Schmidt et al, 2019), but that many of these TEs have the potential to form TE-dsRNAs via sequence complementarity. Furthermore, these fractionation data reveal that such TE-dsRNAs can accumulate in the cytosol of IAV-infected cells, particularly in the absence of the NS1 protein.

**Figure EV2:**

Differential expression and classification of TE-dsRNAs in subcellular fractions during IAV infection. (**A**) Differential expression of TE-dsRNAs in subcellular fractions: volcano plots illustrating the differential expression of TE-dsRNAs in the specified subcellular fractions of A549-ACE2/TMPRSS2 cells following infection with wt IAV or IAV ΔNS1 [MOI = 5 PFU/cell]. TEs reaching the thresholds for statistical significance (*P*_adj_ <0.1, determined using the Wald test from the DESeq2 package) and log_2_FC > 2, with bioinformatic evidence for double-strandedness, were considered differentially expressed TE-dsRNAs. Selected element names are shown. Analysis is based on *n* = 3 biological replicates. See also Dataset EV2. (**B**, **C**) Subclasses of significantly upregulated TE-dsRNAs in nuclear and cytosolic fractions: absolute (**B**) and relative (**C**) numbers of TE subclasses significantly upregulated during wt IAV or IAV ΔNS1 infections at the indicated times and compartments are shown for LTR (long terminal repeat retrotransposon), LINE (long interspersed nuclear element), SINE (short interspersed nuclear element) and DNA transposon elements. Analysis is based on *n* = 3 biological replicates. (**D**) Reanalysis of publicly available RNA-Seq data for TE-dsRNAs during IAV infection: volcano plots illustrating the differential expression of TE-dsRNAs in normal human bronchial epithelial cells (BEAS-2B) following infection for 24 h with IAV strain A/Perth/16/2009 (H3N2) [MOI = 1 PFU/cell]. TEs reaching the thresholds for statistical significance (*P*_adj_ <0.1, determined using the Wald test from the DESeq2 package) and log_2_FC > 1.5, with bioinformatic evidence for double-strandedness, were considered differentially expressed TE-dsRNAs. Selected element names are shown.

### dsRNA-immunoprecipitation assays validate the double-stranded nature of some IAV-induced TE RNAs and their increased cytosolic accumulation in the absence of NS1

**Figure 3:**
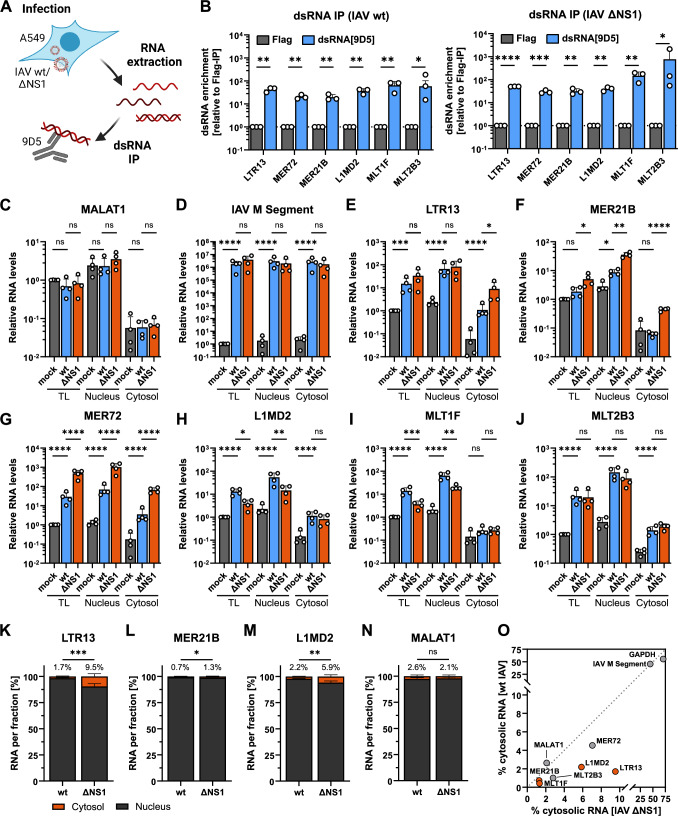
dsRNA-immunoprecipitation assays validate the double-stranded nature of some IAV-induced TE RNAs and their increased cytosolic accumulation in the absence of NS1. (**A**) Experimental set-up for dsRNA immunoprecipitations: schematic representation of the assay aimed at discerning the double-stranded nature of upregulated TEs by immunoprecipitation (IP) with a specific dsRNA antibody (9D5) from total extracted RNA of infected cells. (**B**) dsRNA immunoprecipitation: A549 cells were infected with wt IAV or IAV ΔNS1 [MOI 5 PFU/cell] for 16 h before total RNA was extracted and IP was performed using the 9D5 or anti-Flag antibodies. Selected TEs were subsequently analyzed via RT-qPCR. Enrichment is presented as fold change over the negative control IP (anti-Flag antibody). Bars represent mean values and SDs from *n* = 3 biological replicates (each dot corresponds to one replicate). Significance was determined by a one-sample *t* test on log-transformed data, comparing the sample means to their individual controls (*P ≤ 0.05; **P ≤ 0.01; ***P ≤ 0.001; ****P ≤ 0.0001). Exact *P* values are provided in the Source Data file. (**C**–**J**) Validation of TE expression and localization by RT-qPCR: RT-qPCR analysis of selected RNAs in subcellular fractions following mock, wt IAV, or IAV ΔNS1 infections [MOI 5 PFU/cell; 16 h] in A549 cells. MALAT1 served as a nuclear-localized fractionation control, IAV M segment served as an infection-level control. Bars represent mean values and SDs from *n* = 4 biological replicates (each dot corresponds to one replicate). Significance was determined by ordinary one-way ANOVA with Šídák’s multiple comparisons test on log-transformed data (*P ≤ 0.05; **P ≤ 0.01; ****P ≤ 0.0001; ns non-significant). Exact *P* values are provided in the Source Data file. (**K**–**N**) Expression ratios of selected TEs: quantification of the selected RNAs determined via RT-qPCR, depicted as the percentage of transcript per fraction. Bars represent mean values and SDs from *n* = 4 biological replicates. Significance was determined by unpaired *t* test (*P ≤ 0.05; **P ≤ 0.01; ***P ≤ 0.001; ns non-significant). Exact *P* values are provided in the Source Data file. (**O**) Cytosolic presence of TEs between wt IAV and IAV ΔNS1-infected cells: comparison of the cytosolic presence of selected TEs and other genes between wt IAV and IAV ΔNS1-infected cells. The percentage of each RNA present in the cytosolic fraction (versus the nucleus-associated fraction) for each infected condition is plotted. Each dot represents the mean of the *n* = 4 biological replicates shown in (**C**–**J**). Transcripts with a statistically significant cytosolic localization upon IAV ΔNS1 infection as compared to wt IAV infection are highlighted in orange (data from (**K**–**N**) and Fig. EV5A). GAPDH and IAV M segment RNAs were used as references. Source data are available online for this figure.

**Figure EV3:**
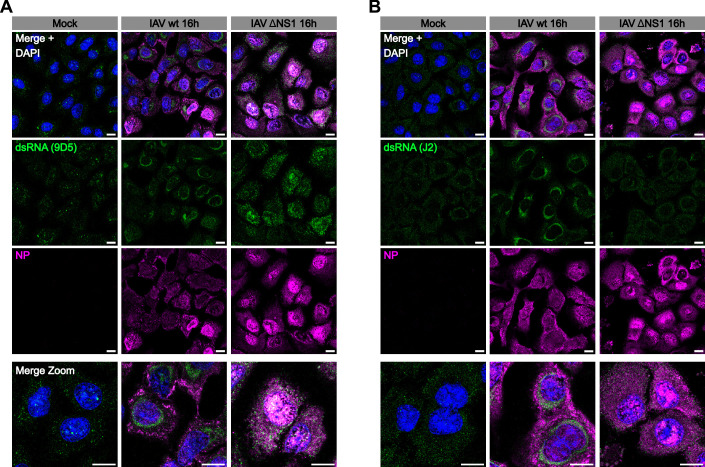
Immunofluorescence confocal microscopy of total dsRNA in A549 cells infected with wt IAV or IAV ΔNS1. (**A**, **B**) A549 cells were infected, or mock, with wt IAV or IAV ΔNS1 [MOI = 1 PFU/cell] for 16 h. Subsequently, cells were fixed and permeabilized with methanol, treated with Proteinase K, and stained with specific antibodies against dsRNA (9D5 in (**A**); J2 in (**B**)), the IAV NP protein, and with DAPI to stain nuclei. Immunofluorescence confocal microscopy images are shown, representative of at least *n* = 2 biological replicates. Scale bars represent 10 µm.

To provide orthogonal evidence that IAV infection leads to upregulation of TE-dsRNA species, we established a protocol to immunoprecipitate dsRNA from cells and quantify specific TE RNA transcripts by RT-qPCR (Fig. 3A). Specifically, A549 cells were infected with either wt IAV or IAV ΔNS1 for 16 h, after which total RNA was harvested using Trizol extraction, and dsRNA species were immunoprecipitated using the well-characterized anti-dsRNA antibody, 9D5 (Son et al, 2015). We chose to use 9D5 as, in our hands, this antibody exhibited a greater sensitivity towards detecting dsRNA in IAV ΔNS1-infected A549 cells than another commonly used anti-dsRNA antibody, J2 (Fig. EV3A,B). Following immunoprecipitation, RNA was again extracted using Trizol and was analyzed by RT-qPCR for a selection of six TEs for which we had transcriptomic and bioinformatic evidence for double-strandedness (LTR13, MER72, MER21B, L1MD2, MLT1F, MLT2B3). All TEs examined were clearly enriched in immunoprecipitates from the 9D5 antibody as compared to the isotype control antibody (anti-Flag) (Fig. 3B). Furthermore, TE-dsRNA was detected in response to infection with both viruses, wt IAV as well as IAV ΔNS1. These data support the findings from our RNA-Seq and bioinformatics analyses, and further indicate that some IAV-induced TEs can form dsRNA species.

**Figure EV5:**
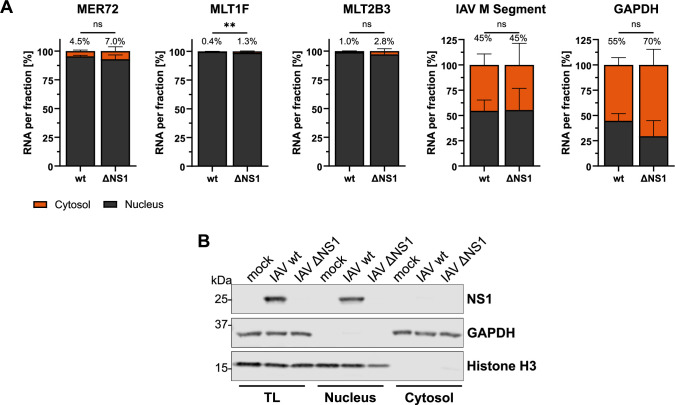
Relative nuclear/cytosolic distribution of specific RNAs and proteins in A549 cells infected with wt IAV or IAV ΔNS1. (**A**) RNA expression ratios: RT-qPCR data for selected RNAs from Fig. 3C–J are depicted as the percentage of transcript per fraction. Bars represent mean values and SDs from *n* = 4 biological replicates. The percentages annotated above the bars specifically denote the cytosolic fractions. Significance was determined by unpaired *t* test (**P ≤ 0.0035; ns non-significant). (**B**) Western blot analysis of IAV NS1 expression in subcellular fractions of A549 cells infected, or mock, with wt IAV or IAV ΔNS1 [MOI = 5 PFU/cell] for 16 h. The purity of subcellular fractions was also assessed by probing for the specific marker proteins GAPDH (cytosolic fraction) and Histone H3 (nucleus-associated fraction). Data are representative of *n* = 3 biological replicates.

**Figure EV4:**
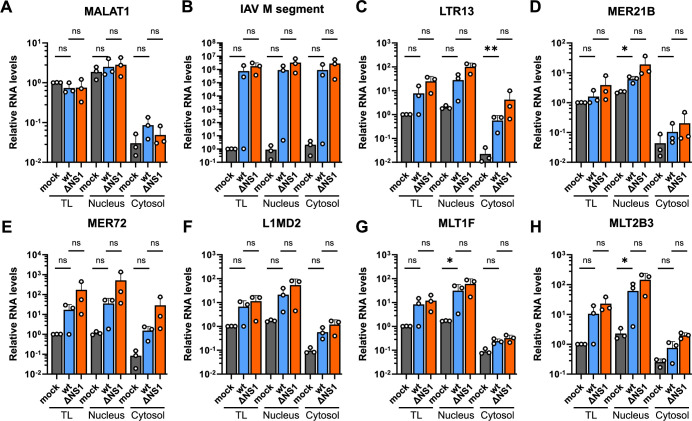
Nuclear/cytosolic distribution of specific RNAs in MRC5 cells infected with wt IAV or IAV ΔNS1. (**A**–**H**) Assessment of TE expression and localization by RT-qPCR: RT-qPCR analysis of selected RNAs in subcellular fractions following mock, wt IAV, or IAV ΔNS1 infections [MOI 5 PFU/cell; 16 h] in MRC5 cells. MALAT1 served as a nuclear-localized fractionation control, IAV M segment served as an infection-level control. Bars represent mean values and SDs from *n* = 3 biological replicates (each dot corresponds to one replicate). Significance was determined by ordinary one-way ANOVA with Šídák’s multiple comparisons test on log-transformed data (*P ≤ 0.05; **P ≤ 0.01; ns, non-significant).

We next specifically assessed the IAV-induced subcellular distribution of these six TEs for which we had both RNA-Seq and dsRNA-immunoprecipitation data supporting their double-strandedness. In particular, we compared the impact of wt IAV and IAV ΔNS1 infection on TE-dsRNA localization. A549 cells were infected, or mock, with wt IAV or IAV ΔNS1 at an MOI of 5 PFU/cell for 16 h, and RT-qPCR for specific candidate TE-dsRNAs was subsequently performed on RNA extracted from total cell lysates or from nucleus-associated and cytosolic fractions. RT-qPCR for the nuclear marker MALAT1 confirmed successful fractionation, and that there were no gross differences in nuclear/cytosolic localization of this RNA species between wt IAV and IAV ΔNS1-infected cells (Fig. 3C). In addition, RT-qPCR for the IAV M segment confirmed similar infection levels between wt and ΔNS1 viruses (Fig. 3D). Furthermore, all six TEs assayed were clearly induced by both wt IAV and IAV ΔNS1, and were found in both the nucleus-associated and cytosolic fractions (Fig. 3E–J). However, when comparing infections with wt IAV and IAV ΔNS1, it was clear that IAV ΔNS1 infection resulted in higher amounts of cytosolic RNA for many candidate TE-dsRNAs (e.g., LTR13, MER21B, and MER72) (Fig. 3E–J). Generally similar results and trends were obtained from wt IAV and IAV ΔNS1 infections of human MRC5 cells, which were used as a primary-like untransformed diploid cell model (Fig. EV4A–H). In A549s, effects were most evident when the data were expressed as the ratio between cytosolic and nuclear transcript abundance (Figs. 3K–O and EV5A). Such increased cytosolic accumulation upon IAV ΔNS1 infection as compared to wt IAV infection was specific for the tested candidate TE-dsRNAs, as the ratio between cytosolic and nuclear localization was comparable between the two infections for other RNA species, such as MALAT1, GAPDH and the viral M segment (Figs. 3N,O and EV5A). The data are also consistent with immunofluorescence-based microscopy results that revealed a diffuse abundance of general dsRNA species in the cytosol of IAV ΔNS1-infected cells, as compared with wt IAV-infected cells where dsRNA species appeared to accumulate in the nucleus or in nucleus-associated perinuclear regions (Fig. EV3). These findings further indicate that IAV-induced TE-dsRNAs can accumulate in the cytosol of infected cells, particularly in the absence of NS1.

### IAV NS1 protein can interact with infection-induced TE-dsRNAs

**Figure 4:**
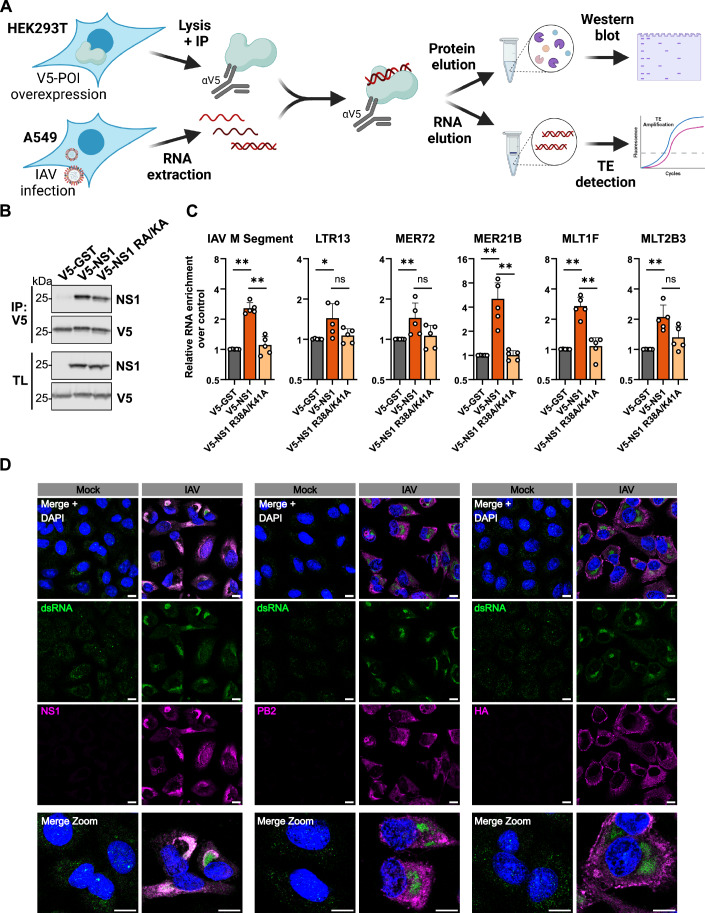
IAV NS1 protein can interact with infection-induced TE-dsRNAs. (**A**) Experimental set-up for NS1 immunoprecipitation (IP): schematic representation of the assay aimed at delineating if NS1 interacts with de-repressed TEs. V5-tagged proteins of interest (POI) were immunoprecipitated from transfected HEK293T cells, and the washed immunoprecipitates were incubated with total RNA extracted from IAV-infected A549 cells [MOI 5 PFU/cell; 16 h]. Selected TEs were quantified by RT-qPCR following further immunoprecipitation. (**B**) Western blot analysis of immunoprecipitated proteins: western blot analysis illustrating the successful immunoprecipitation of V5-tagged GST, wt NS1, or a dsRNA-binding deficient mutant NS1 (R38A/K41A; RA/KA) from transfected HEK293T cells. Total lysate (TL) served as a transfection control. Data are representative of *n* = 5 biological replicates. (**C**) RT-qPCR analysis of TEs co-precipitating with NS1: RT-qPCR analysis of selected TEs and viral M co-precipitated with wt NS1 or the dsRNA-binding deficient mutant NS1 (R38A/K41A; RA/KA). Enrichment is presented as fold change over the negative control (V5-GST). Bars represent mean values and SDs from *n* = 5 biological replicates (each dot corresponds to one replicate). Significance was determined by Mann–Whitney *U* test (*P ≤ 0.05; **P ≤ 0.01; ns non-significant). Exact *P* values are provided in the Source Data file. (**D**) Specific co-localization of IAV NS1 with dsRNA in perinuclear regions: A549 cells were infected, or mock, with wt IAV [MOI = 1 PFU/cell] for 24 h. Cells were subsequently fixed and permeabilized with methanol, treated with Proteinase K, and then stained with antibodies specific for dsRNA (9D5) and the IAV NS1, PB2, or HA proteins. Nuclei were stained with DAPI. Immunofluorescence confocal microscopy images are shown, representative of at least *n* = 2 biological replicates. Scale bars represent 10 µm. Source data are available online for this figure.

Next, we addressed the possible mechanism by which NS1 expression may affect the localization of TE-dsRNAs. Given that we observed enhanced cytosolic abundance of TE-dsRNAs during infection with IAV ΔNS1, that NS1 is known to bind non-specifically to dsRNA (Hatada and Fukuda, 1992), and that a substantial fraction of NS1 is associated with the nucleus during infection (Fig. EV5B) (Ayllon et al, 2012; Kerry et al, 2011), we hypothesized that NS1 might be able to interact with IAV-induced TE-dsRNAs as a means to limit their accumulation in the cytosol. To this end, we immunoprecipitated transiently expressed V5-tagged wt NS1, or a well-characterized mutant NS1 (R38A/K41A) that lacks dsRNA-binding activity (Wang et al, 1999), from HEK293T cells and incubated the bead-captured NS1s (or V5-tagged control, GST) with total RNA extracted from IAV-infected A549 cells (Fig. 4A). Following further immunoprecipitation, protein extracts were assessed by western blot to validate equal immunoprecipitation of V5-tagged GST, wt NS1 and NS1-R38A/K41A (Fig. 4B). RT-qPCR analysis of co-precipitated RNA revealed that (as expected) V5-tagged wt NS1, but not V5-tagged NS1-R38A/K41A could co-precipitate a fraction of the IAV M segment RNA (Hatada et al, 1992). The RNA fold-enrichment for wt NS1 over the GST control was only approximately twofold, which may be an experimental sensitivity caveat relating to wt NS1 already being associated non-specifically with HEK293T-derived RNAs prior to incubation with total RNA extracted from IAV-infected A549 cells. Nevertheless, several candidate TE-dsRNAs, including LTR13, MER72, MER21B, MLT1F and MLT2B3 were also specifically enriched with V5-tagged wt NS1, but not with V5-tagged NS1-R38A/K41A (Fig. 4C). Consistent with these findings, immunofluorescence-based confocal microscopy revealed a close and specific association of general dsRNA species with NS1, but not other viral proteins, in nucleus-associated perinuclear regions within wt IAV-infected cells (Fig. 4D) (Ayllon et al, 2012). Overall, these data expand the range of dsRNA molecules that NS1 can form a complex with and suggest a plausible mechanism by which NS1 expression can limit cytosolic accumulation of host TE-dsRNAs: by interacting with, and sequestering, them.

### Concluding remarks

In this study, we used RNA-Seq, computational analysis, and experimental approaches to characterize the expression and localization of uniquely mapping human TEs in response to IAV infection. Our results demonstrate that IAV induces substantial genome-wide derepression of TEs, and that many TE RNAs might have a double-stranded nature. Furthermore, while these TE-dsRNAs are predominantly associated with the nucleus during infection with wt IAV, infection with an IAV lacking expression of the dsRNA-binding protein, NS1, results in their increased cytosolic accumulation. A plausible mechanism by which NS1 expression prevents the accumulation of TE-dsRNAs in the cytosol was suggested by the interaction of NS1 with TE-dsRNAs. We speculate that IAV-induced host TE-dsRNAs have the potential to be recognized by a range of cellular antiviral dsRNA-binding proteins, should these components localize to the same cytosolic compartment. Thus, NS1-mediated sequestration of TE-dsRNA could play an important role in mitigating activation of several immune and inflammatory pathways.

Previous studies have characterized TE expression in response to infection with different viruses and found that infection-induced genome-wide TE expression often occurs in the vicinity of antiviral response genes (Chen et al, 2023; Macchietto et al, 2020). Thus, derepression of TEs might have cis-acting consequences on regulating the expression of these genes (Chuong et al, 2017). However, our study adds an additional layer to virus-induced TE-mediated regulation of antiviral immunity: we analyzed the localization of TE transcripts and their potential to form dsRNA structures, hallmarks for successful detection by cytosolic host innate immune sensors. Our data could indicate that the widespread virus-induced upregulation of TE-dsRNAs might be an additional line of defense activated by the cell in response to virus infection, with these host-derived TE-dsRNAs potentially acting in a similar way to viral “pathogen-associated molecular patterns” (PAMPs) to stimulate antiviral defenses. It is presently unclear why such an additional set of “host PAMPs” may be generated and what role they would play over and above viral PAMPs. However, one could speculate that given many viruses have evolved measures to hide their own genetic material from host detection (e.g., by replicating in specialized bodies or protecting their vRNA 5′ ends) (Markiewicz et al, 2021), as well as antagonistic mechanisms to target and inhibit specific sensing pathways (e.g., IAV NS1 targeting RIG-I) (Ayllon and García-Sastre, 2015), TE-dsRNA induction and broad activation of multiple sensors might be an effective cellular “mechanism of last resort” to amplify innate and inflammatory immune responses. Future studies are clearly necessary to dissect the contributions of viral versus host PAMPs with respect to the immune activities of individual sensor proteins at the endogenous level and in different cell types or infection scenarios. In this context, it will be key to assess these processes in diverse non-transformed cell models where TE sequences and regulatory mechanisms may differ.

Given that infection-triggered TE-dependent induction of an antiviral response (by viral mimicry) poses a powerful threat to the invading virus, it is conceivable that viruses may have developed antagonistic measures to counteract this. Indeed, we could show that IAV NS1 can interact with TE-dsRNAs, potentially sequestering them in the nucleus or nucleus-associated perinuclear regions and preventing their detection by cytosolic sensors. Although NS1 is widely recognized as an antagonist of the host immune response on many levels, our recent findings unveil an additional dimension to its multifaceted repertoire and provide insights into a previously unappreciated possible host-derived dsRNA target for this viral protein. Nevertheless, a clear limitation of our study is that we focused mainly on infections with WSN/33-based IAVs in the A549 cell-line model, and future studies will have to assess the generality of our findings using both disease-relevant viral strains and different primary cell systems.

In conclusion, our study sheds light on the interactions between IAV and TEs within infected host cells. We demonstrate that IAV NS1 can interact with de-repressed TEs induced during infection, likely sequestering most of them in or around the nucleus and thereby influencing their subcellular distribution and immunostimulatory potential. These findings underscore the multifaceted interplay between viral proteins, host cellular processes, and the regulation of TE expression during IAV infection, providing potential insights into the mechanisms underlying viral pathogenesis and host-activated immune or inflammatory responses.

## Methods

**Table:** Reagents and tools table

Reagent/resource	Reference or source	Identifier or catalog number
**Experimental models**		
Human A549	ATCC	#CCL-185
Human HEK293T	ATCC	#CRL-3216
Human MRC5	ATCC	#CCL-171
Canine MDCK	ATCC	#CRL-2936
A549-ACE2/TMPRSS2	Kind gift from Sam Wilson, Cambridge, UK (Rihn et al, 2021)	
Canine MDCK NS1/NPro	This study	
IAV H1N1 strain A/WSN/1933 (WSN/33)	Lab stock	
IAV H1N1 strain A/WSN/1933 lacking NS1 expression (WSN/33 ΔNS1)	Kind gift from Balaji Manicassamy, Iowa, USA (Manicassamy et al, 2010)	
**Recombinant DNA**		
pCAGGS-GST-V5	Described previously (Hale et al, 2008; Lopes et al, 2017)	
pLVX-PR8-NS1-wt-V5	Described previously (Hale et al, 2008; Lopes et al, 2017)	
pLVX-PR8-NS1-R38A/K41A-V5	Described previously (Hale et al, 2008; Lopes et al, 2017)	
**Antibodies**		
Mouse monoclonal anti-dsRNA [9D5] IgG1	Absolute Antibody	#Ab00458-1.1
Mouse monoclonal anti-Flag M2 IgG1	Sigma-Aldrich	#F1804
Mouse monoclonal anti-V5 antibody	Bio-Rad	#MCA1360
Mouse monoclonal anti-GAPDH (0411)	Santa Cruz	#sc-47724
Rabbit polyclonal anti-Histone H3	Abcam	#ab1791
Rabbit polyclonal anti-NS1	GeneTex	#GTX125990
Rabbit polyclonal anti-NP	Kind gift from Jovan Pavlovic, Zurich, Switzerland	
IRDye 680CW goat anti-mouse IgG	Li-Cor	#926–68070
IRDye 800CW goat anti-rabbit IgG	Li-Cor	#926–32211
Mouse monoclonal anti-dsRNA [J2]	Engscicons	#J2-0804
Rabbit polyclonal anti-NS1	Invitrogen	#PA5-32243
Rabbit polyclonal anti-HA	Sino Biological	#11692-T54
Rabbit polyclonal anti-PB2	Kind gift from Peter Palese, New York, USA	
Donkey anti-mouse IgG Alexa Fluor 488	Thermo Fisher Scientific	#A21202
Donkey anti-rabbit IgG Alexa Fluor 555	Thermo Fisher Scientific	#A-21428
**Oligonucleotides and other sequence-based reagents**		
GAPDH-F primer	Microsynth AG	CTGGCGTCTTCACCACCATGG
GAPDH-R primer	Microsynth AG	CATCACGCCACAGTTTCCCGG
MALAT1-F primer	Microsynth AG	GAAGGAAGGAGCGCTAACGA
MALAT1-R primer	Microsynth AG	TACCAACCACTCGCTTTCCC
IAV M (WHO M30F2) primer	Microsynth AG	ATGAGYCTTYTAACCGAGGTCGAAACG
IAV M (WHO M264R3) primer	Microsynth AG	TGGACAAANCGTCTACGCTGCAG
18s-rRNA-F primer	Microsynth AG	GGCCCTGTAATTGGAATGACTC
18s-rRNA-R primer	Microsynth AG	CCAAGATCCAACTACGAGCTT
LTR13-F primer	Microsynth AG	AAGCTGGCCCACAGTTATCC
LTR13-R primer	Microsynth AG	AGGTGCACAGAGTGGAACAG
MER72-F primer	Microsynth AG	TGATCAGCCTTCCCTCCTGA
MER72-R primer	Microsynth AG	AGGACATAGAGGCACCCTGT
L1MD2-F primer	Microsynth AG	CTTCCCAGTTGTACAGGCGT
L1MD2-R primer	Microsynth AG	GCGTGATGGATGGTCGATCT
MLT1F-F primer	Microsynth AG	GGCATATGGCTTCCAAGGCT
MLT1F-R primer	Microsynth AG	ACATGGCCCTCTCCATAGGT
MLT2B3-F primer	Microsynth AG	GTTCTTGGCCTTTGGCCTTG
MLT2B3-R primer	Microsynth AG	CTGTAAGCTGGAGACCGTGG
**Chemicals, enzymes, and other reagents**		
Dulbecco’s modified Eagle’s medium (DMEM)	Gibco Life Technologies	#11965092
Minimum Essential Medium Eagle (MEM)	Sigma-Aldrich	#M2279
Fetal Bovine Serum (FBS)	Gibco Life Technologies	#A5256701
Penicillin/streptomycin (P/S)	Gibco Life Technologies	#11528876
GlutaMAX	Thermo Fisher Scientific	#35050038
Non-Essential Amino Acids	Thermo Fisher Scientific	#11140035
cOmplete™ Protease Inhibitor Cocktail	Roche	#11836170
RNasin® Ribonuclease Inhibitor	Promega	#N2615
Trizol	Thermo Fisher Scientific	#15596026
GlycoBlue™ Coprecipitant	Thermo Fisher Scientific	#AM9515
Protein G Sepharose™ beads	Millipore	#P3296
FuGENE® HD Transfection Reagent	Promega	#E2311
Amersham™ Protran® western blotting membranes, nitrocellulose	Merck	#GE10600002
DAPI	Sigma-Aldrich	#10236276001
ProLong Gold Antifade Mountant	Thermo Fisher Scientific	#P36930
**Software**		
fastp-0.23.2	Chen et al, 2018	
FastQC-0.12.1	Andrews, 2010	
STAR-2.7.10b	Dobin et al, 2013	
Rust	Matsakis and Klock, 2014	
featureCounts (subread-2.0.1 package)	Liao et al, 2014	
R-4.3.1	R Core Team, 2023	
DESeq2	Love et al, 2014	
dplyr	Wickham H, 2023	
Rsamtools	Morgan, 2023	
LasX	Leica	
ImageJ/Fiji	https://imagej.net	
GraphPad Prism 7	GraphPad	
DAVID	Huang da et al, 2009	
Repbase	Bao et al, 2015	
**Other**		
SuperScript™ IV First-Strand Synthesis System	Thermo Fisher Scientific	#18091300
PowerTrack™ SYBR Green Master Mix for qPCR	Thermo Fisher Scientific	#A46109
7300 Real-Time PCR System	Applied Biosystems	
Fragment Analyzer	Agilent	
TruSeq Stranded Total RNA Library Prep Gold	Illumina	#RS-122-2301
Novaseq 6000	Illumina	
Odyssey XF NIR imaging system	Li-Cor	
SP8 confocal microscope	Leica	

### Cells and viruses

**Figure EV6:**
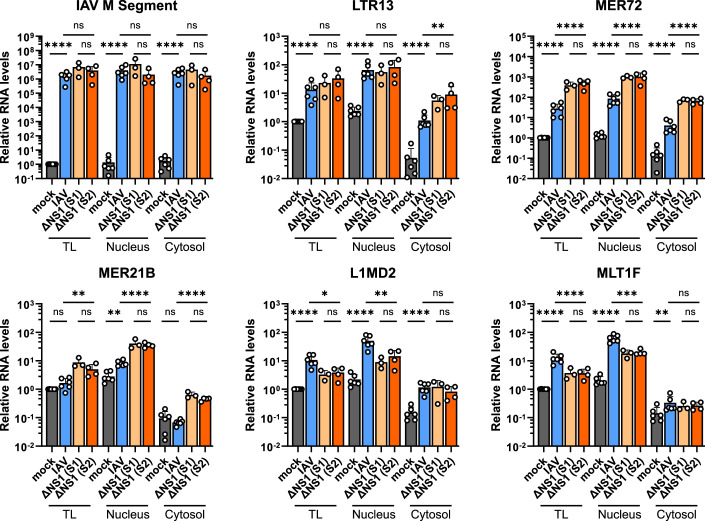
Validation of differential TE expression during infection of A549 cells with different IAV ΔNS1 stocks. RT-qPCR analysis to validate the differential expression of selected TEs and viral M RNA in subcellular fractions following mock, wt IAV, IAV ΔNS1 S1, or IAV ΔNS1 S2 infection [MOI = 5 PFU/cell] of A549 cells for 16 h. Following the initial RNA-Seq experiments, we discovered that the IAV ΔNS1 stock used (S1) was inadvertently contaminated with parainfluenza virus 5 (PIV5). A new non-contaminated IAV ΔNS1 stock was therefore prepared (S2) and both stocks were tested for their ability to induce TEs side-by-side. Some data for mock, wt IAV, and IAV ΔNS1 (S2) are also shown in Fig. 3. Bars represent mean values and SDs from at least *n* = 3 biological replicates, each with at least *n* = 2 technical replicates (each dot corresponds to one experiment). Significance was determined by ordinary one-way ANOVA with Šídák’s multiple comparisons test on log-transformed data (*P ≤ 0.05; **P ≤ 0.01; ***P ≤ 0.001; ****P ≤ 0.0001; ns non-significant).

Human A549 cells (#CCL-185, ATCC), human HEK293T cells (#CRL-3216, ATCC), and canine MDCK cells (#CRL-2936, ATCC) were cultured in Dulbecco’s modified Eagle’s medium (DMEM) (Life Technologies) supplemented with 10% (vol/vol) FBS, 100 units/ml penicillin, and 100 μg/ml streptomycin (Gibco Life Technologies). Human MRC5 cells (#CCL-171, ATCC) were cultured in Minimum Essential Medium Eagle (MEM) (Sigma-Aldrich) supplemented with 10% (vol/vol) FBS, 100 units/ml penicillin, 100 μg/ml streptomycin (Gibco Life Technologies), 2 mM GlutaMAX (Thermo Fisher Scientific), and 1% non-essential amino acids (NEAA; Thermo Fisher Scientific). A549-ACE2/TMPRSS2 cells were a kind gift from Sam Wilson (Cambridge, UK) (Rihn et al, 2021). A549-ACE2/TMPRSS2 cells were used for the initial RNA-Seq analysis, while A549 cells were used for all follow-up experiments. Cell lines were not authenticated, but were routinely tested for mycoplasma contamination (GATC, Germany). None of the cell lines used ever tested positive for Mycoplasma. IAV strain A/WSN/1933 (WSN/33; H1N1) was propagated in MDCK cells. A WSN/33 virus engineered to lack NS1 expression (IAV ΔNS1) was kindly provided by Balaji Manicassamy (Iowa, USA) (Manicassamy et al, 2010) and was propagated in MDCK cells stably expressing the IAV NS1 protein (PR8 strain) (Hale et al, 2006) and the NPro protein from Bovine Viral Diarrhea Virus (Hilton et al, 2006). Following the initial RNA-Seq experiments, we discovered that the IAV ΔNS1 stock used was inadvertently contaminated with parainfluenza virus 5 (PIV5). A new non-contaminated IAV ΔNS1 stock was prepared, and small-scale experiments comparing TE expression in response to both the contaminated (S1) and non-contaminated (S2) IAV ΔNS1 stocks revealed identical TE responses (Fig. EV6). We therefore conclude that the contamination did not grossly impact the large-scale RNA-Seq results as presented. All subsequent validation and follow-up experiments were performed with PCR-confirmed non-contaminated IAV ΔNS1 stocks.

### Virus infections

Generally, cells were seeded at 6 × 10^5^ cells per well of a 6-well plate or 2.5 × 10^6^ cells per 10-cm culture dish and infected the next day at the indicated multiplicity of infection (MOI). Virus inoculum was prepared in PBS supplemented with 0.3% BSA, 1 mM Ca^2+^/Mg^2+^, 100 units/ml penicillin, and 100 μg/ml streptomycin. Cells were incubated with the inoculum for 1 h, washed with PBS, and then overlaid with DMEM (or MEM) supplemented with 0.1% FBS, 0.3% BSA, 20 mM HEPES, 100 units/ml penicillin, and 100 μg/ml streptomycin. For the RNA-Seq analysis, three independent replicates were performed.

### Biosafety

All work with the described infectious IAVs was performed in a biosafety level 2 laboratory at the Institute of Medical Virology, University of Zurich, Switzerland, following approval by the Swiss Federal Office of Public Health (approval number A151455/2).

### Plasmids

Mammalian expression vectors pCAGGS-GST-V5, pLVX-PR8-NS1-wt-V5, and pLVX-PR8-NS1-R38A/K41A-V5 were described previously (Hale et al, 2008; Lopes et al, 2017).

### Subcellular fractionation

Cytosolic and nucleus-associated fractions were isolated using an adapted protocol (Conrad and Ørom, 2017), and total RNA was subsequently extracted from these fractions. Briefly, cells were seeded in six-well plates and infected 24 h later for the indicated times. At the point of harvest, cells were detached by trypsinization, centrifuged at 1200 rpm, and the resulting cell pellets were washed two times with ice-cold PBS. Cells were lysed for 5 min on ice in 100 μl cell lysis buffer (10 mM Tris pH 7.4, 150 mM NaCl, 0.15% IGEPAL CA-630; sterile filtered through a Steritop-GP 0.22-μm filter unit and freshly supplemented with cOmplete™ Protease Inhibitor Cocktail (#11836170, Roche) and RNasin® Ribonuclease Inhibitor (#N2615, Promega; 1:1000)). Lysates were gently overlaid on top of 250 μl ice-cold sucrose buffer (10 mM Tris pH 7.4, 150 mM NaCl, 24% sucrose; sterile filtered through a Steritop-GP 0.22 μm filter unit and freshly supplemented with RNasin® (1:1000)) in protein LoBind 1.5-ml tubes and centrifuged at 3500 × *g* for 10 min. The resulting supernatant containing the cytosolic fraction was cleared by centrifugation at 14,000 × *g* for 1 min in a new 1.5-ml microcentrifuge tube, and the supernatant was collected. For RNA isolation from this fraction, 1 ml of Trizol (#15596026, Thermo Fisher Scientific) was added per 200 μl of cytosolic fraction. The pellet fraction containing nucleus-associated material was briefly rinsed with 1 ml ice-cold PBS-EDTA (1× PBS, 500 μM EDTA pH 8.0; freshly supplemented with RNasin® (1:1000)), followed by a 1 min centrifugation at 3500 × *g* before gently removing the PBS-EDTA from the nucleus-associated pellet. For RNA isolation from this fraction, pellets were directly lysed in 1 ml of Trizol reagent.

### RNA extraction

Material was directly lysed in 1 ml of Trizol (#15596026, Thermo Fisher Scientific) and incubated at room temperature (RT) for 10 min. In total, 200 μl of chloroform was added, samples were mixed by vigorous shaking, and centrifuged for 15 min at 13,000 rpm at 4 °C. The aqueous phase was transferred to a new RNase-free tube containing 1.3 μl GlycoBlue™ Coprecipitant (#AM9515, Thermo Fisher Scientific), 1 volume of isopropanol (600 μl) was added, and after vigorous shaking samples were centrifuged for 15 min at 13,000 rpm at 4 °C. The pellet containing RNA was washed twice with ice-cold 70% ethanol using centrifugation steps of 10 min at 13,000 rpm at 4 °C. RNA was subsequently dissolved in nuclease-free water. RNA was used immediately for reverse transcription and the remaining RNA was stored at −80 °C.

### RT-qPCR

Extracted RNA was reverse-transcribed using the SuperScript™ IV First-Strand Synthesis System (#18091300, Thermo Fisher Scientific) with oligo(dT) primers and random hexamers according to the manufacturer’s instructions. RT-qPCR was then performed using PowerTrack™ SYBR Green Master Mix for qPCR (#A46109, Thermo Fisher Scientific) using specific forward and reverse primers in a 7300 Real-Time PCR System (Applied Biosystems). The relative gene expression was calculated with the ΔΔCt method (Livak and Schmittgen, 2001), using 18s-rRNA for normalization.

### Library preparation and sequencing

Library preparation and sequencing were performed by the Functional Genomics Center Zurich (FGCZ). Briefly, the quality of isolated RNA was determined with a Fragment Analyzer (Agilent, Santa Clara, CA, USA). The TruSeq Stranded Total RNA Library Prep Gold (Illumina, Inc., CA, USA) was used in subsequent steps. Briefly, total RNA samples (100–1000 ng) were depleted of ribosomal RNA and then reverse-transcribed into double-stranded cDNA. The cDNA samples were fragmented, end-repaired and adenylated before ligation of TruSeq adapters containing unique dual indices (UDI) for multiplexing. Fragments containing TruSeq adapters on both ends were selectively enriched with PCR. The quality and quantity of the enriched libraries were validated using a Fragment Analyzer (Agilent, Santa Clara, CA, USA). The product was a smear with an average fragment size of ~260 bp. The libraries were normalized to 10 nM in 10 mM Tris-Cl, pH 8.5, with 0.1% Tween 20. A Novaseq 6000 (Illumina, Inc., CA, USA) was used for cluster generation and sequencing according to a standard protocol. Sequencing was paired-end at 2 × 150 bp.

### Sequencing data analysis

Reads were trimmed and base corrected using fastp-0.23.2 (Chen et al, 2018), and reads shorter than 35 nucleotides were excluded. The quality of the trimmed reads was checked using FastQC-0.12.1 (Andrews, 2010). Trimmed reads were then aligned to the hg38 human genome using STAR-2.7.10b (Dobin et al, 2013), keeping only reads with a single best alignment and a maximum number of three mismatches. A custom Rust (Matsakis and Klock, 2014) program was written to count the number of potentially double-stranded reads. When provided with TE coordinates and the aligned reads, the program counts how many reads from each strand map to each TE locus and overlap with reads from the opposite strand. This results in two counts of reads per locus: the number of reads from the forward strand that overlap with reads from the reverse strand, and vice versa. The minimum of these counts was taken as an estimate of the number of double-stranded reads, assuming that the remaining reads are single-stranded. The read counts for non-TE genes were obtained using featureCounts from the subread-2.0.1 package (Liao et al, 2014). An R-4.3.1 script (R Core Team, 2023) was written to calculate the differential expression. Differential expression analysis of TE loci was performed using DESeq2 (Love et al, 2014) using size factors calculated from non-TE read counts. Data transformations and visualizations were performed using dplyr (Wickham H, 2023) and Rsamtools (Morgan, 2023). A similar pipeline was employed to re-analyze a publicly available RNA-Seq dataset (GSE61517) from normal human bronchial epithelial cells (BEAS-2B) infected for 24 h with IAV strain A/Perth/16/2009 (H3N2) [MOI = 1 PFU/cell] (Fabozzi et al, 2018), albeit the shorter read length and multimapping compromised a comprehensive analysis.

### dsRNA immunoprecipitation

A549 cells seeded in 10-cm culture dishes were infected with wt IAV or IAV ΔNS1 [MOI 5 PFU/cell; 16 h], and total RNA was extracted by Trizol-chloroform extraction as described above. For each condition, 2 μl anti-dsRNA [9D5] (IgG1; #Ab00458-1.1, Absolute Antibody) or mouse monoclonal anti-Flag M2 (IgG1; #F1804, Sigma-Aldrich), were coupled to 30 μl Protein G Sepharose™ beads (#P3296, Millipore) by 2 h incubation at 4 °C with rotation. Washed beads were incubated with 30 μg RNA in 300 μl PBS + 0.1% Triton X-100 (freshly supplemented with 1 μl RNasin®) for 2 h at 4 °C with rotation, followed by 4 washes with PBS + 0.1% Triton X-100 (freshly supplemented with RNasin® (1:1000)). RNA was subsequently eluted and extracted using Trizol.

### Co-immunoprecipitation of RNA with proteins

HEK293T cells (~6 × 10^5^ cells per six-well) were transfected with 1–3 μg of the indicated constructs using FuGENE® HD Transfection Reagent (#E2311, Promega) according to the manufacturer’s instructions. After approximately 24 h, cells were washed once with PBS and lysed for 15 min on ice in lysis buffer (50 mM Tris-HCl, pH 7.4, 300 mM NaCl, 1 mM EDTA, 1% Triton X-100) supplemented with cOmplete™ Protease Inhibitor Cocktail (#11836170, Roche). Lysates were then cleared by centrifugation for 15 min at 14,000 rpm at 4 °C and subjected to immunoprecipitation with anti-V5 mouse monoclonal antibody (#MCA1360, Bio-Rad) overnight at 4 °C with rotation in DNA/RNA LoBind tubes. Subsequently, 30 μl of Protein G Sepharose™ beads (#P3296, Millipore) were added and incubated for 2 h at 4 °C with rotation. The beads were washed twice with lysis buffer. The bead-captured immunoprecipitates were then incubated with 30 μg total extracted RNA from IAV [MOI 5 PFU/cell; 16 h] infected A549 cells in 300 μl PBS + 0.1% Triton X-100 (freshly supplemented with 1 μl RNasin®) for 2 h at 4 °C with rotation. Following this, beads were washed four times with PBS + 0.1% Triton X-100 (freshly supplemented with RNasin® (1:1000)). Bound RNAs were eluted and extracted using Trizol as described above.

### Western blot analysis

Lysates were prepared in 2× urea disruption buffer (6 M urea, 2 M β-mercaptoethanol, 4% SDS, bromophenol blue) followed by sonication to shear nucleic acids. Proteins were separated by SDS-PAGE, transferred to nitrocellulose membranes (Amersham), and detected using the following primary and secondary antibodies: mouse monoclonal anti-GAPDH (0411)(#sc-47724, Santa Cruz), rabbit polyclonal anti-Histone H3 (#ab1791, Abcam), rabbit polyclonal anti-NS1 (#GTX125990, GeneTex), mouse monoclonal anti-V5 (#MCA1360, Bio-Rad), IRDye 680CW goat anti-mouse IgG (#926–68070, Li-Cor), and IRDye 800CW goat anti-rabbit IgG (#926–32211, Li-Cor).

### Immunofluorescence and confocal microscopy

In total, 1 × 10^5^ A549 cells were seeded on glass coverslips in 24-well plates and infected with the indicated virus as described above. At the time of harvest, cells were washed twice with PBS and then fixed and permeabilized with 1 ml of ice-cold methanol (MeOH) for 15 min at −20 °C. Subsequently, cells were washed four times with 1 ml of ice-cold PBS and samples were incubated with 0.01 U/ml Proteinase K in 50 mM Tris-HCl (pH 8.0) and 5 mM CaCl_2_ for 30 min at 37 °C. After washing three times with PBS, blocking was performed with 2% fetal bovine serum (FBS) in PBS (500 µl/well) for 30–60 min at RT. Primary antibody staining was performed in PBS with 2% FBS using the following antibodies at the indicated dilutions: mouse monoclonal anti-dsRNA [9D5] (#Ab00458-1.1, Absolute Antibody; 1:1000), mouse monoclonal anti-dsRNA [J2] (#J2-0804, Engscicons; 1:200), rabbit polyclonal anti-NS1 (#PA5-32243, Invitrogen; 1:100), rabbit polyclonal anti-NP (kind gift from Jovan Pavlovic, Zurich, Switzerland; 1:1000), rabbit polyclonal anti-HA (#11692-T54, Sino Biological; 1:500), or rabbit polyclonal anti-PB2 (kind gift from Peter Palese, NY, USA; 1:1000). Cells were incubated with 200 µl of the primary antibody solution overnight at 4 °C with gentle shaking. Cells were subsequently washed three times with PBS and incubated for 1 h at RT in the dark with the appropriate secondary antibody diluted in PBS/2% FBS and DAPI (#10236276001, Sigma-Aldrich; 1:1000). Secondary antibodies used were: donkey anti-mouse IgG Alexa Fluor 488 (#A21202, Thermo Fisher Scientific; 1:1000) and donkey anti-rabbit IgG Alexa Fluor 555 (#A-21428, Thermo Fisher Scientific; 1:1000). Cells were then washed three times with PBS, followed by two final washes with distilled water (ddH_2_O). Coverslips were mounted onto glass slides using 8 µl of ProLong Gold Antifade Mountant (#P36930, Thermo Fisher Scientific), and slides were allowed to dry in the dark at RT for at least 24 h. Samples were imaged with an SP8 confocal microscope (Leica) using LasX (Leica) software. Images were further processed using ImageJ/Fiji programs.

### Statistical analysis

Unless otherwise stated, statistical analyses were generally performed using GraphPad Prism 7 software. For RT-qPCR data, ΔCt values were analyzed. The statistical tests used and *P* values for significance are stated in the figure legends. Gene ontology enrichment analyses were performed on DEGs (log_2_FC > 2 and *P*_adj_ <0.1) using DAVID (Huang da et al, 2009). Dot plot visualization of the Top 10 enriched GO terms for each condition (*P* value < 0.001) was generated in R version 4.3.2 (R Core Team, 2023). TE classes were manually annotated according to Repbase (Bao et al, 2015).

### Graphics

Objects in the synopsis, as well as in Figs. 1A, 3A, and 4A were created in BioRender. Hale B (2025): https://BioRender.com/nxhqajh; https://BioRender.com/fytxn8t; https://BioRender.com/0vy5qwb; https://BioRender.com/z4ab6rp.

## Supplementary information

Peer Review File

Dataset EV1

Dataset EV2

Source data Fig. 3

Source data Fig. 4

Expanded View Figures

## Data Availability

The unique datasets produced in this study have been deposited to the European Nucleotide Archive (ENA) with the dataset identifier PRJEB75711. Code is available upon reasonable request. The source data of this paper are collected in the following database record: biostudies:S-SCDT-10_1038-S44319-025-00498-2.
