# Supplementary figures and images for: Regulated localization of transposable element RNA during influenza A virus infection
Source: EMBO Rep. 2025 Jun 16;26(14):3506–28. doi: 10.1038/s44319-025-00498-2 (PMC12287459; doi:10.1038/s44319-025-00498-2)

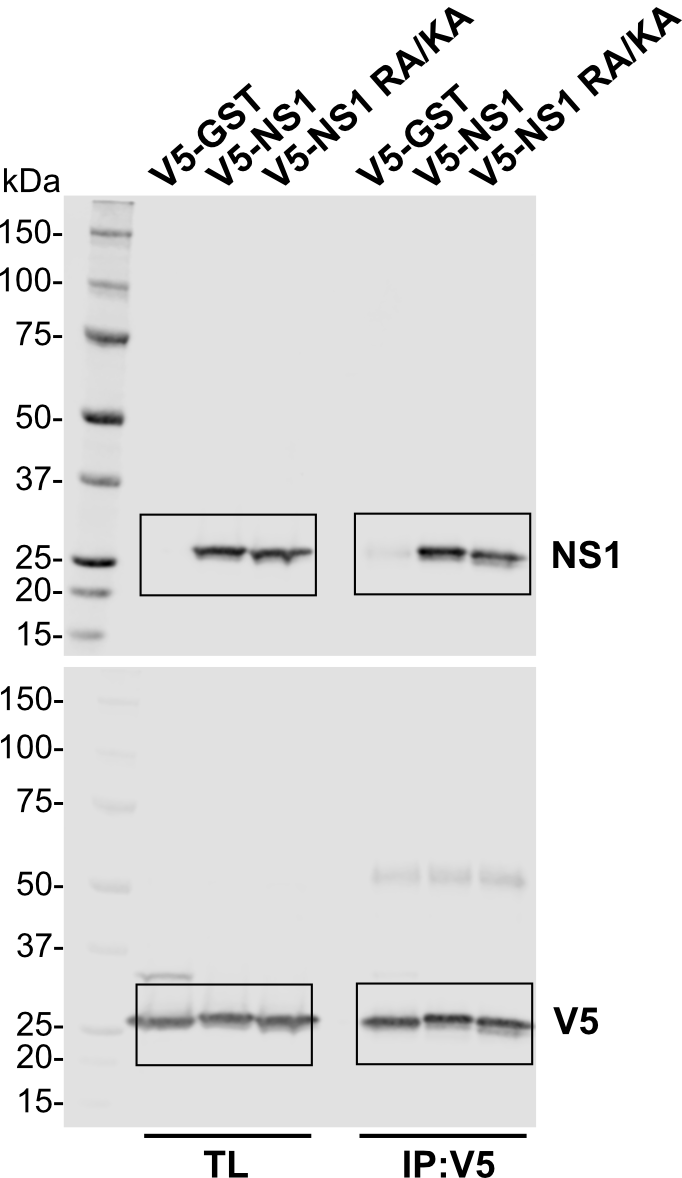

Supplement: Supplementary file 5 — Source data Fig. 4 [file 44319_2025_498_MOESM5_ESM.zip › Figure 4/Figure 4B/Figure 4B.tiff]

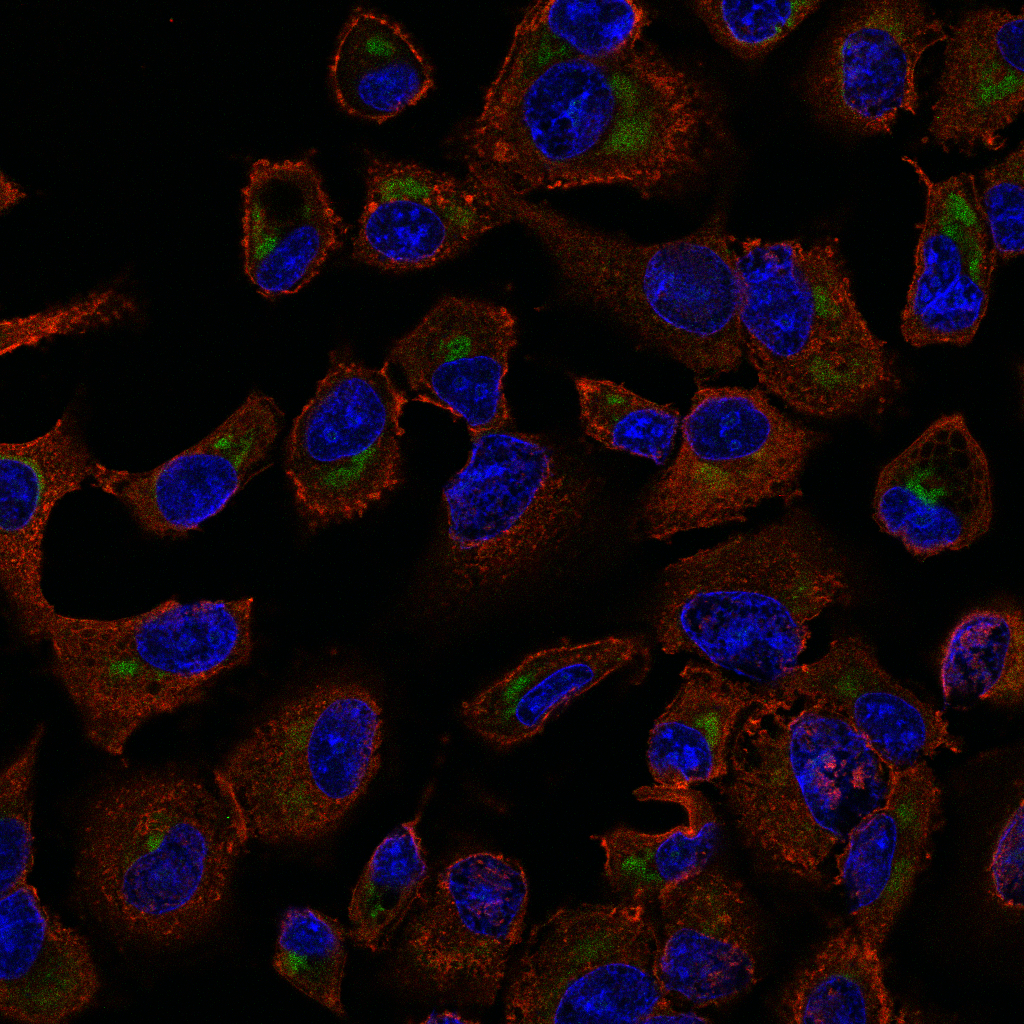

Supplement: Supplementary file 5 — Source data Fig. 4 [file 44319_2025_498_MOESM5_ESM.zip › Figure 4/Figure 4D/Fig.4D_HA/IAV_004.tif]

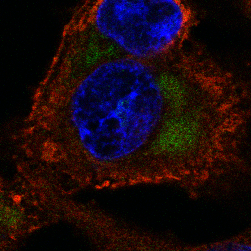

Supplement: Supplementary file 5 — Source data Fig. 4 [file 44319_2025_498_MOESM5_ESM.zip › Figure 4/Figure 4D/Fig.4D_HA/IAV_004_Crop001.tif]

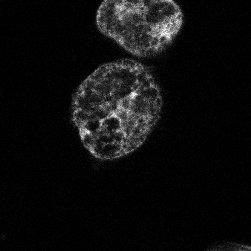

Supplement: Supplementary file 5 — Source data Fig. 4 [file 44319_2025_498_MOESM5_ESM.zip › Figure 4/Figure 4D/Fig.4D_HA/IAV_004_Crop001_RAW_ch00.tif]

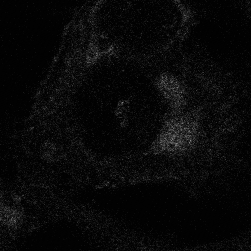

Supplement: Supplementary file 5 — Source data Fig. 4 [file 44319_2025_498_MOESM5_ESM.zip › Figure 4/Figure 4D/Fig.4D_HA/IAV_004_Crop001_RAW_ch01.tif]

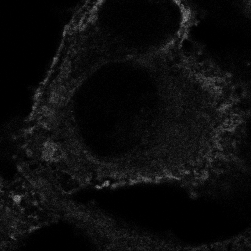

Supplement: Supplementary file 5 — Source data Fig. 4 [file 44319_2025_498_MOESM5_ESM.zip › Figure 4/Figure 4D/Fig.4D_HA/IAV_004_Crop001_RAW_ch02.tif]

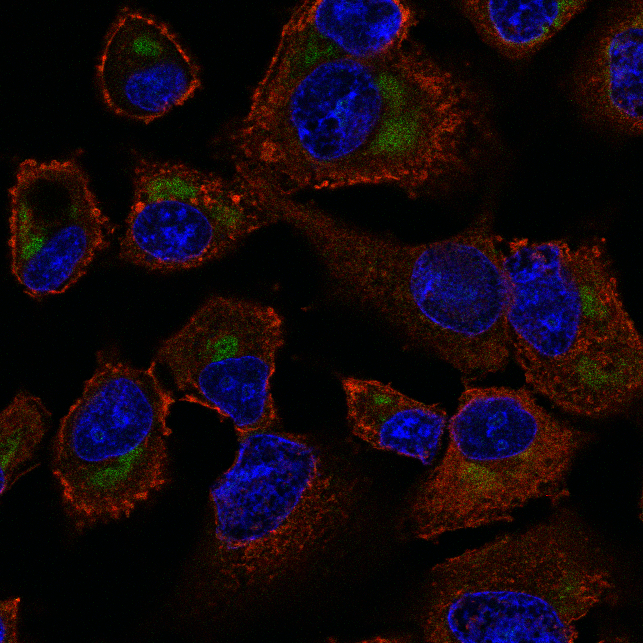

Supplement: Supplementary file 5 — Source data Fig. 4 [file 44319_2025_498_MOESM5_ESM.zip › Figure 4/Figure 4D/Fig.4D_HA/IAV_004_Crop002.tif]

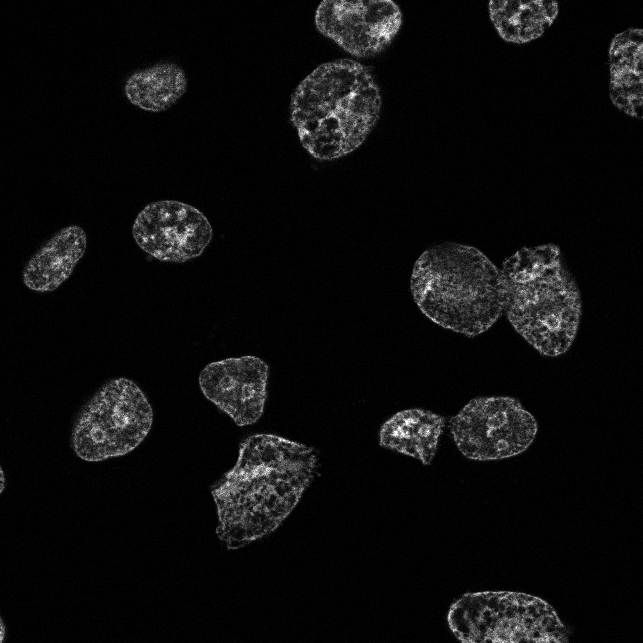

Supplement: Supplementary file 5 — Source data Fig. 4 [file 44319_2025_498_MOESM5_ESM.zip › Figure 4/Figure 4D/Fig.4D_HA/IAV_004_Crop002_RAW_ch00.tif]

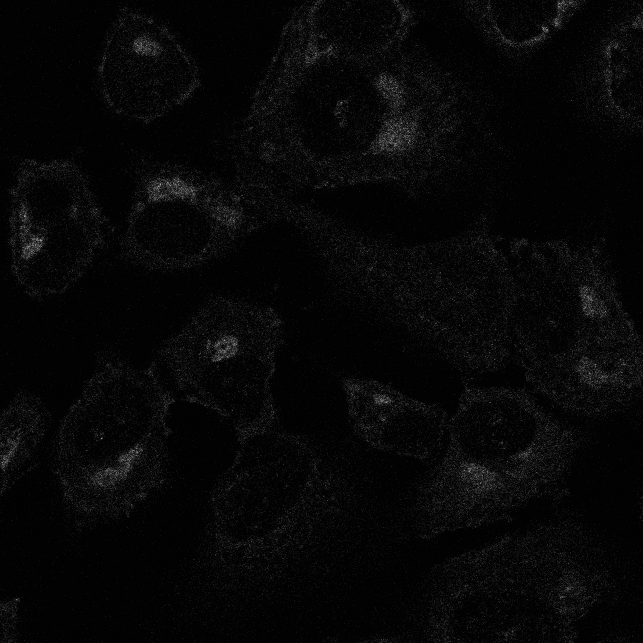

Supplement: Supplementary file 5 — Source data Fig. 4 [file 44319_2025_498_MOESM5_ESM.zip › Figure 4/Figure 4D/Fig.4D_HA/IAV_004_Crop002_RAW_ch01.tif]

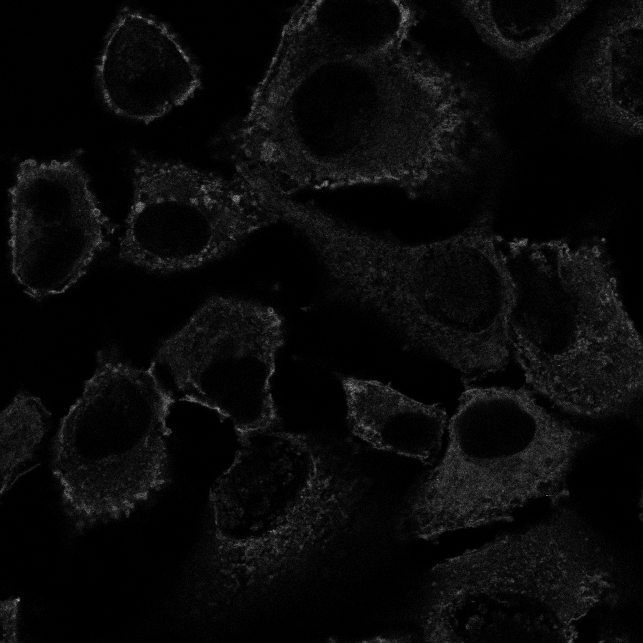

Supplement: Supplementary file 5 — Source data Fig. 4 [file 44319_2025_498_MOESM5_ESM.zip › Figure 4/Figure 4D/Fig.4D_HA/IAV_004_Crop002_RAW_ch02.tif]

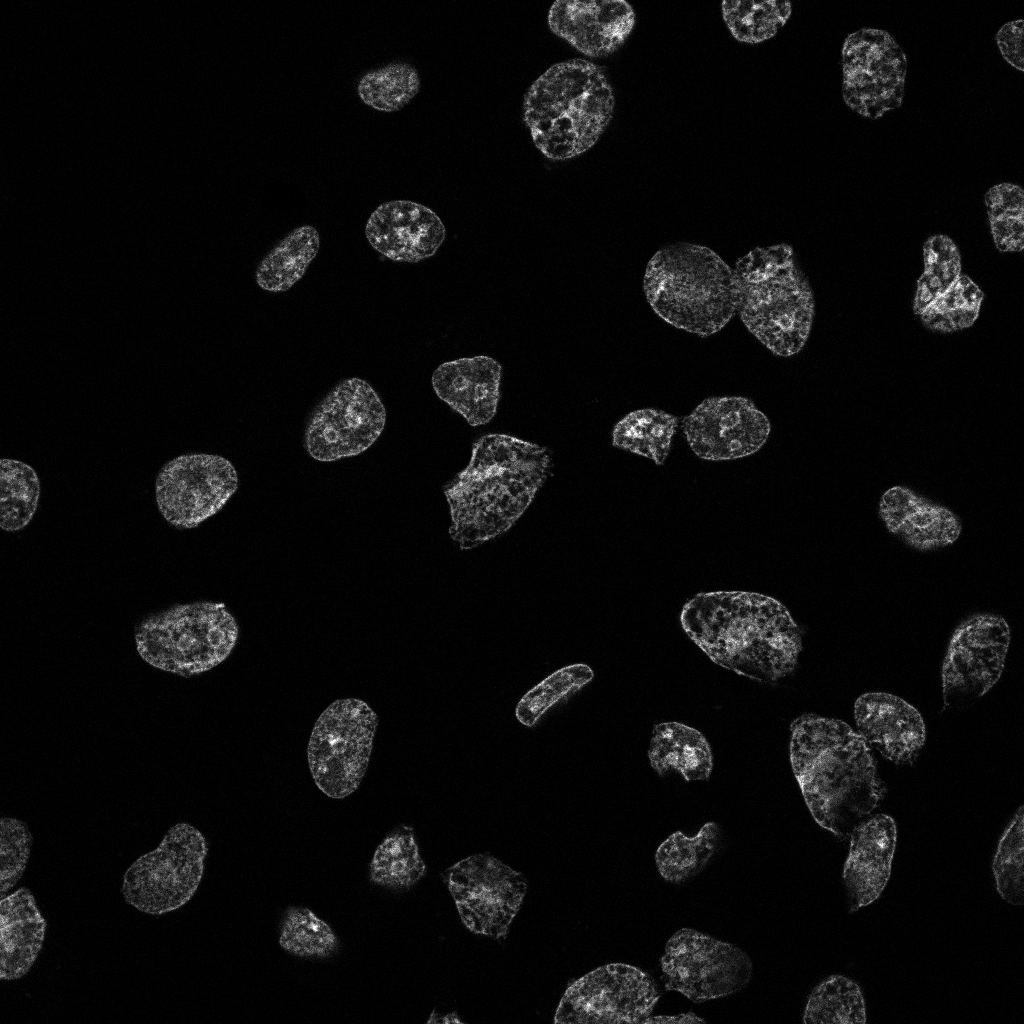

Supplement: Supplementary file 5 — Source data Fig. 4 [file 44319_2025_498_MOESM5_ESM.zip › Figure 4/Figure 4D/Fig.4D_HA/IAV_004_RAW_ch00.tif]

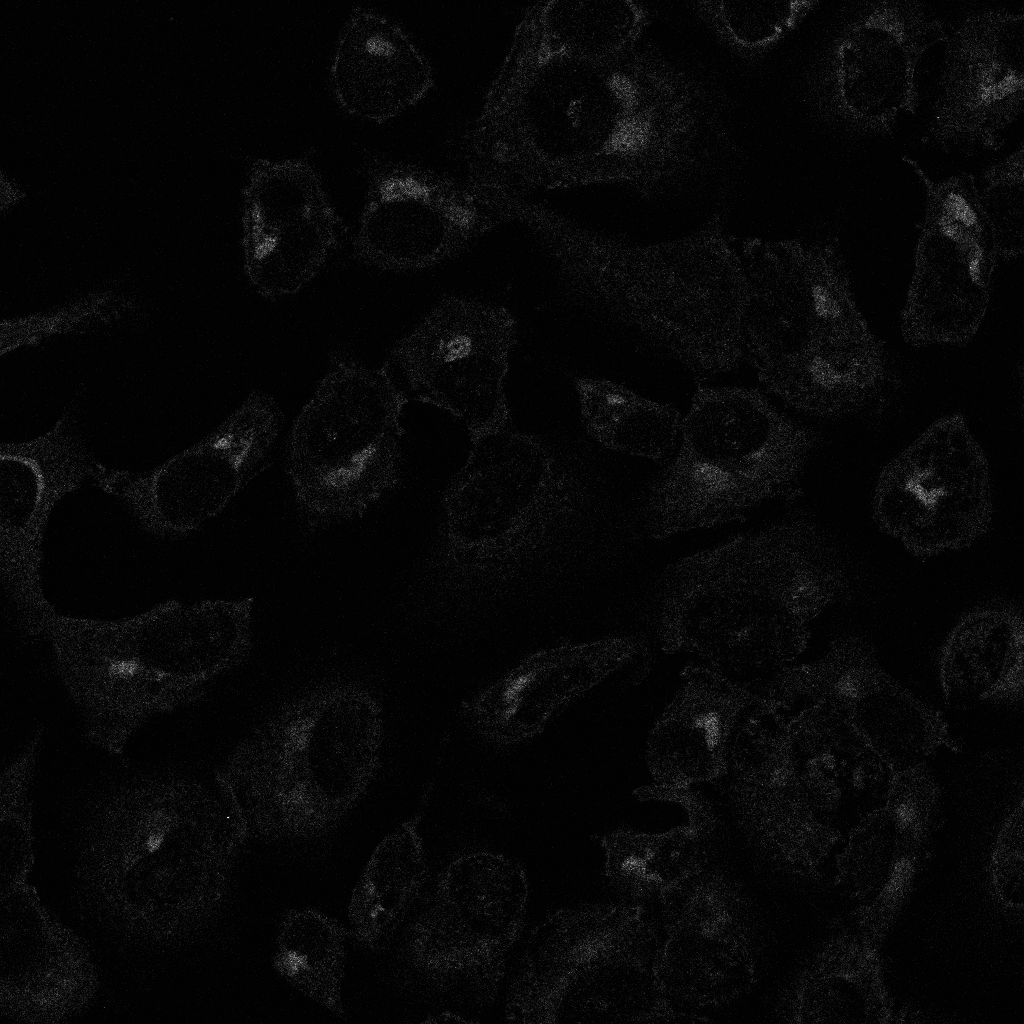

Supplement: Supplementary file 5 — Source data Fig. 4 [file 44319_2025_498_MOESM5_ESM.zip › Figure 4/Figure 4D/Fig.4D_HA/IAV_004_RAW_ch01.tif]

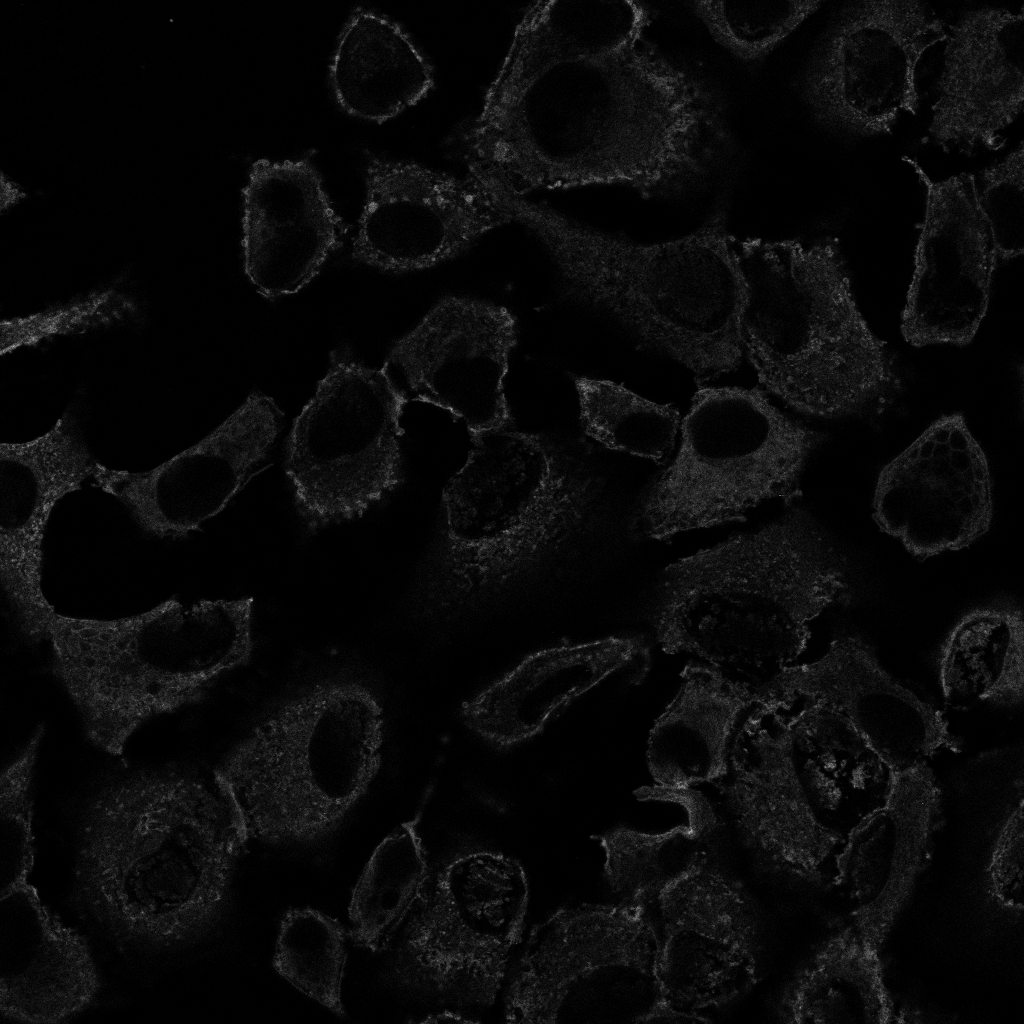

Supplement: Supplementary file 5 — Source data Fig. 4 [file 44319_2025_498_MOESM5_ESM.zip › Figure 4/Figure 4D/Fig.4D_HA/IAV_004_RAW_ch02.tif]

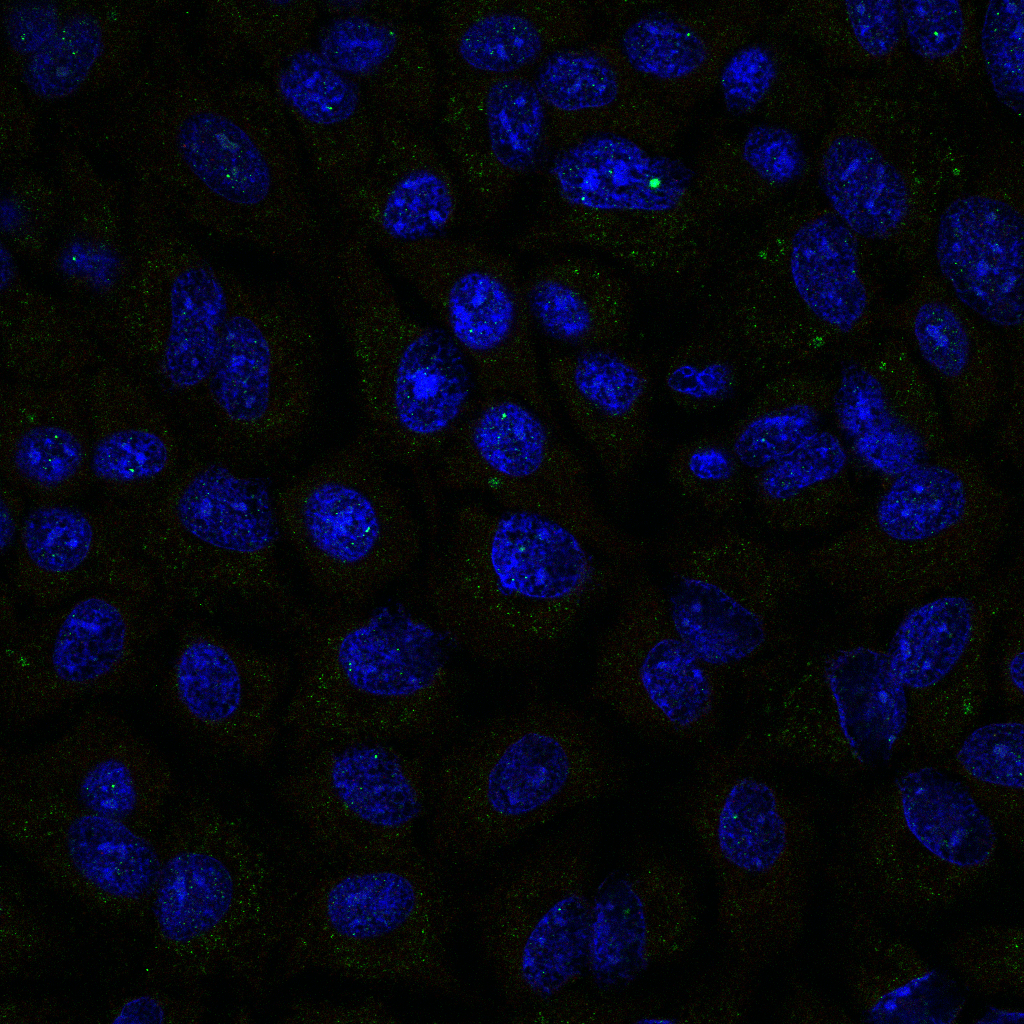

Supplement: Supplementary file 5 — Source data Fig. 4 [file 44319_2025_498_MOESM5_ESM.zip › Figure 4/Figure 4D/Fig.4D_HA/Mock_003.tif]

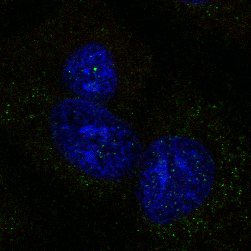

Supplement: Supplementary file 5 — Source data Fig. 4 [file 44319_2025_498_MOESM5_ESM.zip › Figure 4/Figure 4D/Fig.4D_HA/Mock_003_Crop001.tif]

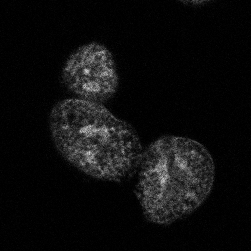

Supplement: Supplementary file 5 — Source data Fig. 4 [file 44319_2025_498_MOESM5_ESM.zip › Figure 4/Figure 4D/Fig.4D_HA/Mock_003_Crop001_RAW_ch00.tif]

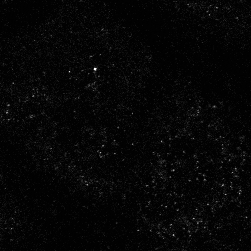

Supplement: Supplementary file 5 — Source data Fig. 4 [file 44319_2025_498_MOESM5_ESM.zip › Figure 4/Figure 4D/Fig.4D_HA/Mock_003_Crop001_RAW_ch01.tif]

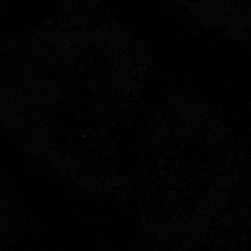

Supplement: Supplementary file 5 — Source data Fig. 4 [file 44319_2025_498_MOESM5_ESM.zip › Figure 4/Figure 4D/Fig.4D_HA/Mock_003_Crop001_RAW_ch02.tif]

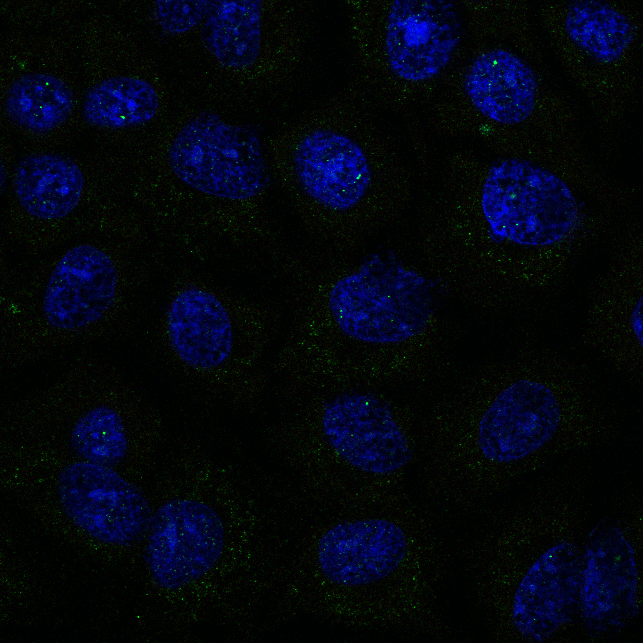

Supplement: Supplementary file 5 — Source data Fig. 4 [file 44319_2025_498_MOESM5_ESM.zip › Figure 4/Figure 4D/Fig.4D_HA/Mock_003_Crop002.tif]

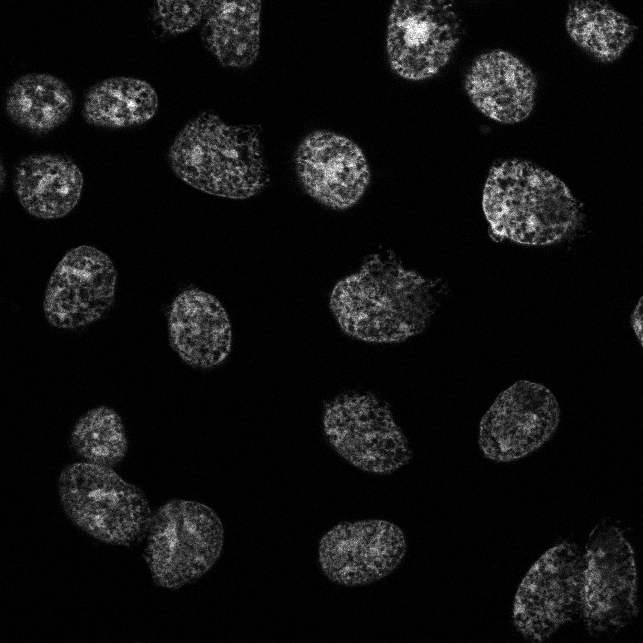

Supplement: Supplementary file 5 — Source data Fig. 4 [file 44319_2025_498_MOESM5_ESM.zip › Figure 4/Figure 4D/Fig.4D_HA/Mock_003_Crop002_RAW_ch00.tif]

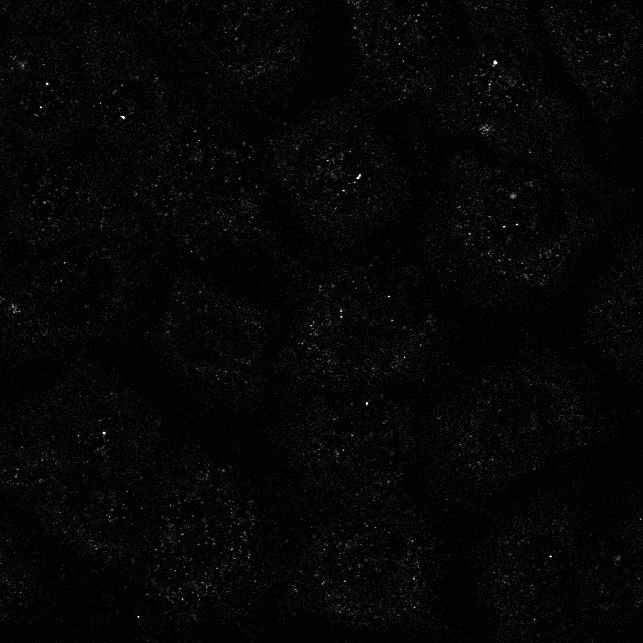

Supplement: Supplementary file 5 — Source data Fig. 4 [file 44319_2025_498_MOESM5_ESM.zip › Figure 4/Figure 4D/Fig.4D_HA/Mock_003_Crop002_RAW_ch01.tif]

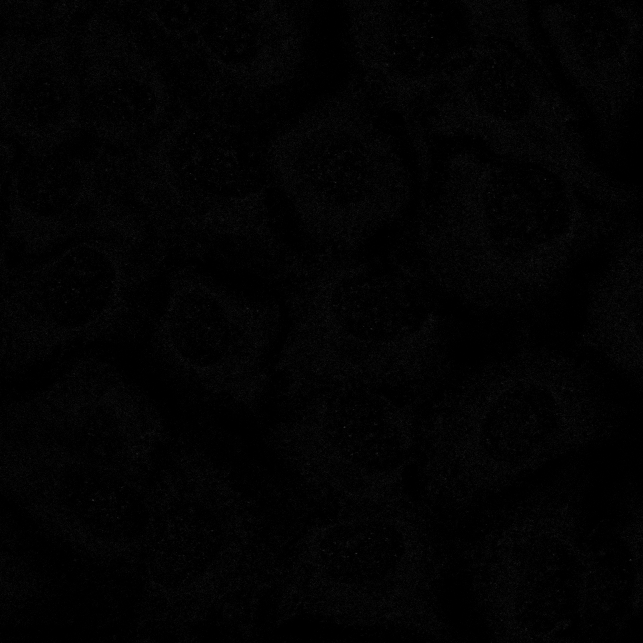

Supplement: Supplementary file 5 — Source data Fig. 4 [file 44319_2025_498_MOESM5_ESM.zip › Figure 4/Figure 4D/Fig.4D_HA/Mock_003_Crop002_RAW_ch02.tif]

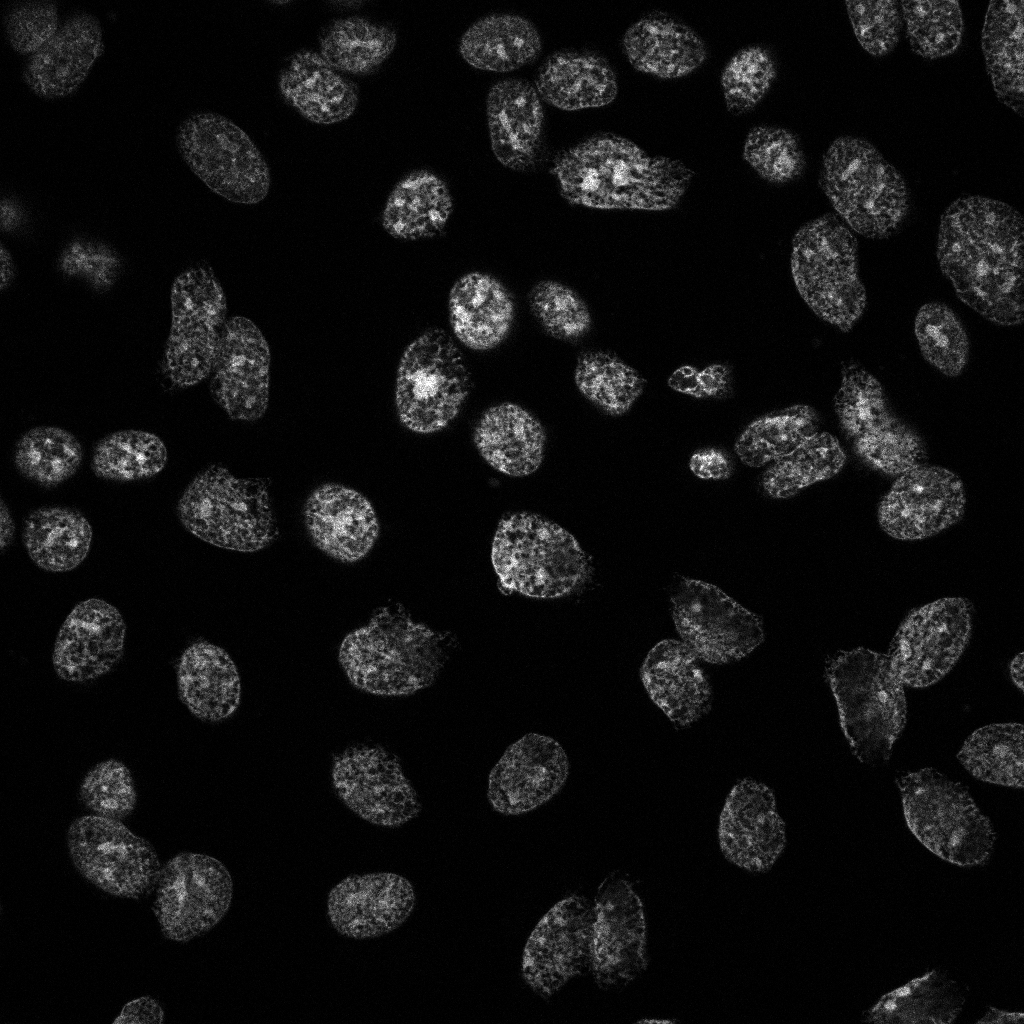

Supplement: Supplementary file 5 — Source data Fig. 4 [file 44319_2025_498_MOESM5_ESM.zip › Figure 4/Figure 4D/Fig.4D_HA/Mock_003_RAW_ch00.tif]

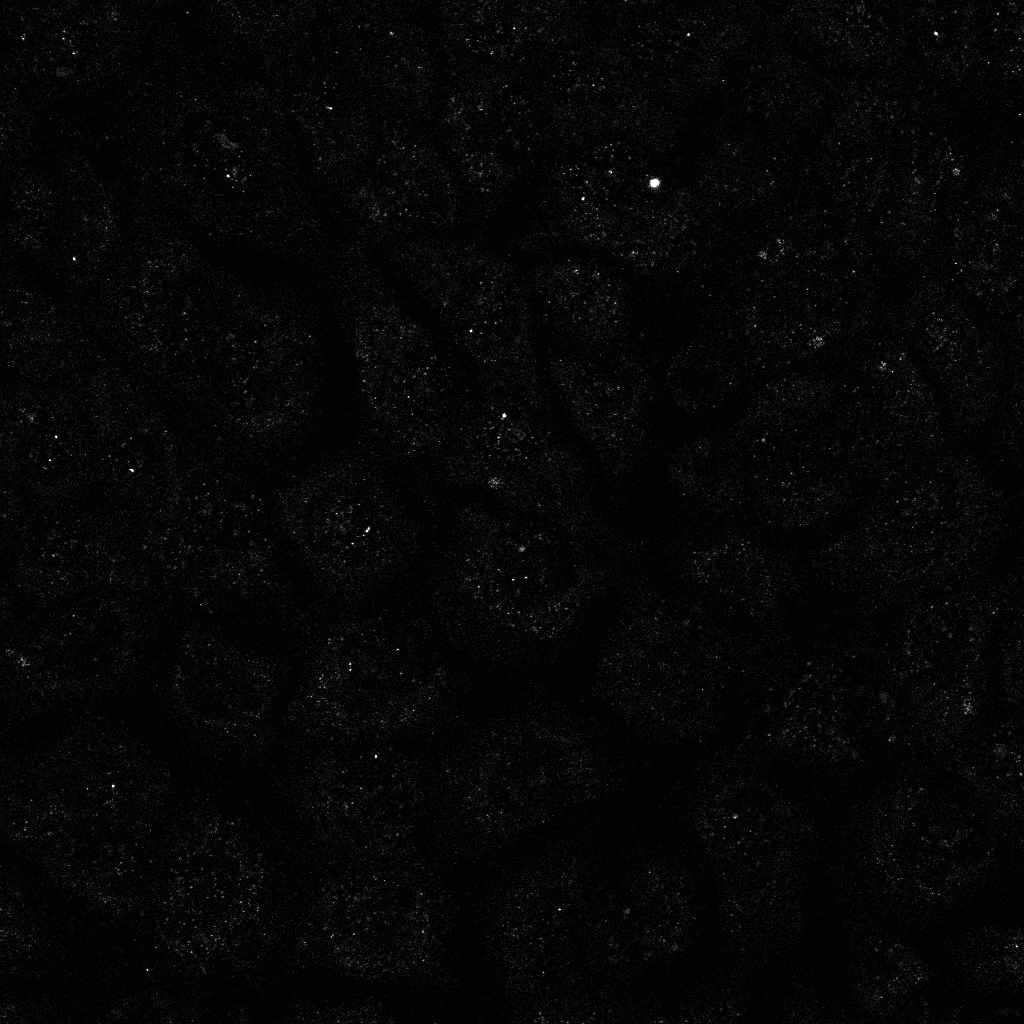

Supplement: Supplementary file 5 — Source data Fig. 4 [file 44319_2025_498_MOESM5_ESM.zip › Figure 4/Figure 4D/Fig.4D_HA/Mock_003_RAW_ch01.tif]

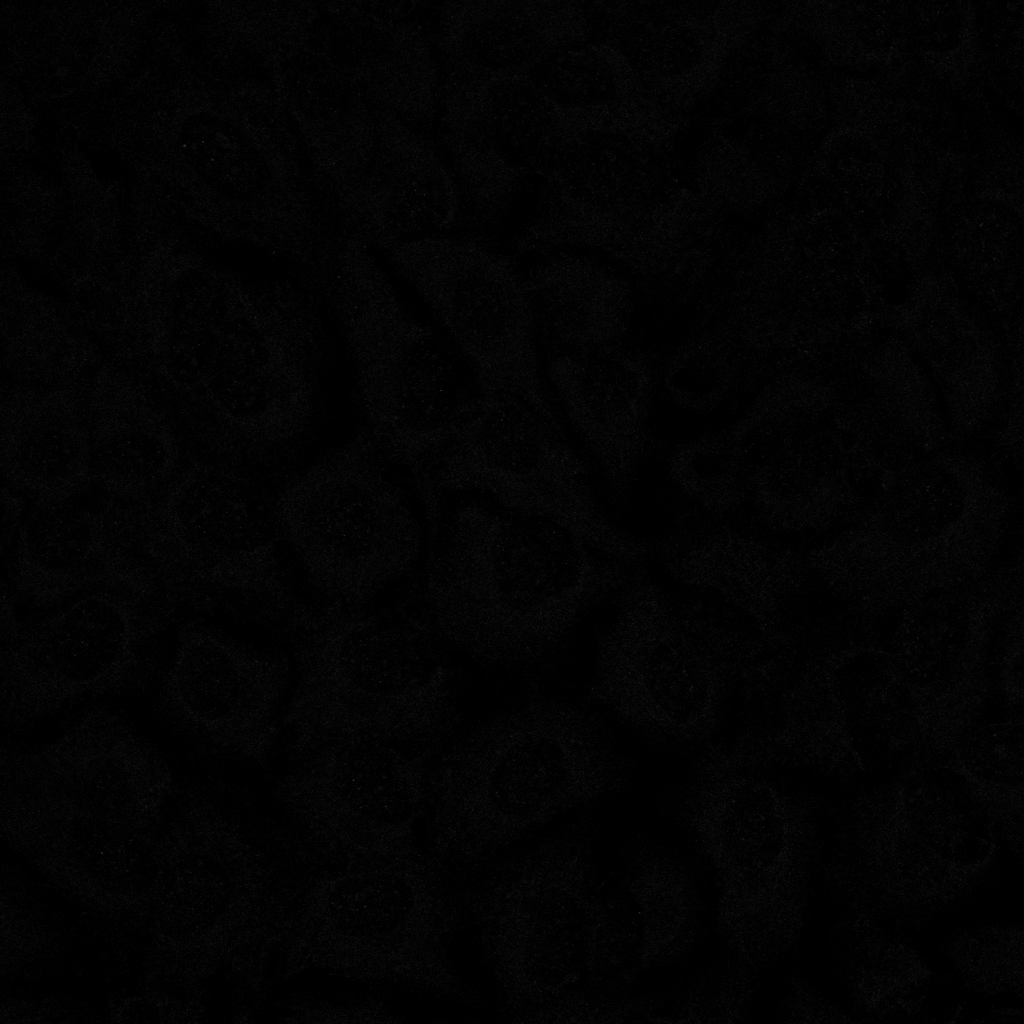

Supplement: Supplementary file 5 — Source data Fig. 4 [file 44319_2025_498_MOESM5_ESM.zip › Figure 4/Figure 4D/Fig.4D_HA/Mock_003_RAW_ch02.tif]

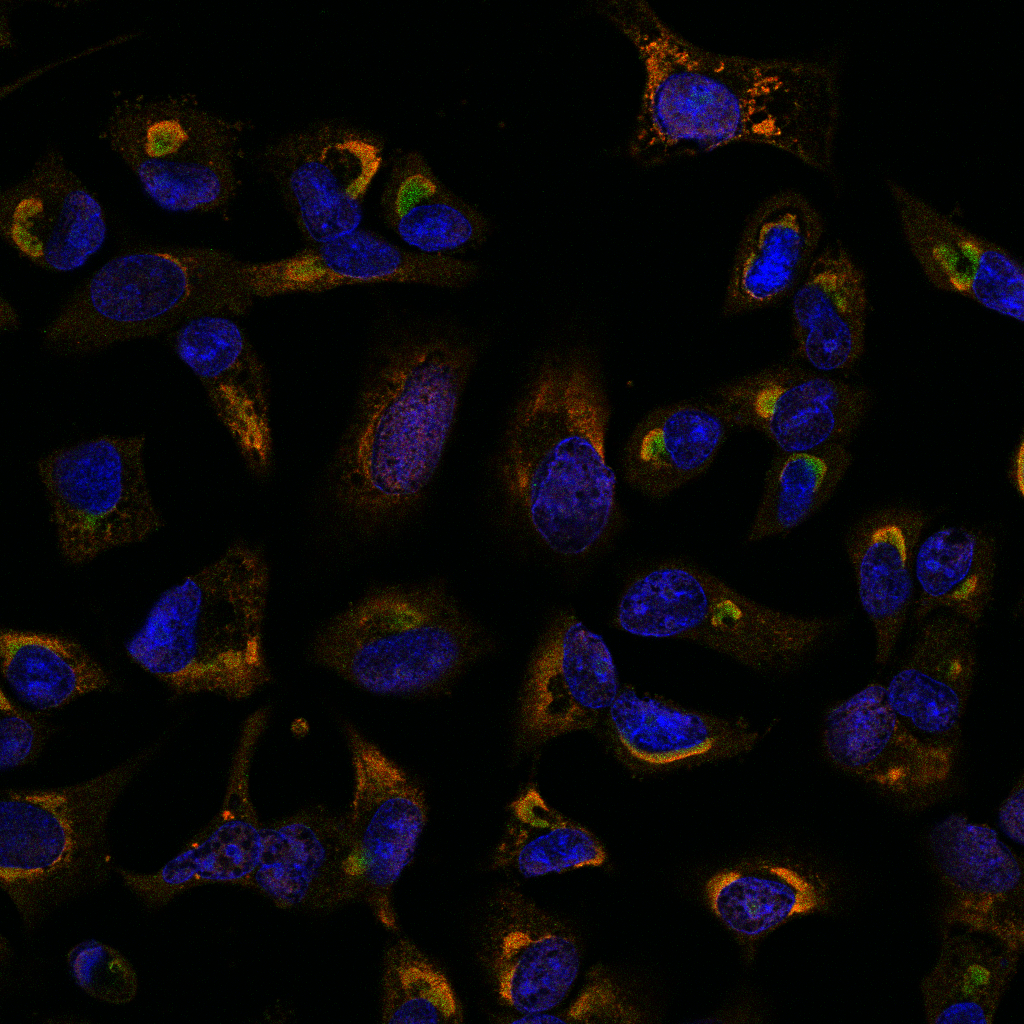

Supplement: Supplementary file 5 — Source data Fig. 4 [file 44319_2025_498_MOESM5_ESM.zip › Figure 4/Figure 4D/Fig.4D_NS1/IAV_003_000.tif]

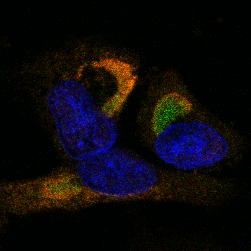

Supplement: Supplementary file 5 — Source data Fig. 4 [file 44319_2025_498_MOESM5_ESM.zip › Figure 4/Figure 4D/Fig.4D_NS1/IAV_003_000_Crop001.tif]

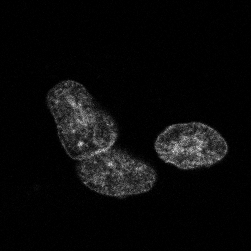

Supplement: Supplementary file 5 — Source data Fig. 4 [file 44319_2025_498_MOESM5_ESM.zip › Figure 4/Figure 4D/Fig.4D_NS1/IAV_003_000_Crop001_RAW_ch00.tif]

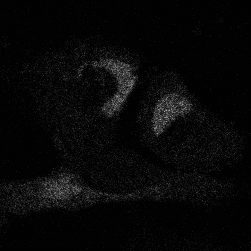

Supplement: Supplementary file 5 — Source data Fig. 4 [file 44319_2025_498_MOESM5_ESM.zip › Figure 4/Figure 4D/Fig.4D_NS1/IAV_003_000_Crop001_RAW_ch01.tif]

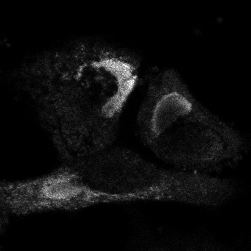

Supplement: Supplementary file 5 — Source data Fig. 4 [file 44319_2025_498_MOESM5_ESM.zip › Figure 4/Figure 4D/Fig.4D_NS1/IAV_003_000_Crop001_RAW_ch02.tif]

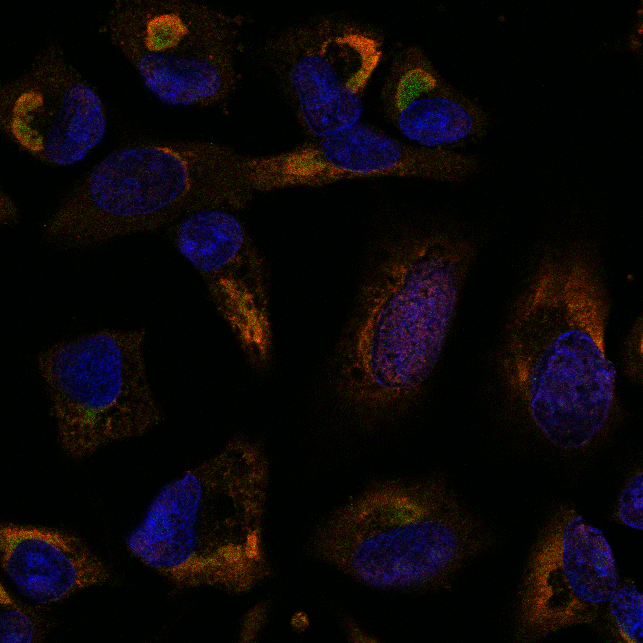

Supplement: Supplementary file 5 — Source data Fig. 4 [file 44319_2025_498_MOESM5_ESM.zip › Figure 4/Figure 4D/Fig.4D_NS1/IAV_003_000_Crop002.tif]

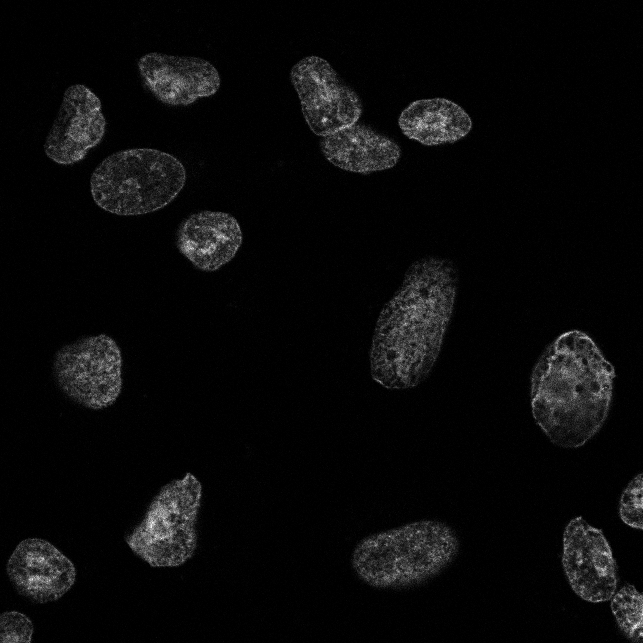

Supplement: Supplementary file 5 — Source data Fig. 4 [file 44319_2025_498_MOESM5_ESM.zip › Figure 4/Figure 4D/Fig.4D_NS1/IAV_003_000_Crop002_RAW_ch00.tif]

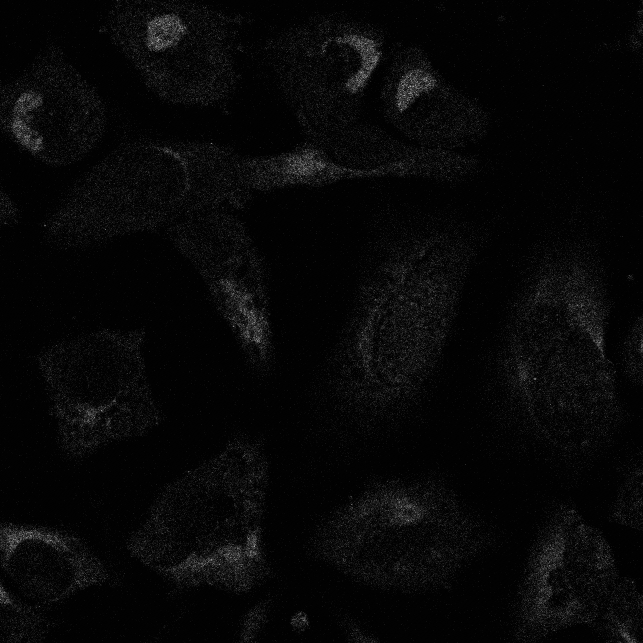

Supplement: Supplementary file 5 — Source data Fig. 4 [file 44319_2025_498_MOESM5_ESM.zip › Figure 4/Figure 4D/Fig.4D_NS1/IAV_003_000_Crop002_RAW_ch01.tif]

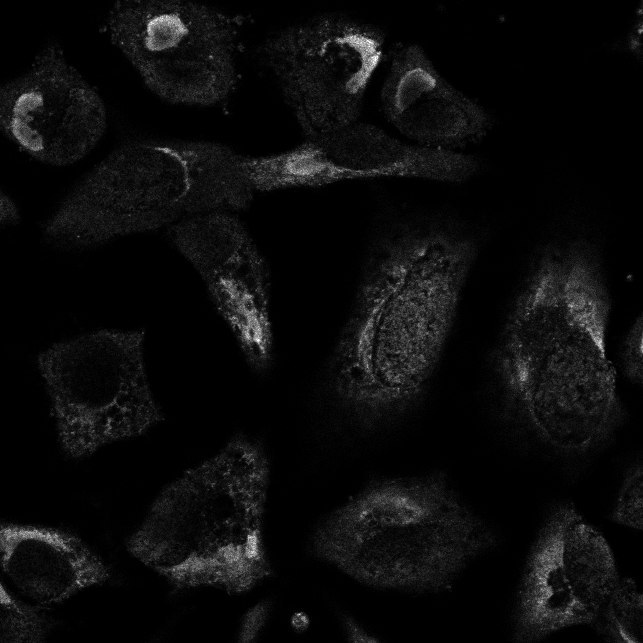

Supplement: Supplementary file 5 — Source data Fig. 4 [file 44319_2025_498_MOESM5_ESM.zip › Figure 4/Figure 4D/Fig.4D_NS1/IAV_003_000_Crop002_RAW_ch02.tif]

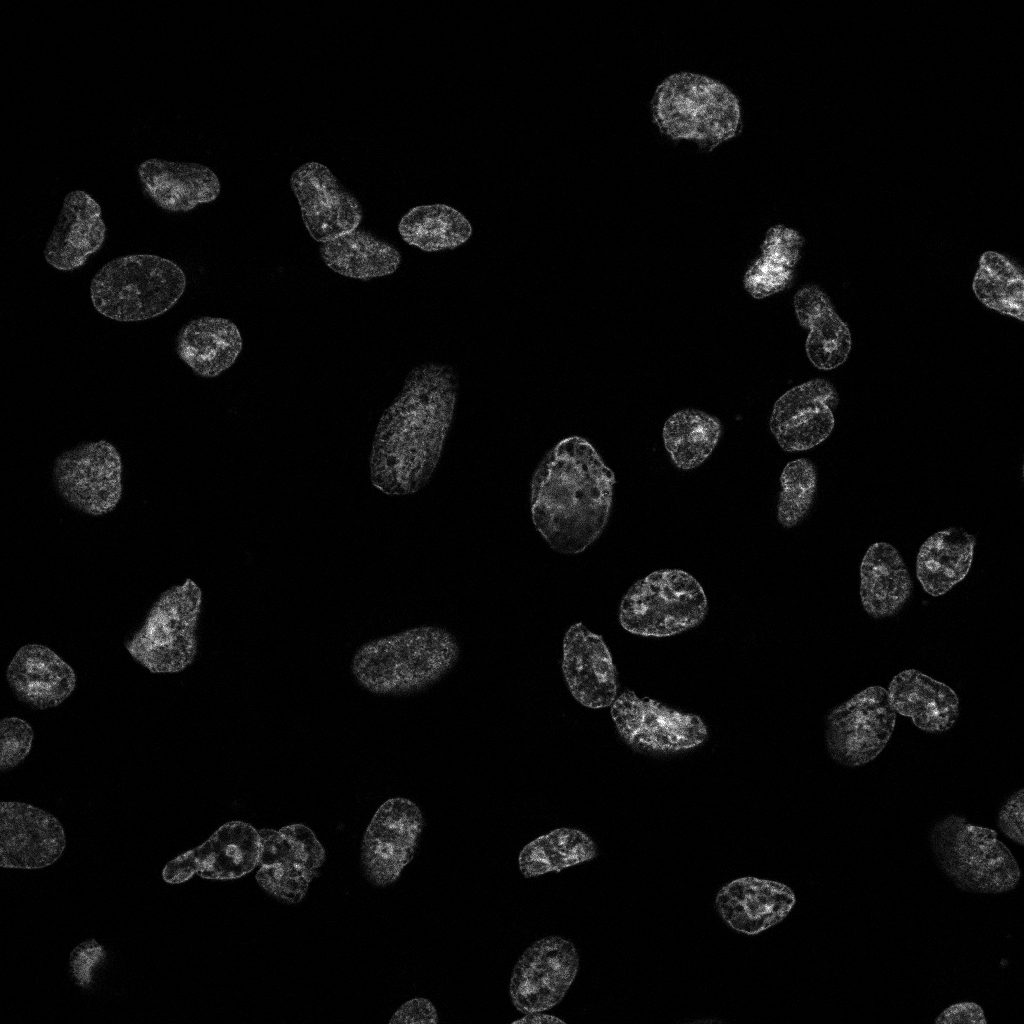

Supplement: Supplementary file 5 — Source data Fig. 4 [file 44319_2025_498_MOESM5_ESM.zip › Figure 4/Figure 4D/Fig.4D_NS1/IAV_003_000_RAW_ch00.tif]

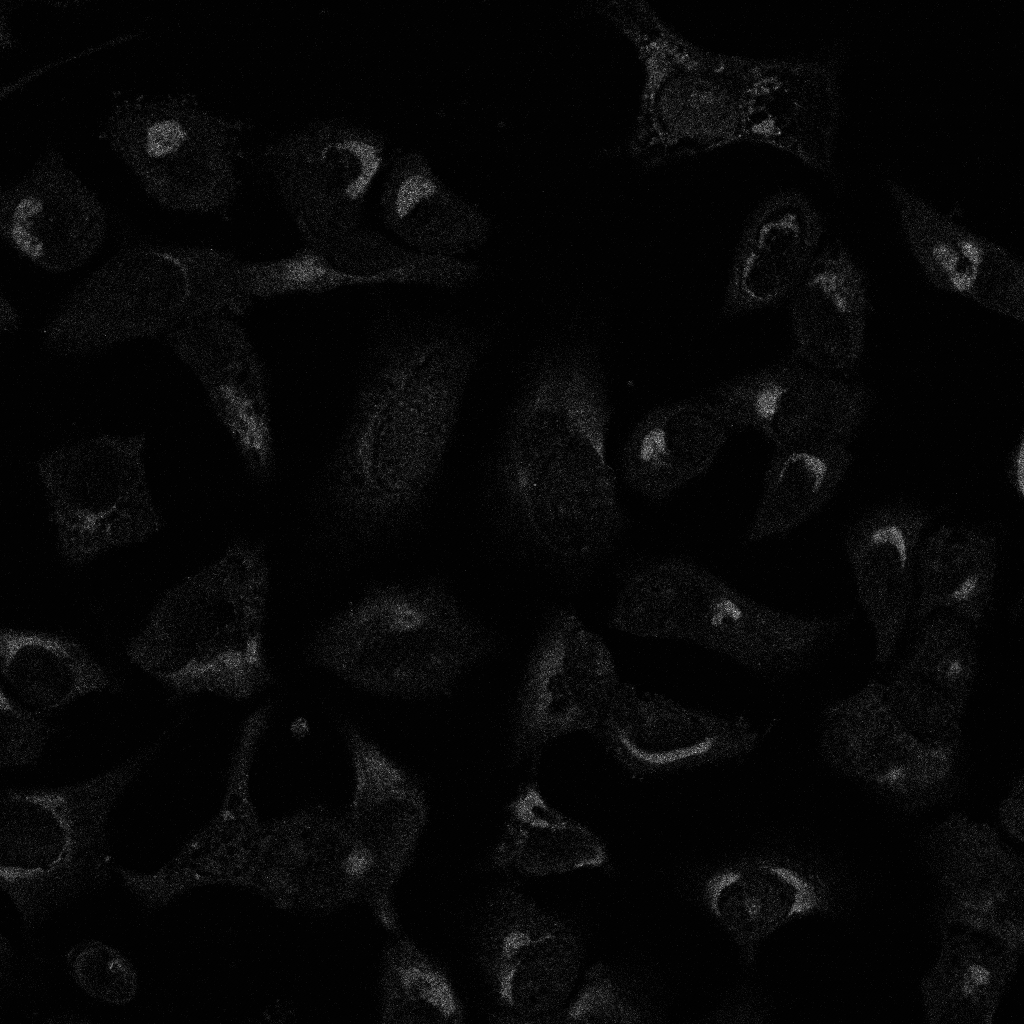

Supplement: Supplementary file 5 — Source data Fig. 4 [file 44319_2025_498_MOESM5_ESM.zip › Figure 4/Figure 4D/Fig.4D_NS1/IAV_003_000_RAW_ch01.tif]

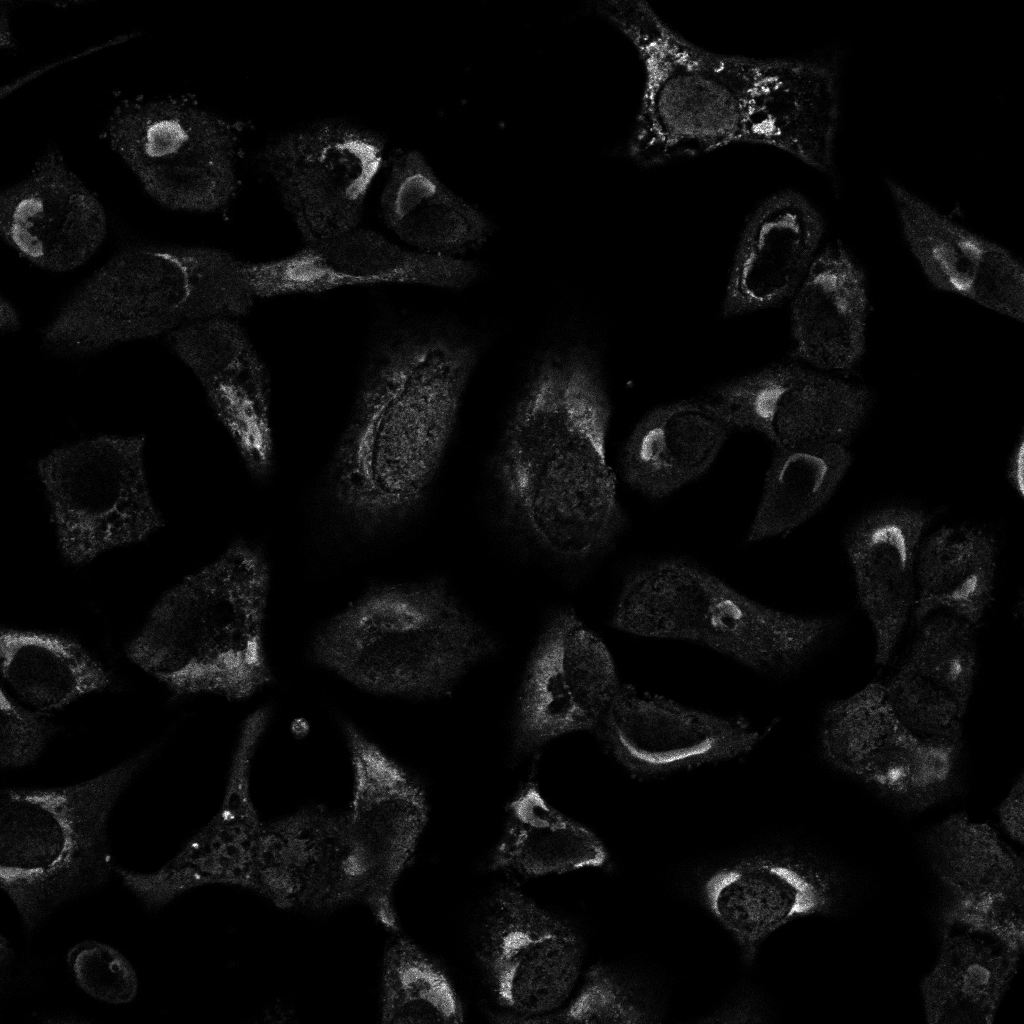

Supplement: Supplementary file 5 — Source data Fig. 4 [file 44319_2025_498_MOESM5_ESM.zip › Figure 4/Figure 4D/Fig.4D_NS1/IAV_003_000_RAW_ch02.tif]

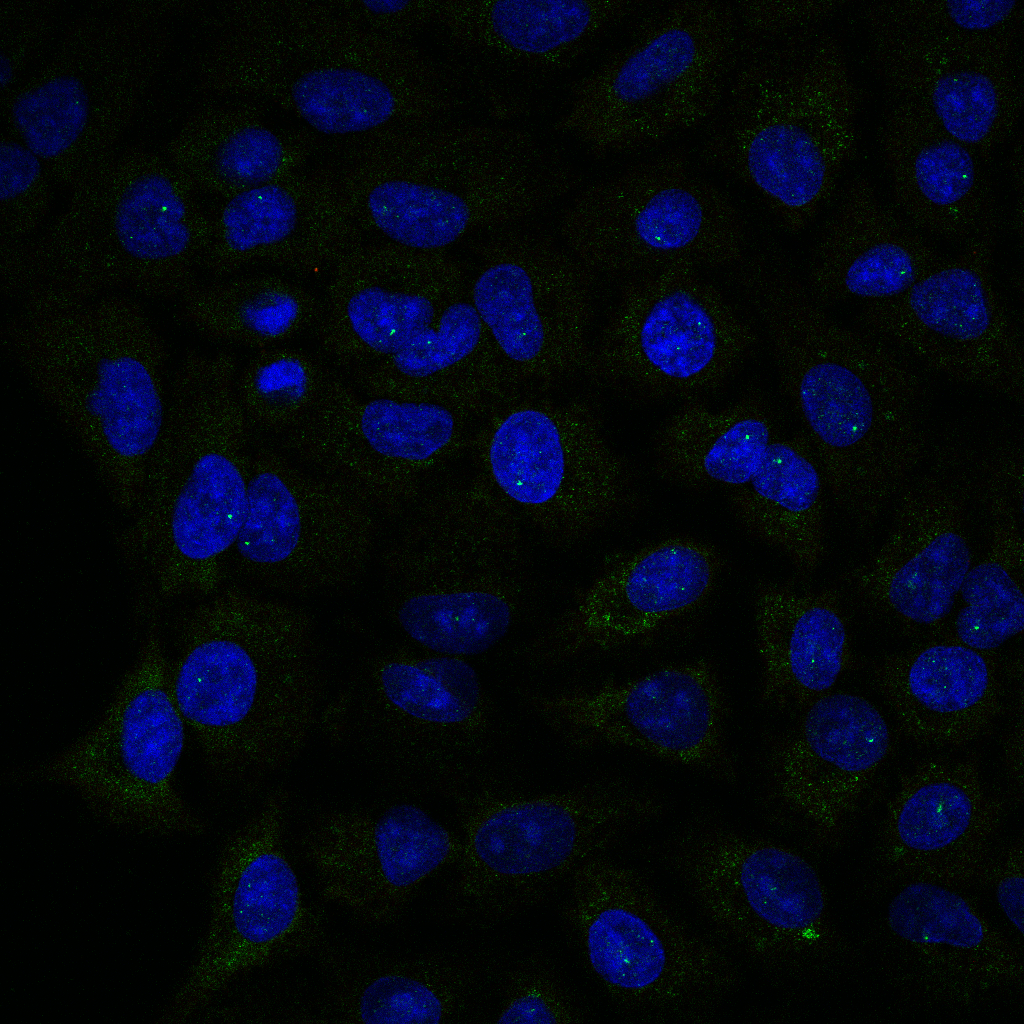

Supplement: Supplementary file 5 — Source data Fig. 4 [file 44319_2025_498_MOESM5_ESM.zip › Figure 4/Figure 4D/Fig.4D_NS1/Mock_001.tif]

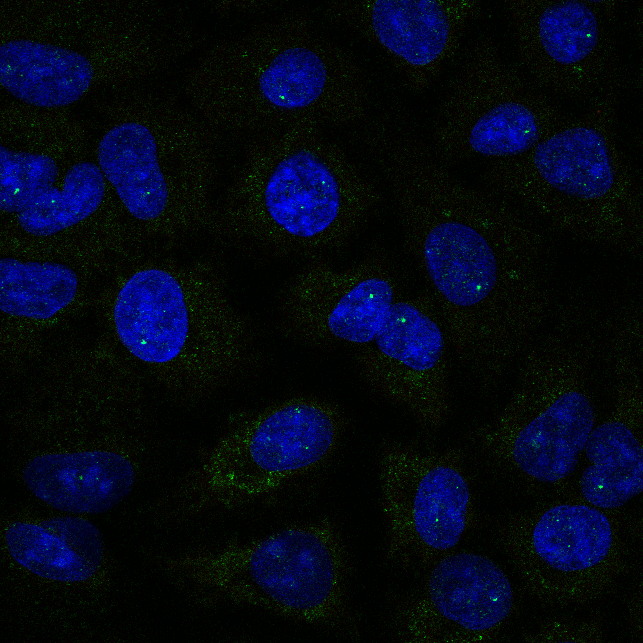

Supplement: Supplementary file 5 — Source data Fig. 4 [file 44319_2025_498_MOESM5_ESM.zip › Figure 4/Figure 4D/Fig.4D_NS1/Mock_001_Crop003.tif]

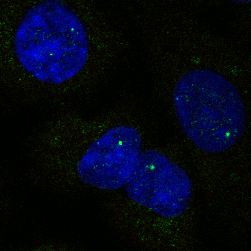

Supplement: Supplementary file 5 — Source data Fig. 4 [file 44319_2025_498_MOESM5_ESM.zip › Figure 4/Figure 4D/Fig.4D_NS1/Mock_001_Crop003_Crop001.tif]

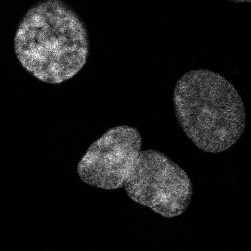

Supplement: Supplementary file 5 — Source data Fig. 4 [file 44319_2025_498_MOESM5_ESM.zip › Figure 4/Figure 4D/Fig.4D_NS1/Mock_001_Crop003_Crop001_RAW_ch00.tif]

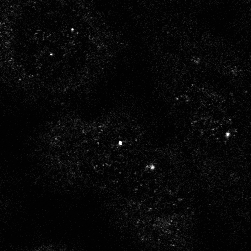

Supplement: Supplementary file 5 — Source data Fig. 4 [file 44319_2025_498_MOESM5_ESM.zip › Figure 4/Figure 4D/Fig.4D_NS1/Mock_001_Crop003_Crop001_RAW_ch01.tif]

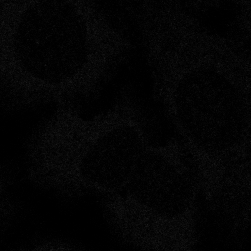

Supplement: Supplementary file 5 — Source data Fig. 4 [file 44319_2025_498_MOESM5_ESM.zip › Figure 4/Figure 4D/Fig.4D_NS1/Mock_001_Crop003_Crop001_RAW_ch02.tif]

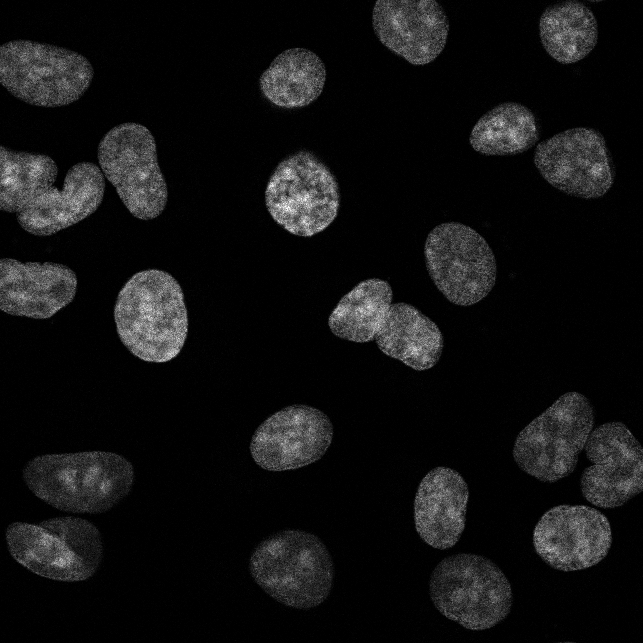

Supplement: Supplementary file 5 — Source data Fig. 4 [file 44319_2025_498_MOESM5_ESM.zip › Figure 4/Figure 4D/Fig.4D_NS1/Mock_001_Crop003_RAW_ch00.tif]

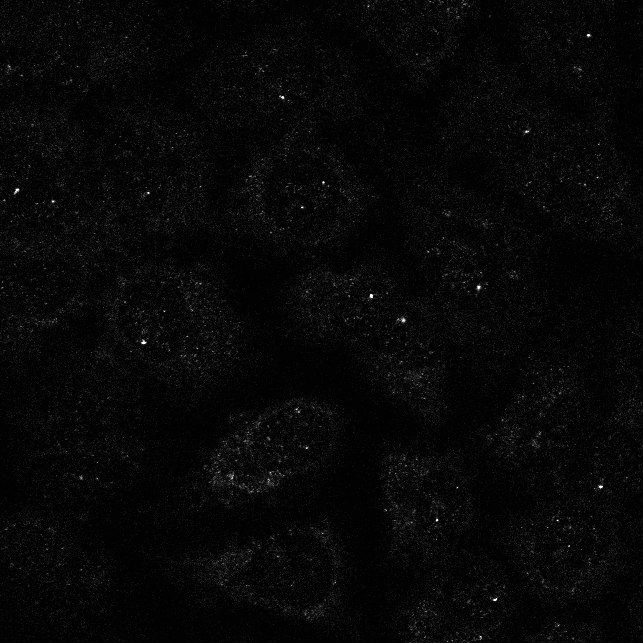

Supplement: Supplementary file 5 — Source data Fig. 4 [file 44319_2025_498_MOESM5_ESM.zip › Figure 4/Figure 4D/Fig.4D_NS1/Mock_001_Crop003_RAW_ch01.tif]

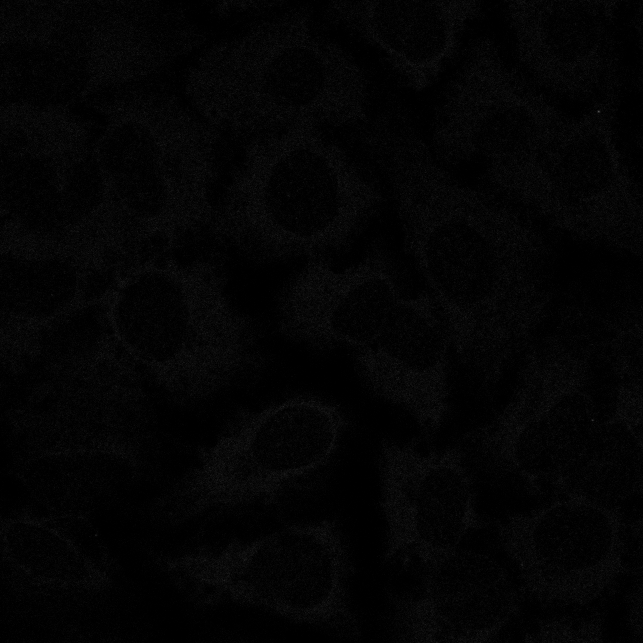

Supplement: Supplementary file 5 — Source data Fig. 4 [file 44319_2025_498_MOESM5_ESM.zip › Figure 4/Figure 4D/Fig.4D_NS1/Mock_001_Crop003_RAW_ch02.tif]

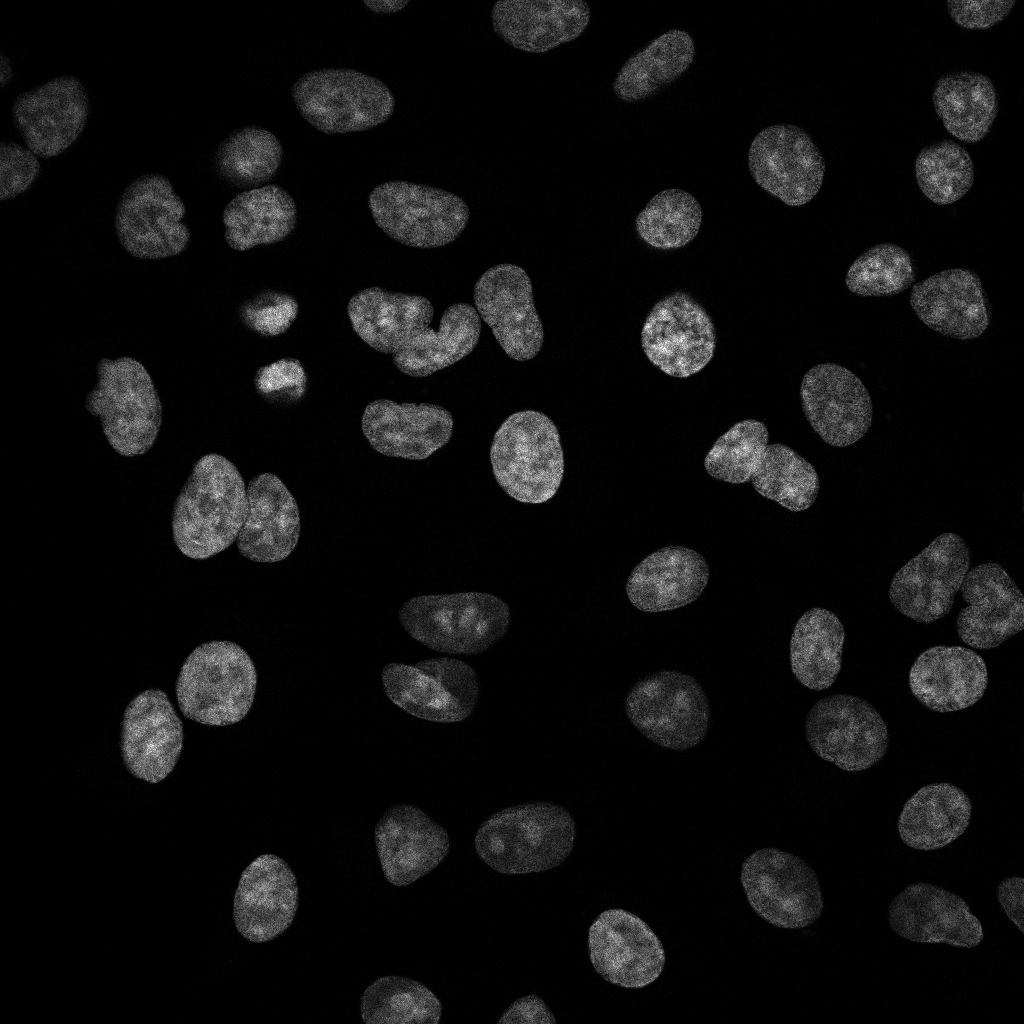

Supplement: Supplementary file 5 — Source data Fig. 4 [file 44319_2025_498_MOESM5_ESM.zip › Figure 4/Figure 4D/Fig.4D_NS1/Mock_001_RAW_ch00.tif]

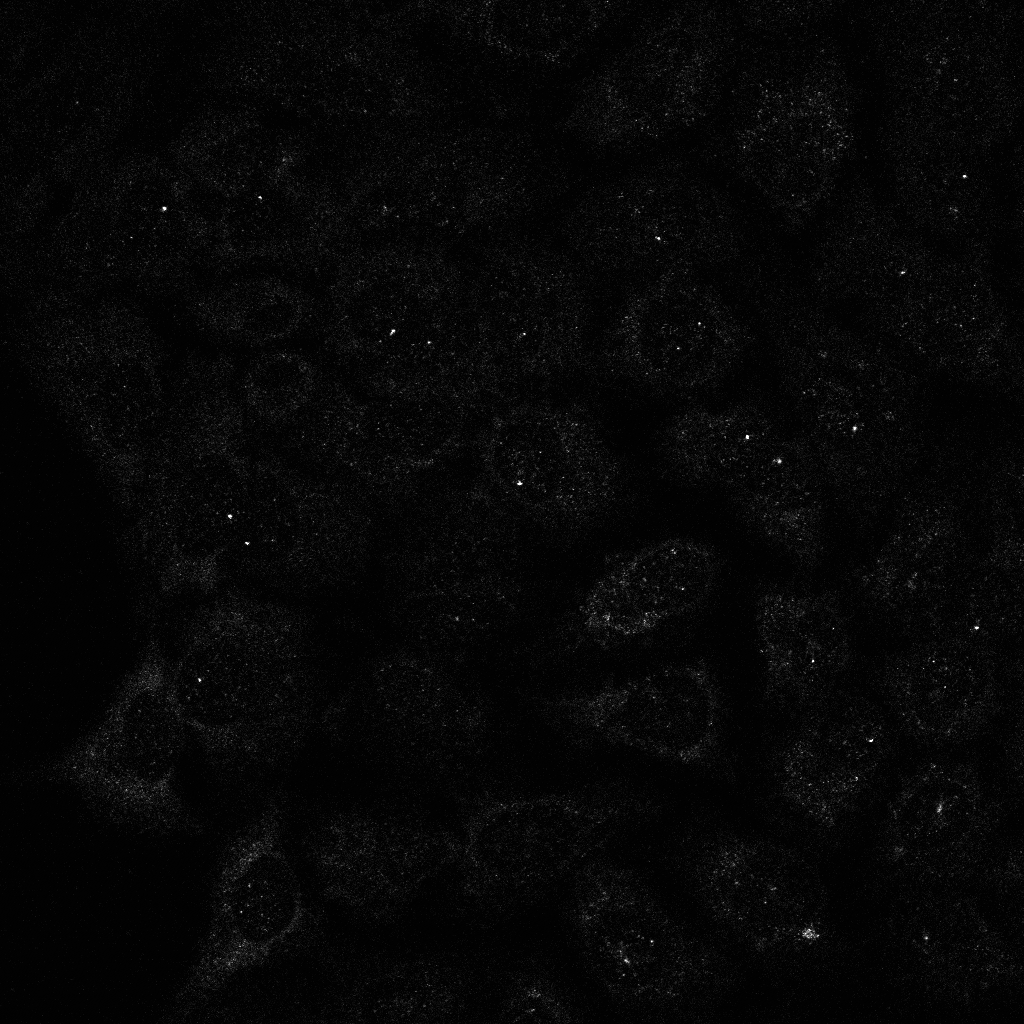

Supplement: Supplementary file 5 — Source data Fig. 4 [file 44319_2025_498_MOESM5_ESM.zip › Figure 4/Figure 4D/Fig.4D_NS1/Mock_001_RAW_ch01.tif]

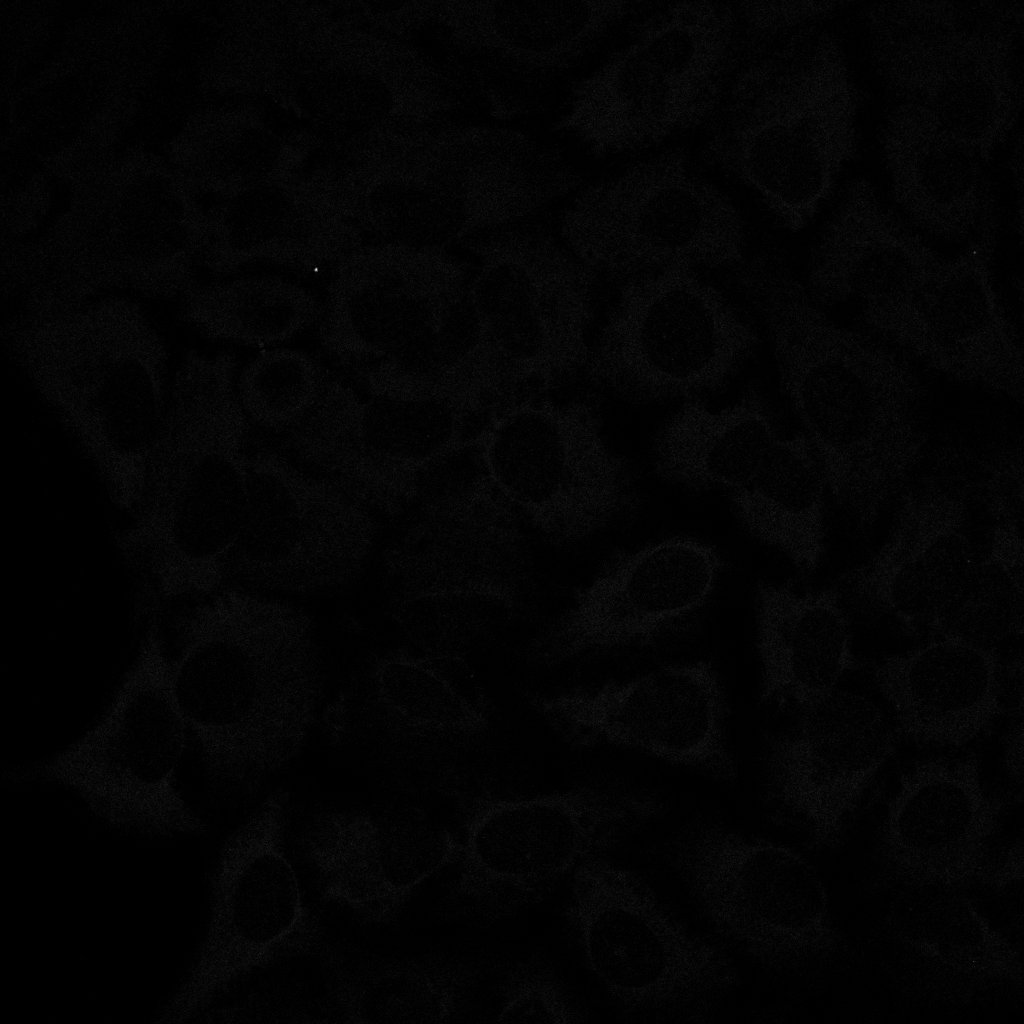

Supplement: Supplementary file 5 — Source data Fig. 4 [file 44319_2025_498_MOESM5_ESM.zip › Figure 4/Figure 4D/Fig.4D_NS1/Mock_001_RAW_ch02.tif]

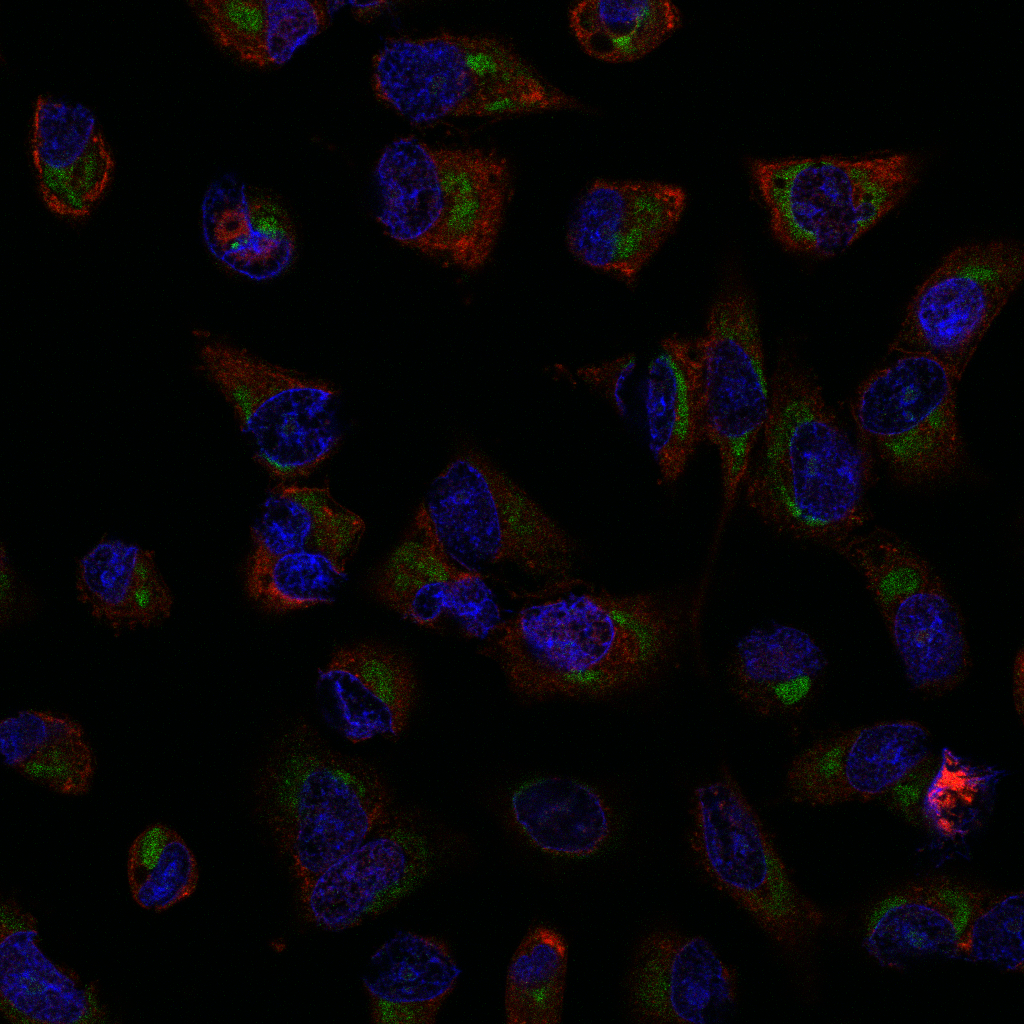

Supplement: Supplementary file 5 — Source data Fig. 4 [file 44319_2025_498_MOESM5_ESM.zip › Figure 4/Figure 4D/Fig.4D_PB2/IAV_003.tif]

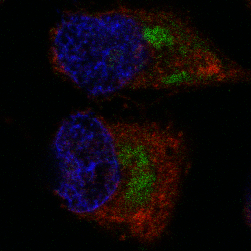

Supplement: Supplementary file 5 — Source data Fig. 4 [file 44319_2025_498_MOESM5_ESM.zip › Figure 4/Figure 4D/Fig.4D_PB2/IAV_003_Crop001.tif]

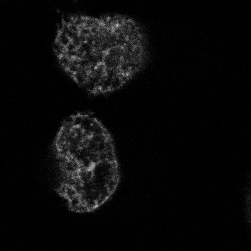

Supplement: Supplementary file 5 — Source data Fig. 4 [file 44319_2025_498_MOESM5_ESM.zip › Figure 4/Figure 4D/Fig.4D_PB2/IAV_003_Crop001_RAW_ch00.tif]

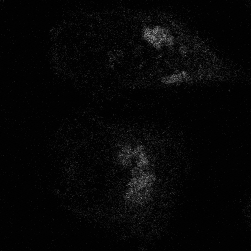

Supplement: Supplementary file 5 — Source data Fig. 4 [file 44319_2025_498_MOESM5_ESM.zip › Figure 4/Figure 4D/Fig.4D_PB2/IAV_003_Crop001_RAW_ch01.tif]

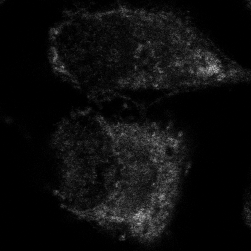

Supplement: Supplementary file 5 — Source data Fig. 4 [file 44319_2025_498_MOESM5_ESM.zip › Figure 4/Figure 4D/Fig.4D_PB2/IAV_003_Crop001_RAW_ch02.tif]

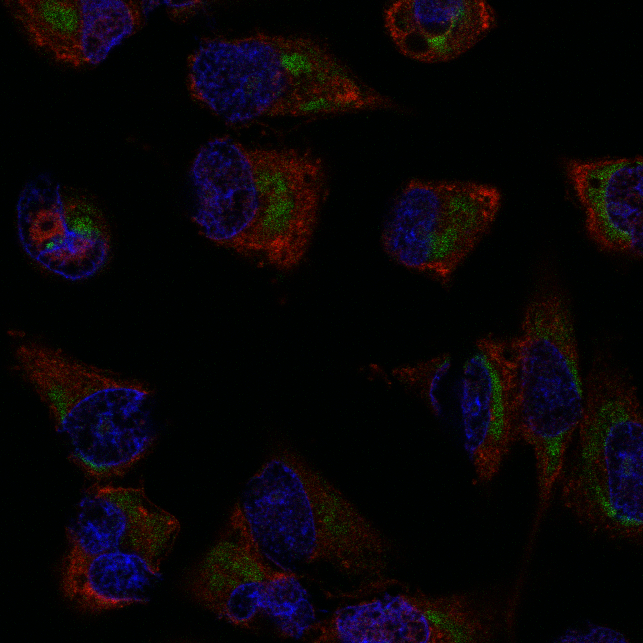

Supplement: Supplementary file 5 — Source data Fig. 4 [file 44319_2025_498_MOESM5_ESM.zip › Figure 4/Figure 4D/Fig.4D_PB2/IAV_003_Crop002.tif]

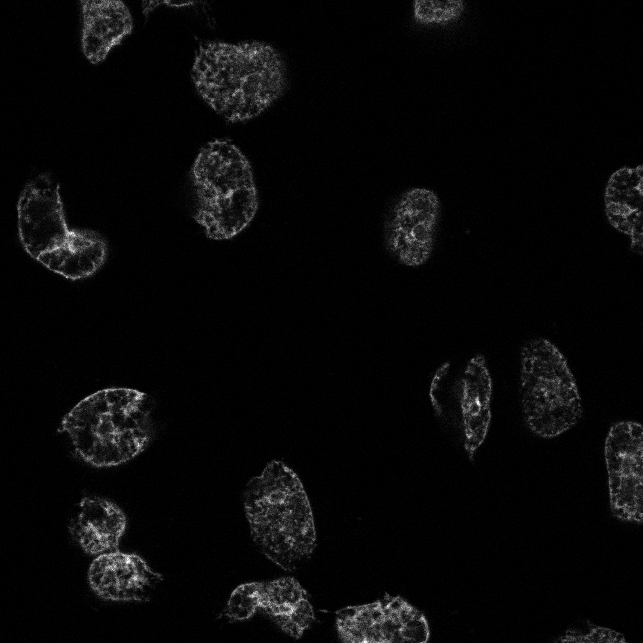

Supplement: Supplementary file 5 — Source data Fig. 4 [file 44319_2025_498_MOESM5_ESM.zip › Figure 4/Figure 4D/Fig.4D_PB2/IAV_003_Crop002_RAW_ch00.tif]

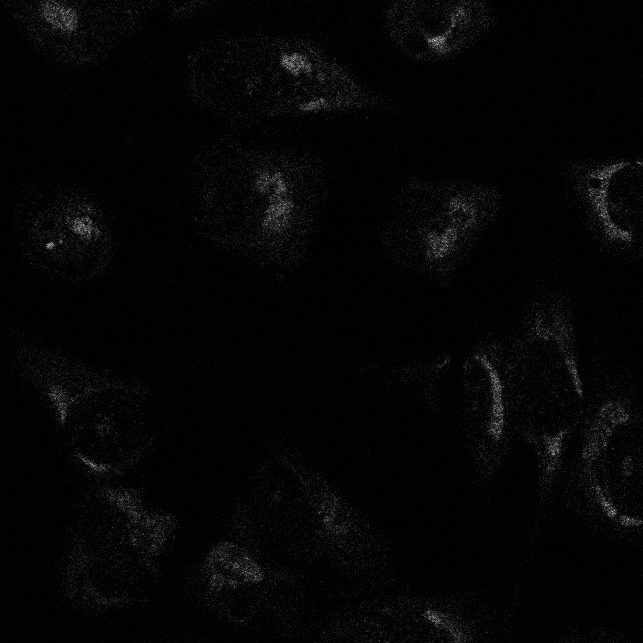

Supplement: Supplementary file 5 — Source data Fig. 4 [file 44319_2025_498_MOESM5_ESM.zip › Figure 4/Figure 4D/Fig.4D_PB2/IAV_003_Crop002_RAW_ch01.tif]

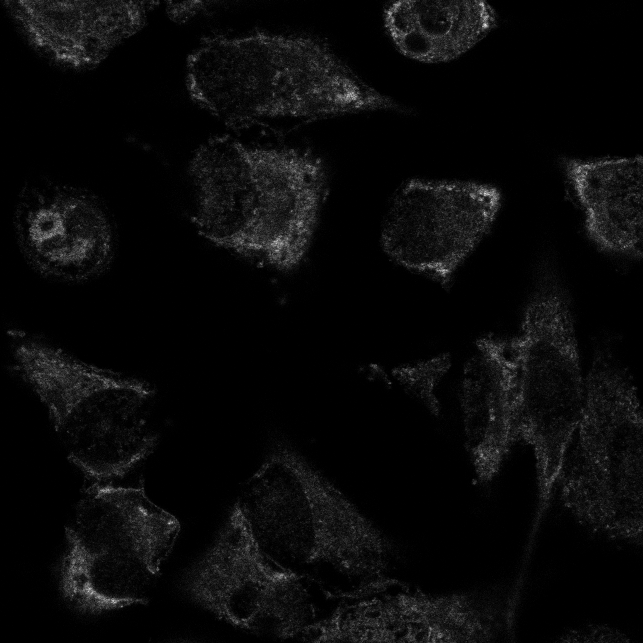

Supplement: Supplementary file 5 — Source data Fig. 4 [file 44319_2025_498_MOESM5_ESM.zip › Figure 4/Figure 4D/Fig.4D_PB2/IAV_003_Crop002_RAW_ch02.tif]

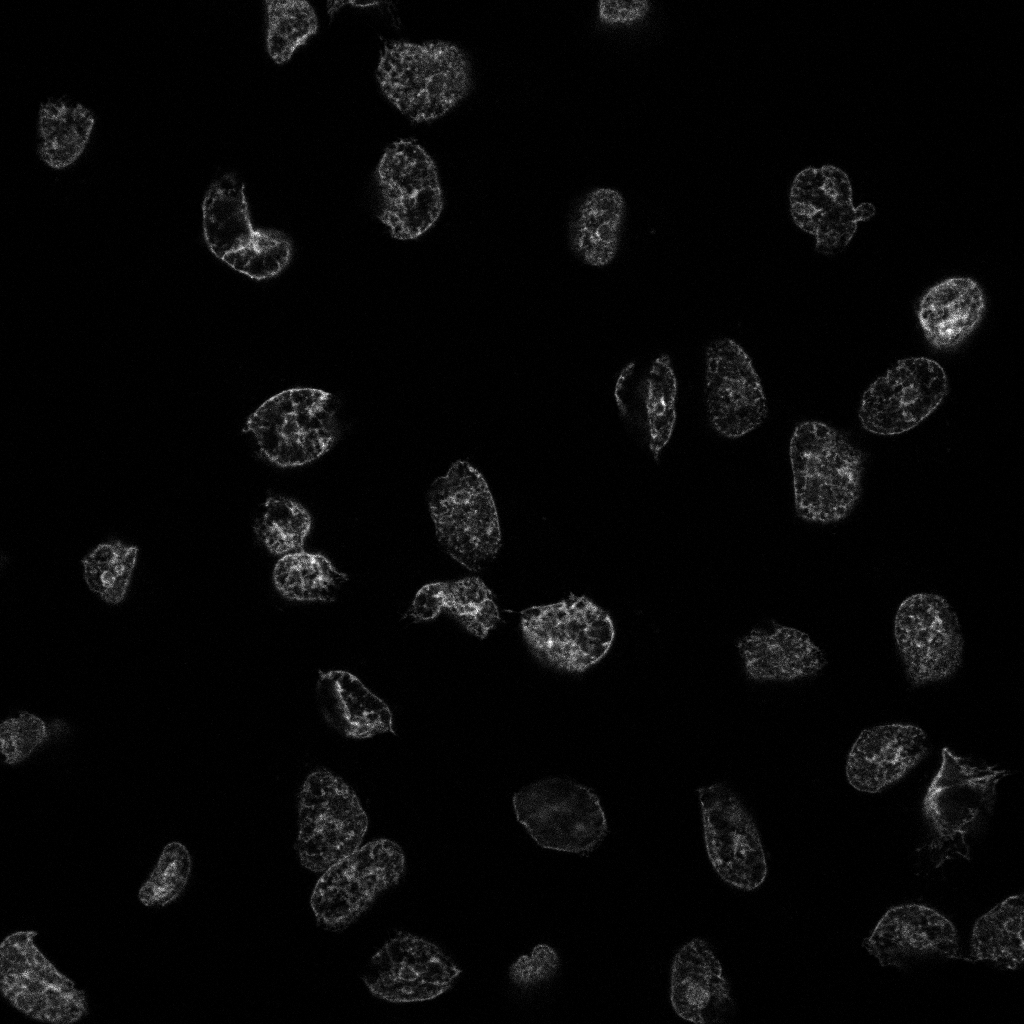

Supplement: Supplementary file 5 — Source data Fig. 4 [file 44319_2025_498_MOESM5_ESM.zip › Figure 4/Figure 4D/Fig.4D_PB2/IAV_003_RAW_ch00.tif]

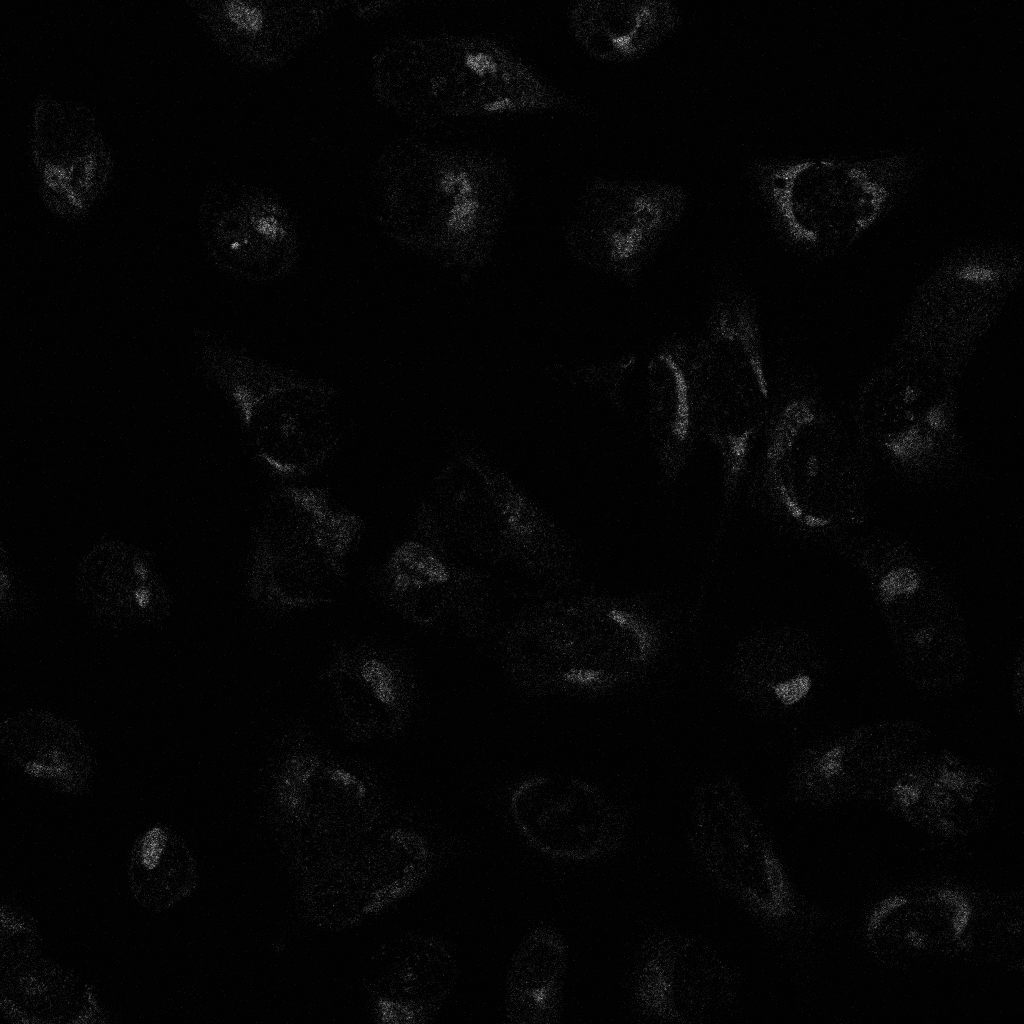

Supplement: Supplementary file 5 — Source data Fig. 4 [file 44319_2025_498_MOESM5_ESM.zip › Figure 4/Figure 4D/Fig.4D_PB2/IAV_003_RAW_ch01.tif]

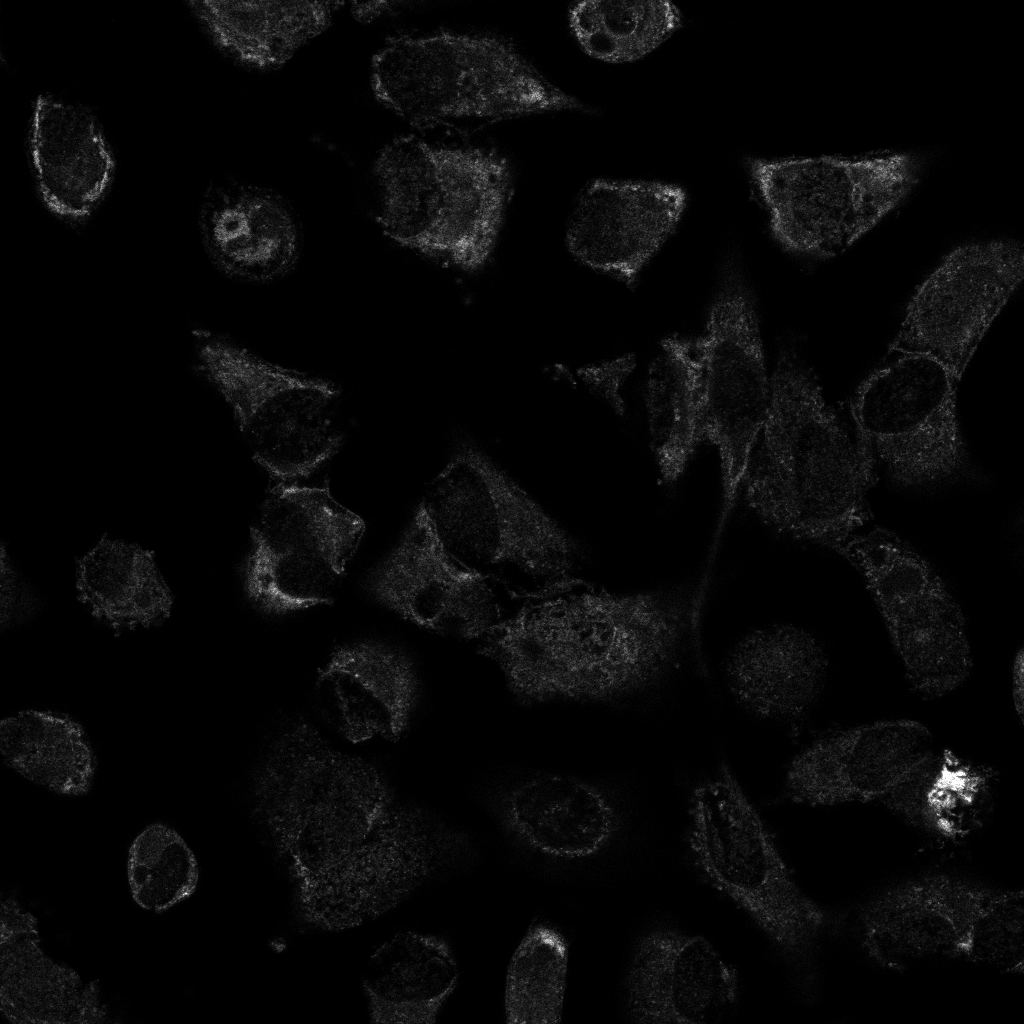

Supplement: Supplementary file 5 — Source data Fig. 4 [file 44319_2025_498_MOESM5_ESM.zip › Figure 4/Figure 4D/Fig.4D_PB2/IAV_003_RAW_ch02.tif]

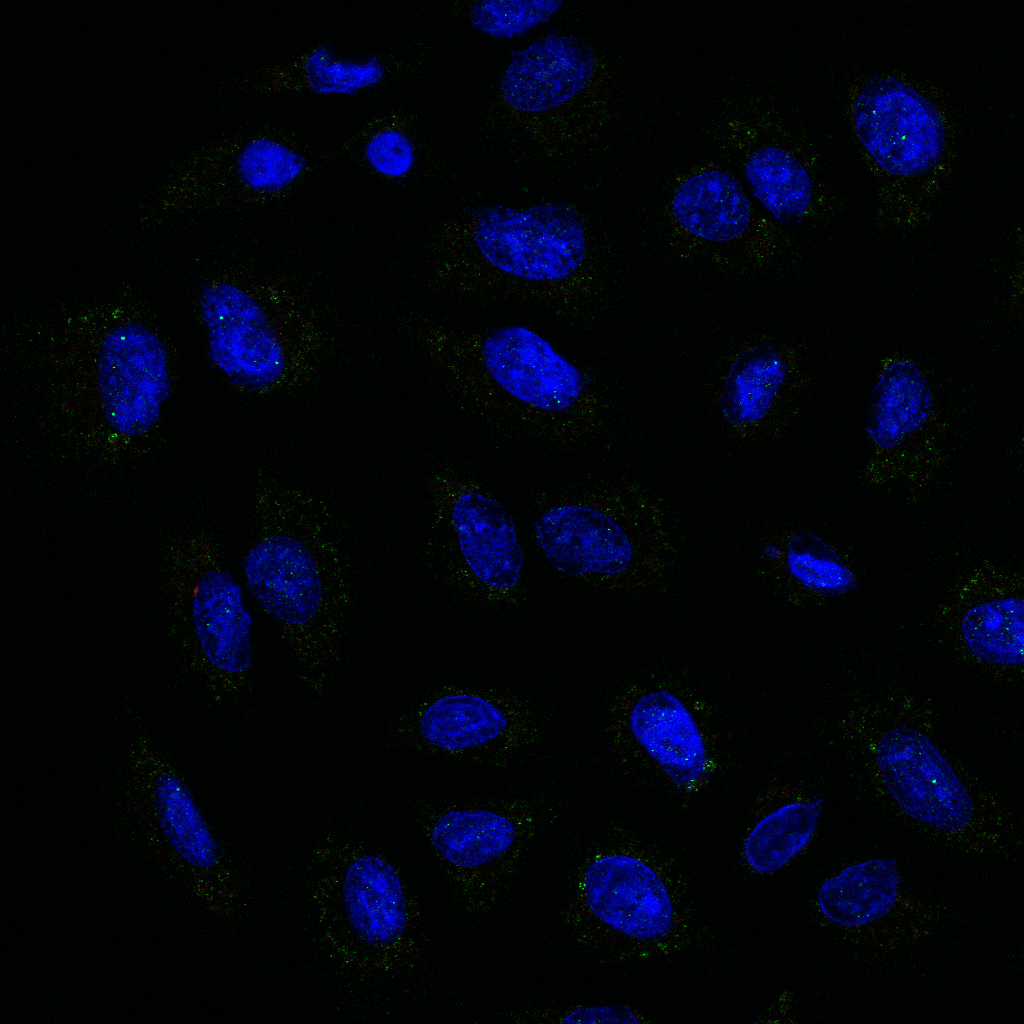

Supplement: Supplementary file 5 — Source data Fig. 4 [file 44319_2025_498_MOESM5_ESM.zip › Figure 4/Figure 4D/Fig.4D_PB2/Mock_001.tif]

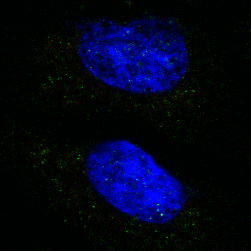

Supplement: Supplementary file 5 — Source data Fig. 4 [file 44319_2025_498_MOESM5_ESM.zip › Figure 4/Figure 4D/Fig.4D_PB2/Mock_001_Crop001.tif]

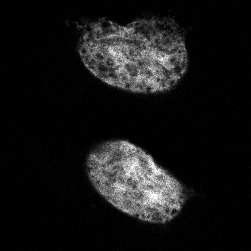

Supplement: Supplementary file 5 — Source data Fig. 4 [file 44319_2025_498_MOESM5_ESM.zip › Figure 4/Figure 4D/Fig.4D_PB2/Mock_001_Crop001_RAW_ch00.tif]

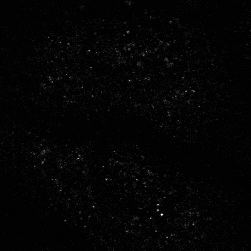

Supplement: Supplementary file 5 — Source data Fig. 4 [file 44319_2025_498_MOESM5_ESM.zip › Figure 4/Figure 4D/Fig.4D_PB2/Mock_001_Crop001_RAW_ch01.tif]

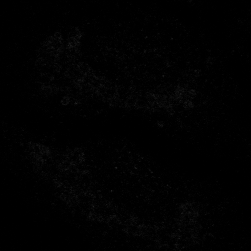

Supplement: Supplementary file 5 — Source data Fig. 4 [file 44319_2025_498_MOESM5_ESM.zip › Figure 4/Figure 4D/Fig.4D_PB2/Mock_001_Crop001_RAW_ch02.tif]

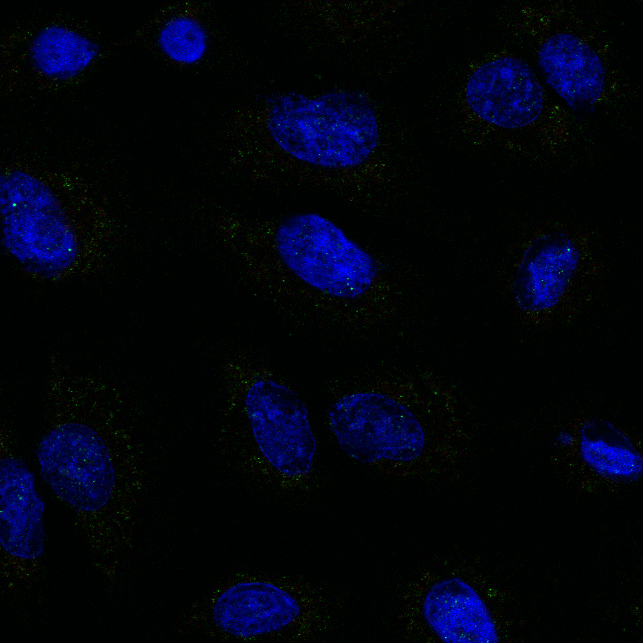

Supplement: Supplementary file 5 — Source data Fig. 4 [file 44319_2025_498_MOESM5_ESM.zip › Figure 4/Figure 4D/Fig.4D_PB2/Mock_001_Crop003.tif]

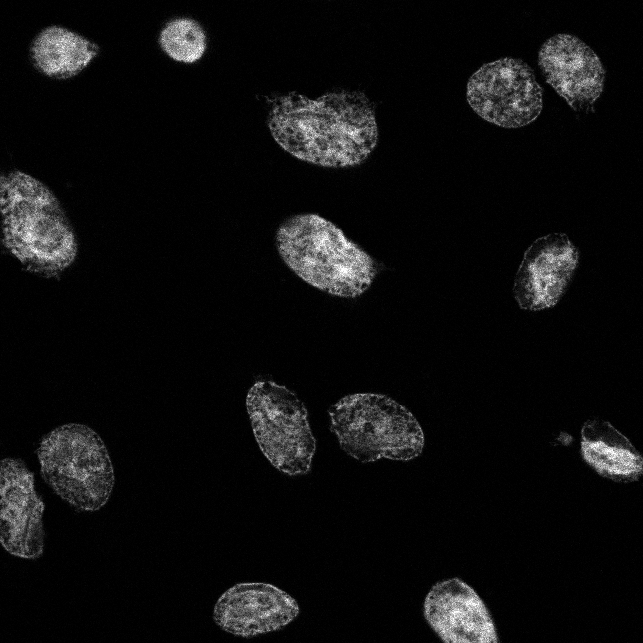

Supplement: Supplementary file 5 — Source data Fig. 4 [file 44319_2025_498_MOESM5_ESM.zip › Figure 4/Figure 4D/Fig.4D_PB2/Mock_001_Crop003_RAW_ch00.tif]

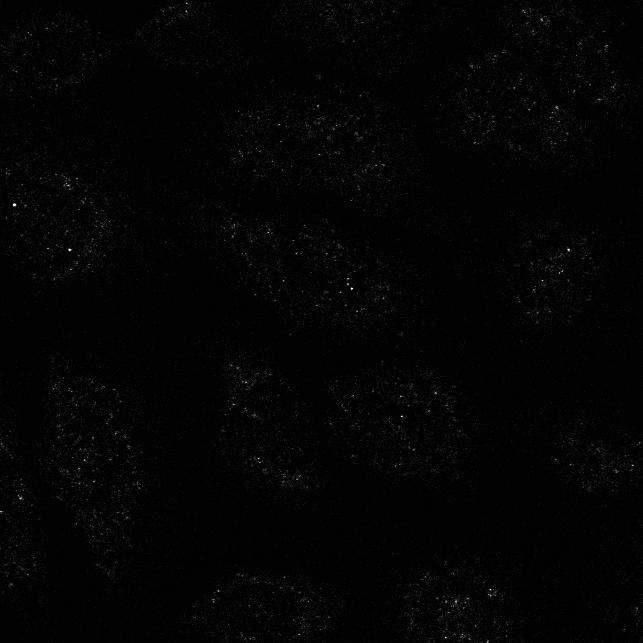

Supplement: Supplementary file 5 — Source data Fig. 4 [file 44319_2025_498_MOESM5_ESM.zip › Figure 4/Figure 4D/Fig.4D_PB2/Mock_001_Crop003_RAW_ch01.tif]

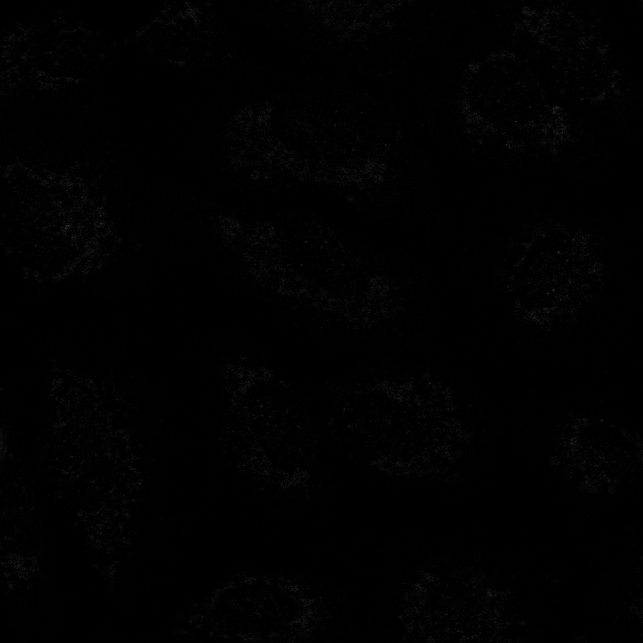

Supplement: Supplementary file 5 — Source data Fig. 4 [file 44319_2025_498_MOESM5_ESM.zip › Figure 4/Figure 4D/Fig.4D_PB2/Mock_001_Crop003_RAW_ch02.tif]

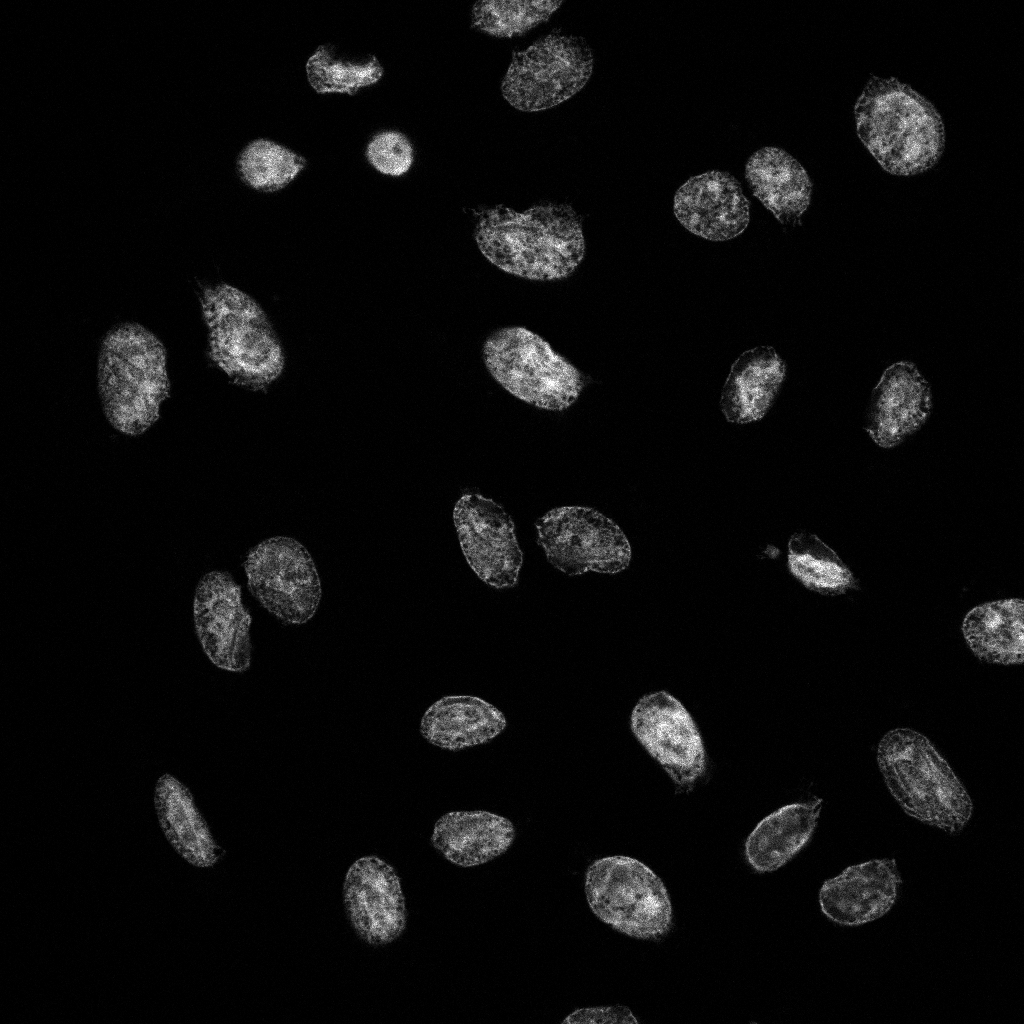

Supplement: Supplementary file 5 — Source data Fig. 4 [file 44319_2025_498_MOESM5_ESM.zip › Figure 4/Figure 4D/Fig.4D_PB2/Mock_001_RAW_ch00.tif]

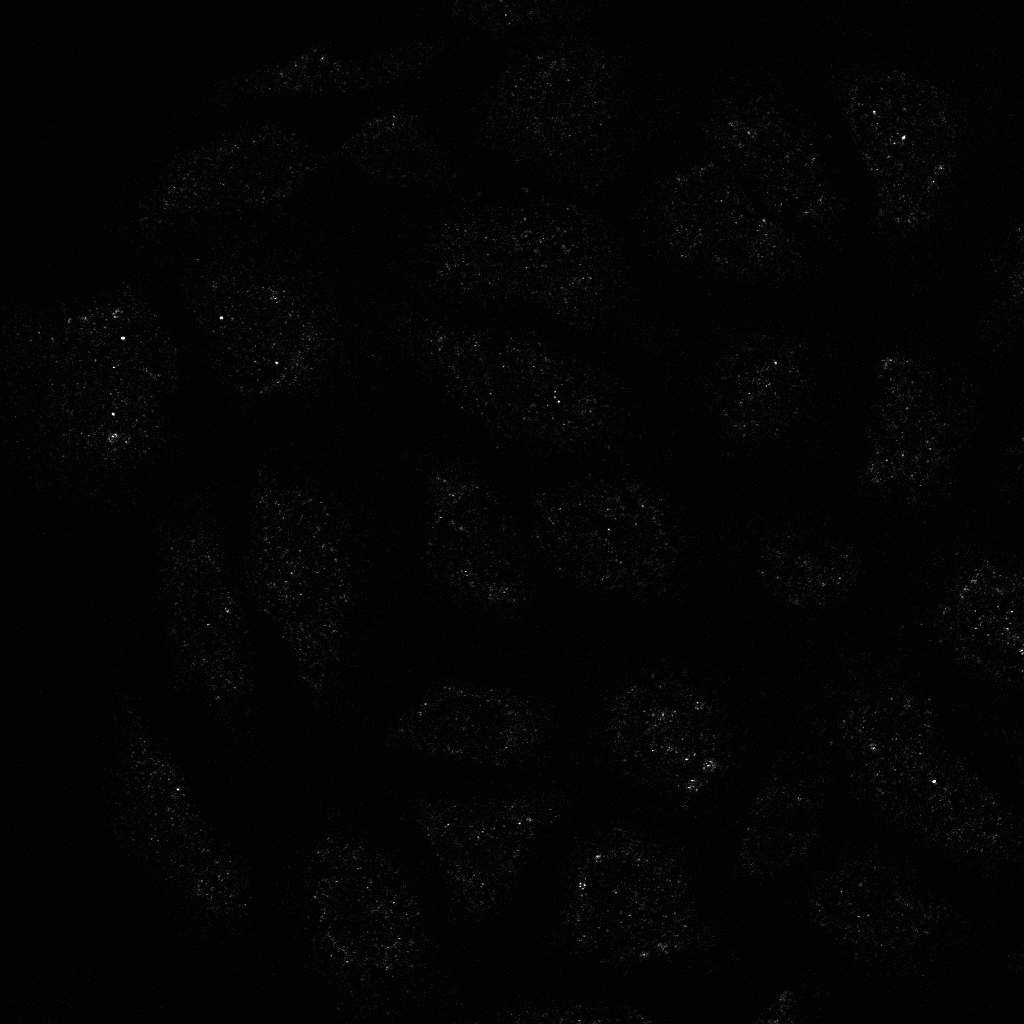

Supplement: Supplementary file 5 — Source data Fig. 4 [file 44319_2025_498_MOESM5_ESM.zip › Figure 4/Figure 4D/Fig.4D_PB2/Mock_001_RAW_ch01.tif]

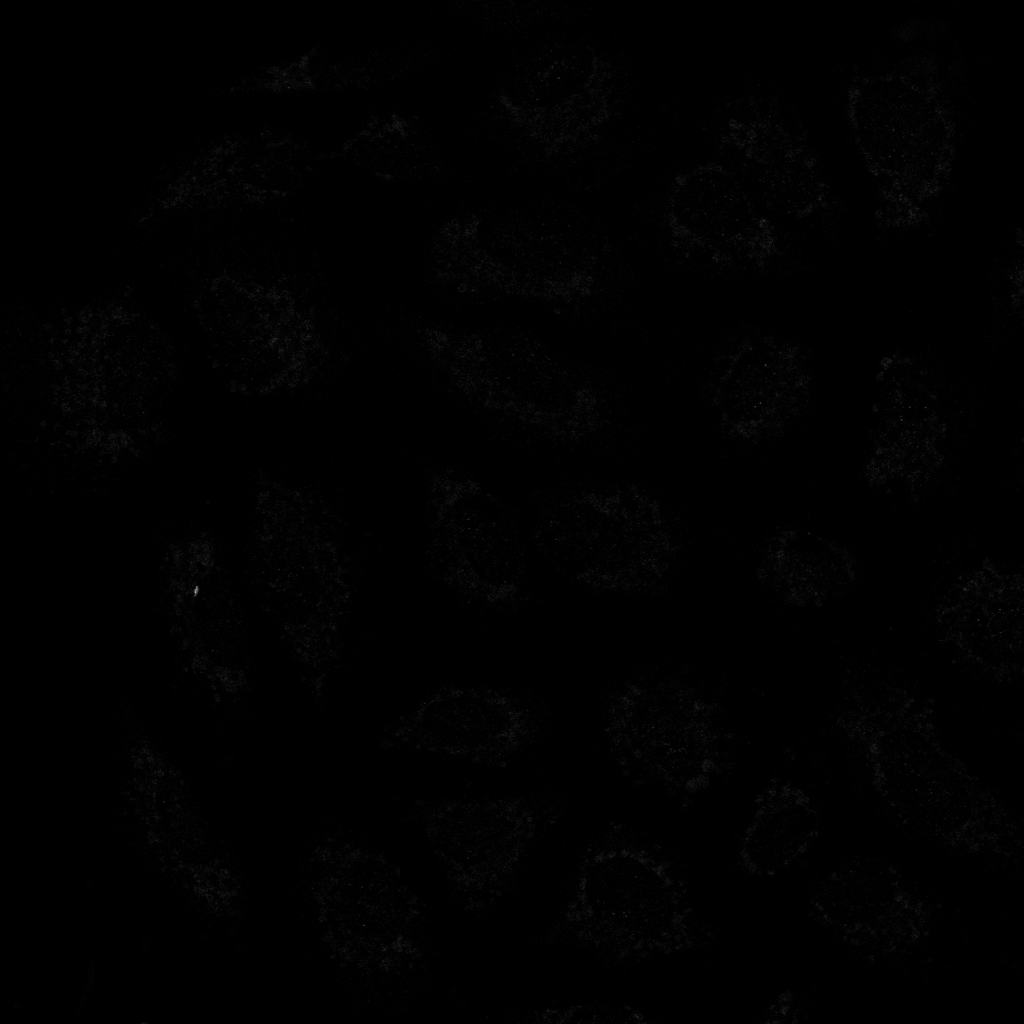

Supplement: Supplementary file 5 — Source data Fig. 4 [file 44319_2025_498_MOESM5_ESM.zip › Figure 4/Figure 4D/Fig.4D_PB2/Mock_001_RAW_ch02.tif]
