# Supplementary material for: Regulated localization of transposable element RNA during influenza A virus infection
Source: EMBO Rep. 2025 Jun 16;26(14):3506–28. doi: 10.1038/s44319-025-00498-2 (PMC12287459; doi:10.1038/s44319-025-00498-2)
Supplement: Supplementary file 6 — Expanded View Figures [file 44319_2025_498_MOESM6_ESM.pdf]

Expanded View Figures

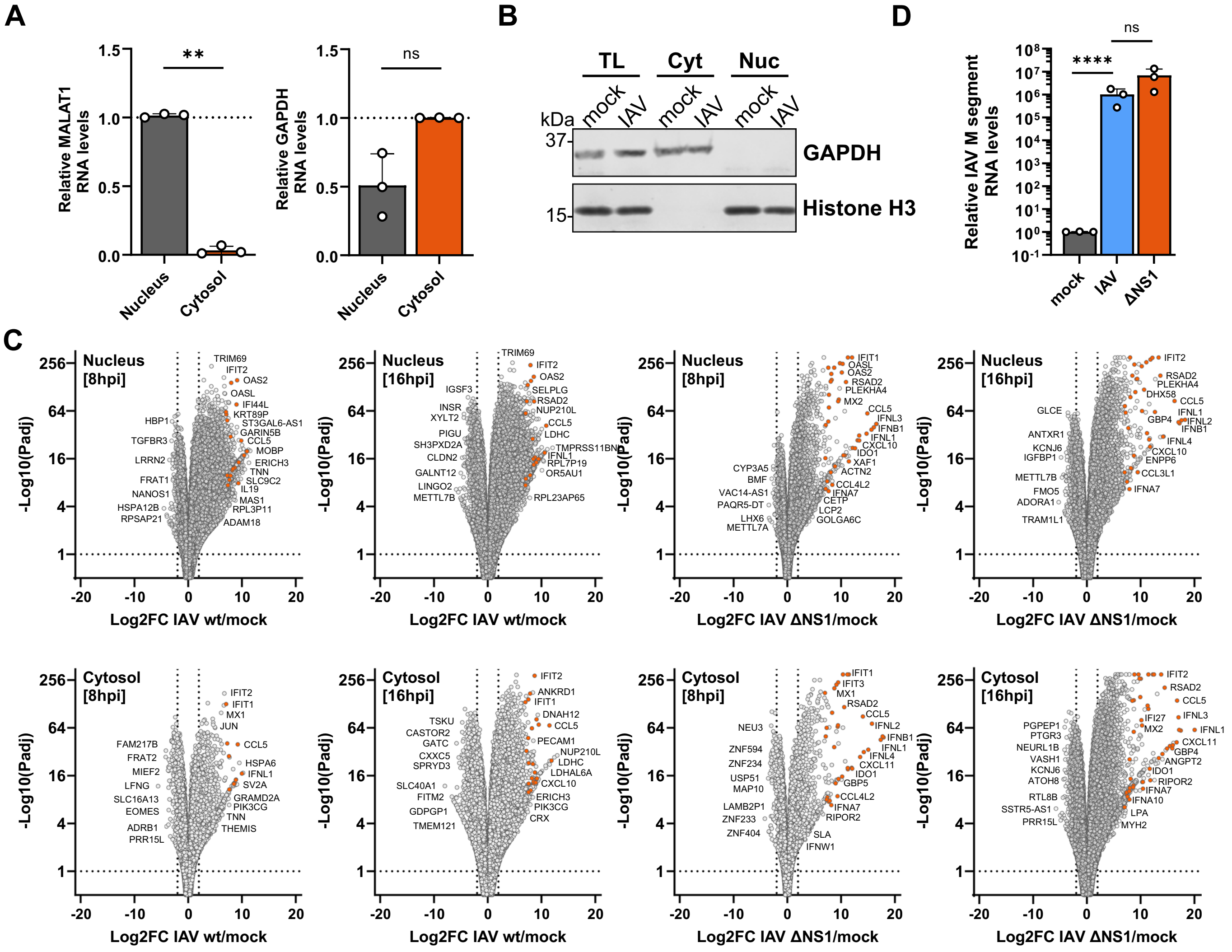

Figure EV1. Fractionation quality controls and subcellular host transcriptome analysis during IAV infection.

(**A**) Subcellular fraction purity assessment: RT-qPCR analysis was conducted on RNA samples from experiments similar to those shown in Fig. 1 with wt IAV. GAPDH was utilized as a cytosolic fraction marker, and MALAT1 served as a nucleus-associated fraction marker. Bars represent mean values and SDs from $n = 3$ biological replicates (each dot corresponds to one replicate). Significance was determined by unpaired *t* test on log-transformed data (**$P = 0.0024$; ns, non-significant). (**B**) Subcellular fraction purity assessment: western blot analysis was conducted on cell lysates from experiments similar to those shown in Fig. 1 with wt IAV, probing for the specific marker proteins GAPDH (cytosolic fraction) and Histone H3 (nucleus-associated fraction). Data are representative of $n = 3$ biological replicates. (**C**) Differential gene expression analysis in subcellular fractions: volcano plots depicting the differential gene expression in A549-ACE2/TMPRSS2 cells following infection with wt IAV or IAV ΔNS1 [MOI = 5 PFU/cell] at the indicated times and in the indicated subcellular fractions. Genes with statistically significant differential expression (P_{adj} <0.1, determined using the Wald test from the DESeq2 package) and a $\log_2$FC > 2 were considered differentially expressed genes (DEGs). Cytokines, chemokines, and interferon-stimulated genes (ISGs) with a $\log_2$FC > 7 and P_{adj} <0.1 are highlighted in orange. Selected gene names are shown. Analysis is based on $n = 3$ biological replicates. See also Dataset EV1. (**D**) Assessment of infection comparability between wt IAV and IAV ΔNS1: A549 cells were infected with wt IAV or IAV ΔNS1 [MOI = 5 PFU/cell] for 16 h. RT-qPCR analysis for IAV M segment was performed on RNA samples. Bars represent mean values and SDs from $n = 3$ biological replicates (each dot corresponds to one replicate). Significance was determined by ordinary one-way ANOVA on log-transformed data (****$P \leq 0.0001$; ns non-significant).

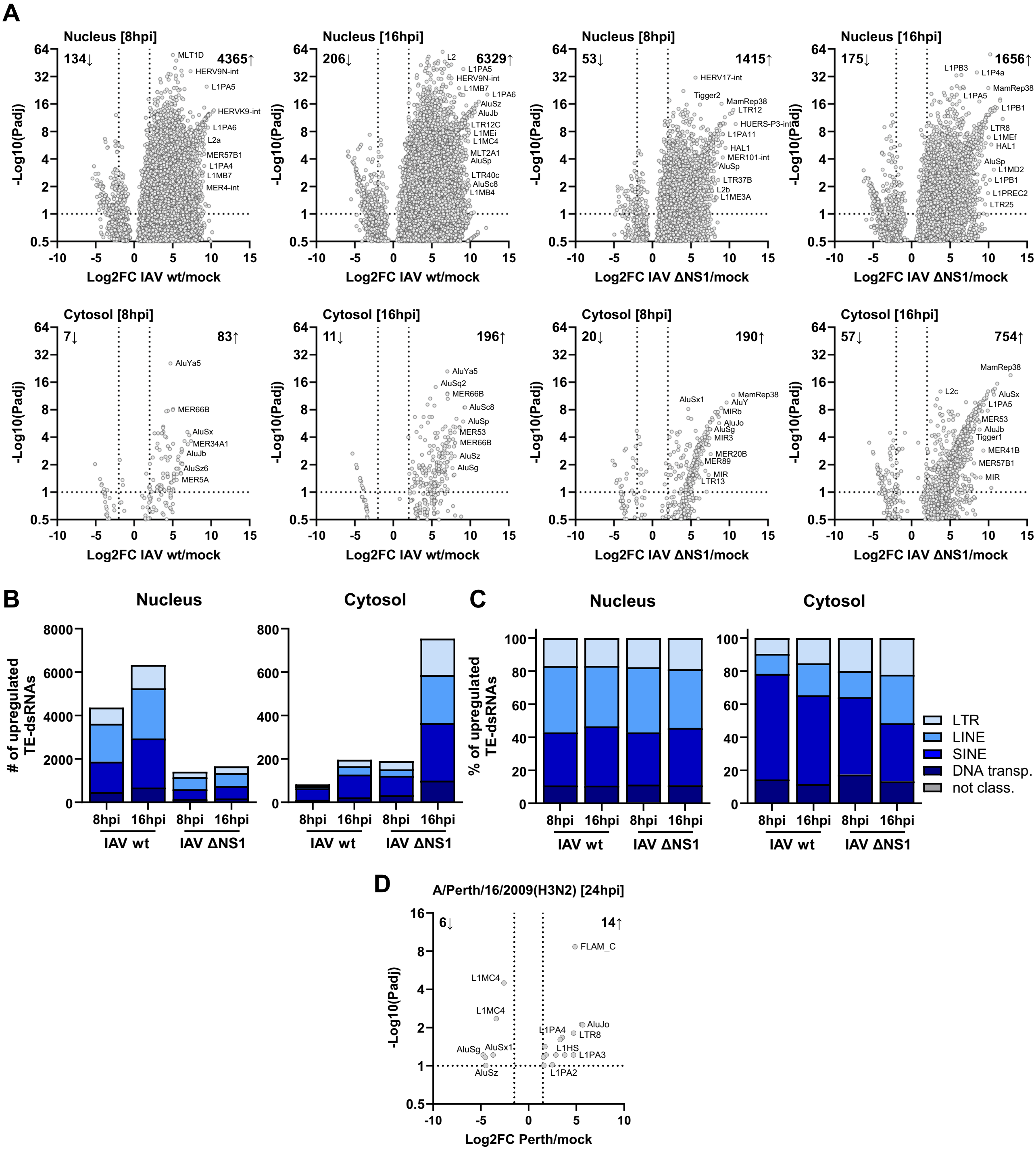
A
Nucleus [8hpi]
134↓
4365↑
MLT1D
HERV9N-int
L1PA5
HERVK9-int
L1PA6
L2a
MER57B1
L1PA4
L1MB7
MER4-int
Nucleus [16hpi]
206↓
6329↑
L2
L1PA5
HERV9N-int
L1MB7
L1PA6
AluSz
AluJb
LTR12C
L1MEi
L1MC4
MLT2A1
AluSp
LTR40c
AluSc8
L1MB4
Nucleus [8hpi]
53↓
1415↑
HERV17-int
Tigger2
MamRep38
LTR12
HUERS-P3-int
L1PA11
HAL1
MER101-int
AluSp
LTR37B
L2b
L1ME3A
Nucleus [16hpi]
175↓
1656↑
L1PB3
L1P4a
MamRep38
L1PA5
L1PB1
LTR8
L1MEf
HAL1
AluSp
L1MD2
L1PB1
L1PREC2
LTR25
Cytosol [8hpi]
7↓
83↑
AluYa5
MER66B
AluSx
MER34A1
AluJb
AluSz6
MER5A
Cytosol [16hpi]
11↓
196↑
AluYa5
AluSq2
MER66B
AluSc8
AluSp
MER53
MER66B
AluSz
AluSg
Cytosol [8hpi]
20↓
190↑
AluSx1
MamRep38
AluY
MIRb
AluJo
AluSg
MIR3
MER20B
MER89
MIR
LTR13
Cytosol [16hpi]
57↓
754↑
MamRep38
L2c
AluSx
L1PA5
MER53
AluJb
Tigger1
MER41B
MER57B1
MIR
-Log10(Padj)
Log2FC IAV wt/mock
Log2FC IAV ΔNS1/mock
B
Nucleus
Cytosol
# of upregulated TE-dsRNAs
8hpi 16hpi 8hpi 16hpi
IAV wt
IAV ΔNS1
C
Nucleus
Cytosol
% of upregulated TE-dsRNAs
LTR
LINE
SINE
DNA transp.
not class.
D
A/Perth/16/2009(H3N2) [24hpi]
6↓
14↑
FLAM_C
L1MC4
L1MC4
AluJo
LTR8
L1PA4
L1HS
L1PA3
AluSg
AluSx1
AluSz
L1PA2
-Log10(Padj)
Log2FC Perth/mock

 © The Author(s)

Figure EV2. Differential expression and classification of TE-dsRNAs in subcellular fractions during IAV infection.

(**A**) Differential expression of TE-dsRNAs in subcellular fractions: volcano plots illustrating the differential expression of TE-dsRNAs in the specified subcellular fractions of A549-ACE2/TMPRSS2 cells following infection with wt IAV or IAV ΔNS1 [MOI = 5 PFU/cell]. TEs reaching the thresholds for statistical significance (P_{adj} <0.1, determined using the Wald test from the DESeq2 package) and $log_2FC > 2$, with bioinformatic evidence for double-strandedness, were considered differentially expressed TE-dsRNAs. Selected element names are shown. Analysis is based on $n = 3$ biological replicates. See also Dataset EV2. (**B**, **C**) Subclasses of significantly upregulated TE-dsRNAs in nuclear and cytosolic fractions: absolute (**B**) and relative (**C**) numbers of TE subclasses significantly upregulated during wt IAV or IAV ΔNS1 infections at the indicated times and compartments are shown for LTR (long terminal repeat retrotransposon), LINE (long interspersed nuclear element), SINE (short interspersed nuclear element) and DNA transposon elements. Analysis is based on $n = 3$ biological replicates. (**D**) Reanalysis of publicly available RNA-Seq data for TE-dsRNAs during IAV infection: volcano plots illustrating the differential expression of TE-dsRNAs in normal human bronchial epithelial cells (BEAS-2B) following infection for 24 h with IAV strain A/Perth/16/2009 (H3N2) [MOI = 1 PFU/cell]. TEs reaching the thresholds for statistical significance (P_{adj} <0.1, determined using the Wald test from the DESeq2 package) and $log_2FC > 1.5$, with bioinformatic evidence for double-strandedness, were considered differentially expressed TE-dsRNAs. Selected element names are shown.

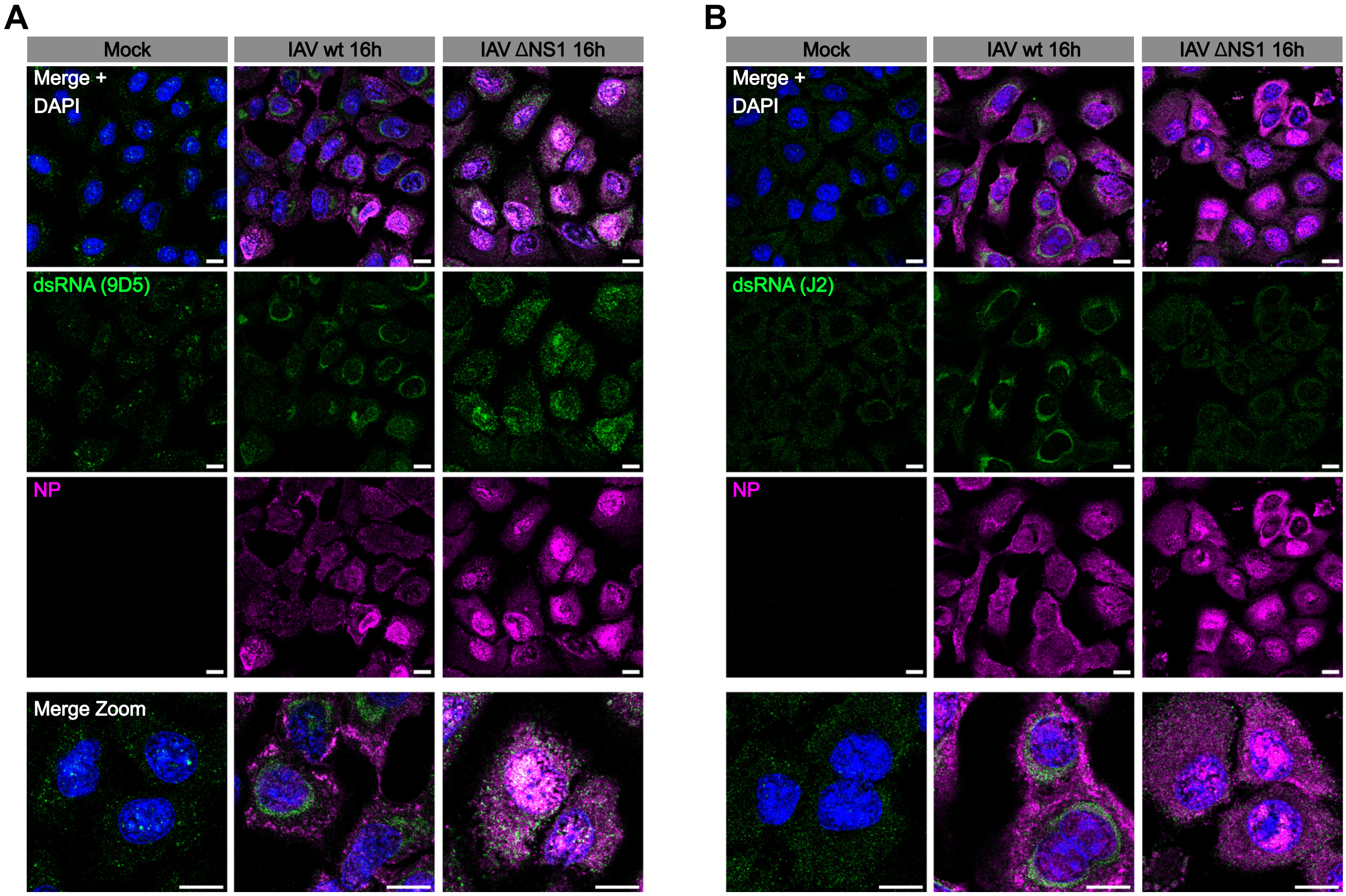

Figure EV3. Immunofluorescence confocal microscopy of total dsRNA in A549 cells infected with wt IAV or IAV ΔNS1.

(**A**, **B**) A549 cells were infected, or mock, with wt IAV or IAV ΔNS1 [MOI = 1 PFU/cell] for 16 h. Subsequently, cells were fixed and permeabilized with methanol, treated with Proteinase K, and stained with specific antibodies against dsRNA (9D5 in (**A**); J2 in (**B**)), the IAV NP protein, and with DAPI to stain nuclei. Immunofluorescence confocal microscopy images are shown, representative of at least $n = 2$ biological replicates. Scale bars represent 10 μm.

 © The Author(s)

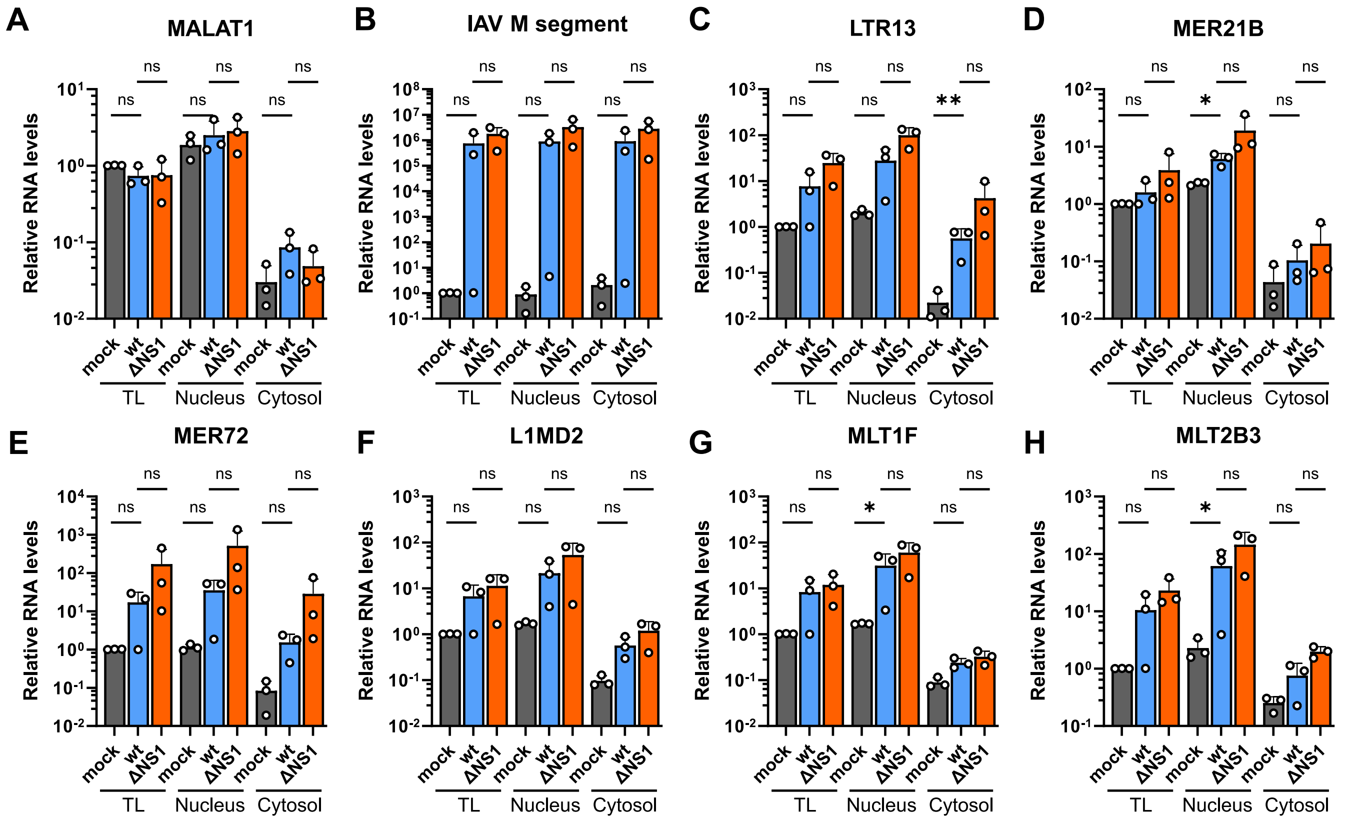

Figure EV4. Nuclear/cytosolic distribution of specific RNAs in MRC5 cells infected with wt IAV or IAV ΔNS1.

(**A–H**) Assessment of TE expression and localization by RT-qPCR: RT-qPCR analysis of selected RNAs in subcellular fractions following mock, wt IAV, or IAV ΔNS1 infections [MOI 5 PFU/cell; 16 h] in MRC5 cells. MALAT1 served as a nuclear-localized fractionation control, IAV M segment served as an infection-level control. Bars represent mean values and SDs from $n = 3$ biological replicates (each dot corresponds to one replicate). Significance was determined by ordinary one-way ANOVA with Šídák's multiple comparisons test on log-transformed data (*$P \leq 0.05$; **$P \leq 0.01$; ns, non-significant).

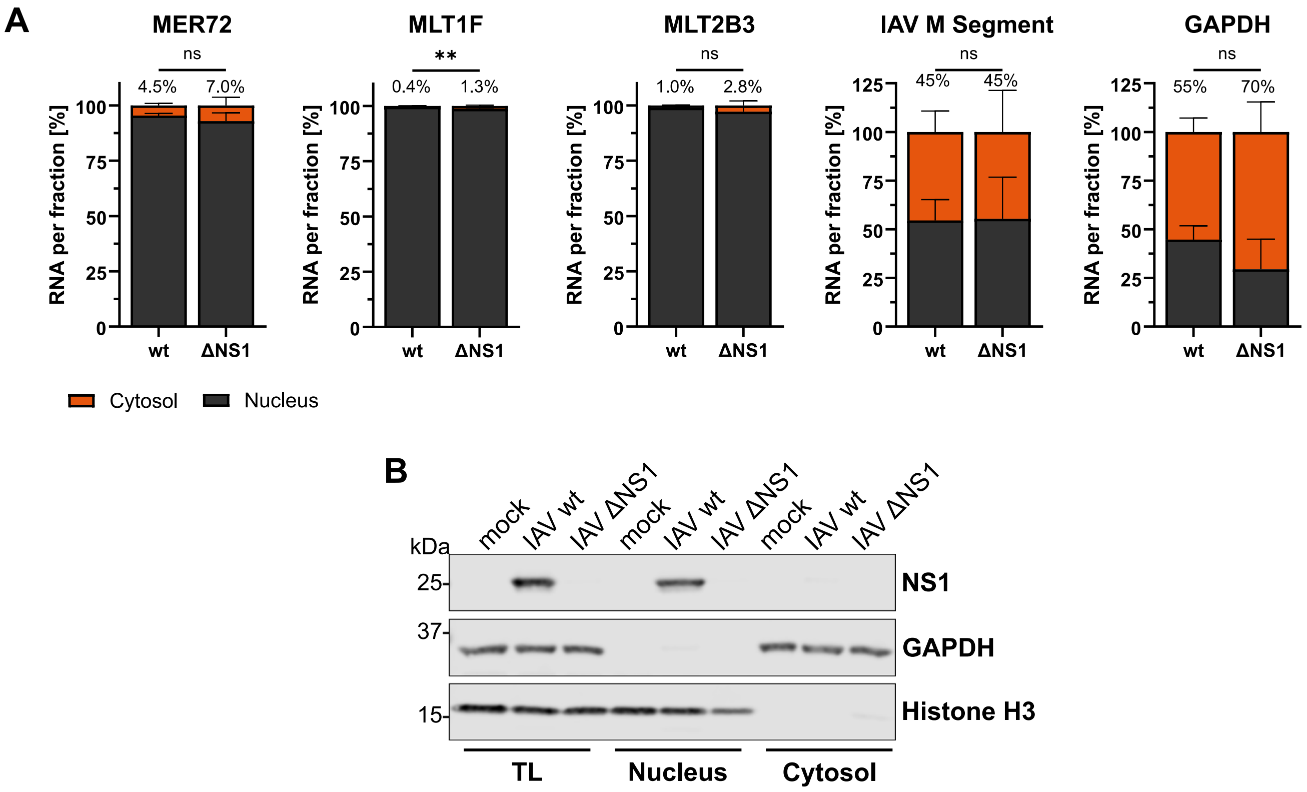

Figure EV5. Relative nuclear/cytosolic distribution of specific RNAs and proteins in A549 cells infected with wt IAV or IAV ΔNS1.

(**A**) RNA expression ratios: RT-qPCR data for selected RNAs from Fig. 3C–J are depicted as the percentage of transcript per fraction. Bars represent mean values and SDs from $n = 4$ biological replicates. The percentages annotated above the bars specifically denote the cytosolic fractions. Significance was determined by unpaired *t* test (**$P \leq 0.0035$; ns non-significant). (**B**) Western blot analysis of IAV NS1 expression in subcellular fractions of A549 cells infected, or mock, with wt IAV or IAV ΔNS1 [MOI = 5 PFU/cell] for 16 h. The purity of subcellular fractions was also assessed by probing for the specific marker proteins GAPDH (cytosolic fraction) and Histone H3 (nucleus-associated fraction). Data are representative of $n = 3$ biological replicates.

 © The Author(s)

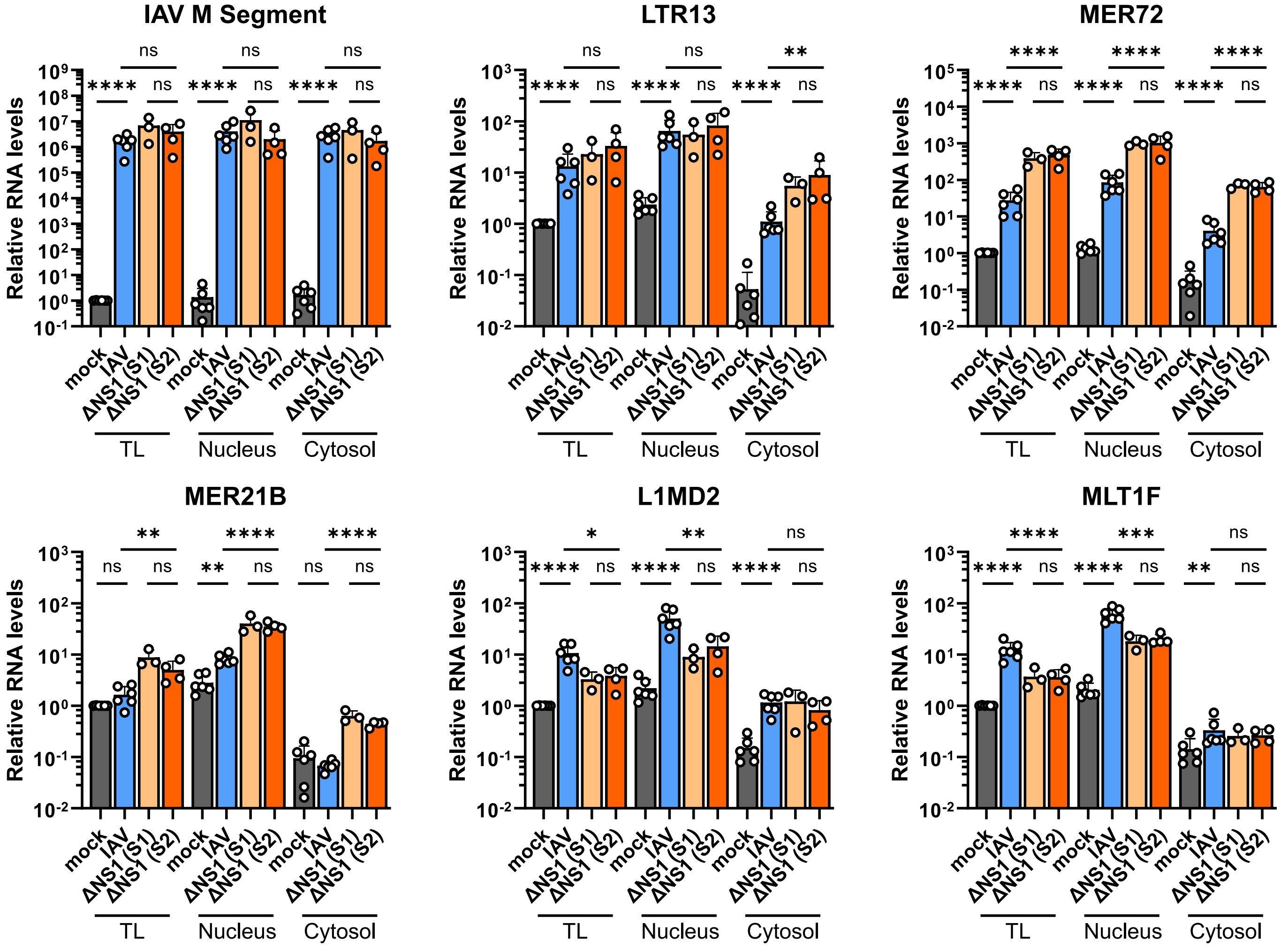

Figure EV6. Validation of differential TE expression during infection of A549 cells with different IAV ΔNS1 stocks.

RT-qPCR analysis to validate the differential expression of selected TEs and viral M RNA in subcellular fractions following mock, wt IAV, IAV ΔNS1 S1, or IAV ΔNS1 S2 infection [MOI = 5 PFU/cell] of A549 cells for 16 h. Following the initial RNA-Seq experiments, we discovered that the IAV ΔNS1 stock used (S1) was inadvertently contaminated with parainfluenza virus 5 (PIV5). A new non-contaminated IAV ΔNS1 stock was therefore prepared (S2) and both stocks were tested for their ability to induce TEs side-by-side. Some data for mock, wt IAV, and IAV ΔNS1 (S2) are also shown in Fig. 3. Bars represent mean values and SDs from at least $n = 3$ biological replicates, each with at least $n = 2$ technical replicates (each dot corresponds to one experiment). Significance was determined by ordinary one-way ANOVA with Šídák's multiple comparisons test on log-transformed data (*$P \leq 0.05$; **$P \leq 0.01$; ***$P \leq 0.001$; ****$P \leq 0.0001$; ns non-significant).
